# Supplementary material for: Abnormal spirometric patterns and respiratory symptoms in HIV patients with no recent pulmonary infection in a periurban hospital in Ghana
Source: PLoS One. 2024 Oct 16;19(10):e0273063. doi: 10.1371/journal.pone.0273063 (PMC11482697; doi:10.1371/journal.pone.0273063)
Supplement: S1 File — (DOCX) [file pone.0273063.s001.docx]

**Supplementary Information**

**Abnormal spirometric patterns and respiratory symptoms in HIV patients with no recent pulmonary infection in a periurban hospital in Ghana**

Kwame Yeboah^1^, Latif Musa^1,2^, Kweku Bedu-Addo^2^

1. Department of Physiology, University of Ghana Medical School, Accra, Ghana
2. Department of Physiology, School of Medicine and Dentistry, KNUST, Kumasi, Ghana

Table S1. Factors associated with obstructive lung pattern in cART-treated HIV patients

|  | | Unadjusted model | | Adjusted model | |
| --- | --- | --- | --- | --- | --- |
|  | | OR (95% CI) | p | OR (95% CI) | p |
| Age | | 1.05 (1.01 – 1.1) | 0.028 | 1.07 (1 – 1.14) | 0.06 |
| Females | | 2.2 (0.48 – 10.19) | 0.312 | 7.83 (0.94 – 18.92) | 0.059 |
| Unmarried | | 1.29 (0.45 – 3.75) | 0.638 | 1.19 (0.33 – 4.33) | 0.791 |
| Employment | |  |  |  |  |
|  | Self-employed | 0.59 (0.19 – 1.73) | 0.319 | 0.55 (0.14 – 2.15) | 0.389 |
|  | Unemployed | 1.17 (0.22 – 6.2) | 0.856 | 1.36 (0.17 – 11.21) | 0.774 |
| Medium/high biomass exposure | | 1.64 (0.56 – 4.83) | 0.367 | 3.3 (0.71 – 15.26) | 0.127 |
| BMI (unit change) | | 0.93 (0.83 – 1.05) | 0.244 | 0.93 (0.81 – 1.06) | 0.287 |
| Respiratory symptoms | | 1.92 (0.66 – 5.56) | 0.23 | 1.55 (0.41 – 5.94) | 0.524 |
| Alcohol intake | | 0.64 (0.14 – 2.98) | 0.567 | 0.89 (0.13 – 6.19) | 0.904 |
| Current smoking | | 2.39 (0.46 – 12.39) | 0.298 | 1.95 (0.21 – 17.8) | 0.556 |
| Duration of infection, per year | | 1.08 (0.96 – 1.22) | 0.191 | 1.02 (0.89 – 1.17) | 0.802 |
| Duration of treatment, per year | | 1.04 (0.92 – 1.16) | 0.534 |  |  |
| CD4 count, per unit increase | | 0.82 (0.55 – 1.22) | 0.336 |  |  |

cART, combination antiretroviral therapy; JHS, junior high school; SHS, senior high school; BMI, body mass index.

Table S2. Factors associated with obstructive lung pattern in cART-naïve HIV patients

|  | | Unadjusted model | | Adjusted model | |
| --- | --- | --- | --- | --- | --- |
|  | | OR (95% CI) | p | OR (95% CI) | p |
| Age | | 0.97 (0.92 – 1.02) | 0.28 | 0.94 (0.86 – 1.03) | 0.162 |
| **Females** | | **5.33 (1.15 – 24.74)** | **0.032** | **8.04 (1.08 – 29.59)** | **0.042** |
| Employment | |  |  |  |  |
|  | Self-employed | 2.25 (0.6 – 8.4) | 0.277 | 2.02 (0.4 – 10.34) | 0.398 |
|  | Unemployed | 4.5 (1.02 – 19.93) | 0.048 | 5.88 (0.72 – 48.41) | 0.099 |
| Education: reference= tertiary | | |  |  |  |
|  | None | 1.17 (0.19 – 7.25) | 0.869 |  |  |
|  | Basic/JHS | 0.57 (0.15 – 2.16) | 0.407 |  |  |
|  | SHS/Tech | 0.64 (0.11 – 3.8) | 0.62 |  |  |
| **Medium/high biomass exposure** | | **2.61 (0.85 – 7.97)** | **0.092** | **11.88 (2.03 – 27.52)** | **0.006** |
| BMI (unit change) | | 0.74 (0.61 – 0.91) | 0.003 |  |  |
| **Respiratory symptoms** | | **11 (2.36 – 51.19)** | **0.002** | **15.03 (2.5 – 30.22)** | **0.003** |
| CD4 count, per unit increase | | 0.92 (0.54 – 1.59) | 0.771 | 0.96 (0.33 – 2.78) | 0.947 |

JHS, junior high school; SHS, senior high school; BMI, body mass index.

Table S3. Factors associated with obstructive lung pattern in non-HIV controls

|  | | Unadjusted model | | Adjusted model | |
| --- | --- | --- | --- | --- | --- |
|  | | OR (95% CI) | p | OR (95% CI) | p |
| **Age** | | **0.92 (0.85 – 0.99)** | **0.035** | **0.9 (0.81 – 1)** | **0.048** |
| Females | | 3.06 (0.66 – 14.24) | 0.153 | 2.65 (0.53 – 13.24) | 0.234 |
| Unmarried | | 2.17 (0.65 – 7.25) | 0.208 | 1.11 (0.23 – 5.29 | 0.9 |
| Employment | |  |  |  |  |
|  | Self-employed | 2.33 (0.44 – 12.27) | 0.317 |  |  |
|  | Unemployed | 3.11 (0.85 – 11.41) | 0.087 |  |  |
| Medium/high biomass exposure | | 1.1 (0.23 – 5.26) | 0.905 |  |  |
| BMI (unit change) | | 1.06 (0.95 – 1.19) | 0.318 | 1.1 (0.96 – 1.27) | 0.179 |
| Respiratory symptoms | | 0.68 (0.14 – 3.21) | 0.625 | 0.7 (0.13 – 3.77) | 0.677 |
| Alcohol intake | | 1.27 (0.37 – 4.31) | 0.701 | 1.32 (0.34 – 5.1) | 0.689 |

Table S4. Factors associated with restrictive lung pattern in HAART-treated HIV patients

|  | | Unadjusted model | | Adjusted model | |
| --- | --- | --- | --- | --- | --- |
|  | | OR (95% CI) | p | OR (95% CI) | p |
| **Age** | | **0.95 (0.92 – 0.98)** | **0.002** | **0.92 (0.87 – 0.96)** | **<0.001** |
| Females | | 3.24 (1.26 – 8.38) | 0015 | 2.62 (0.68 – 10.11) | 0.161 |
| **Unmarried** | | **2.75 (1.4 – 5.41)** | **0.003** | **7.3 (2.86 – 18.66)** | **<0.001** |
| Employment | |  |  |  |  |
|  | Self-employed | 1.06 (0.52 – 2.15) | 0.873 | 0.75 (0.31 – 1.82) | 0.529 |
|  | Unemployed | 2.93 (0.9 – 9.58) | 0.075 | 7.92 (1.36 – 26.14) | 0.021 |
| **Medium/high biomass exposure** | | **3.04 (1.3 – 7.14)** | **0.01** | **5.81 (1.71 – 19.72)** | **0.005** |
| BMI (unit change) | | 1 (0.94 – 1.06) |  | 0.95 (0.87 – 1.04) | 0.286 |
| BMI categories | |  |  |  |  |
|  | Underweight | 1.04 (0.29 – 3.8) | 0.951 |  |  |
|  | Overweight | 1.1 (0.52 – 2.34) | 0.798 |  |  |
|  | Obese | 1.39 (0.5 – 3.85) | 0.527 |  |  |
| Respiratory symptoms | | 0.64 (0.33 – 1.24) | 0.183 | 0.91 (0.37 – 2.26) | 0.846 |
| Alcohol intake | | 0.47 (0.18 – 1.23) | 0.123 | 0.4 (0.11 – 1.39) | 0.149 |
| Current smoking | | 0.46 (0.1 – 2.25) | 0.339 | 0.55 (0.1 – 2.78) | 0.539 |
| Duration of infection, per year | | 0.87 (0.8 – 0.94) | <0.001 |  |  |
| Duration of treatment, per year | | 0.86 (0.79 – 0.93) | <0.001 |  |  |
| CD4 count, per cubic root of unit increase | | 1.19 (0.94 – 1.52) | 0.155 | 1.03 (0.71 – 1.49) | 0.895 |

Table S5. Factors associated with restrictive lung pattern in HAART-naïve HIV patients

|  | | Unadjusted model | | Adjusted model | |
| --- | --- | --- | --- | --- | --- |
|  | | OR (95% CI) | p | OR (95% CI) | p |
| Age | | 1.01 (0.98 – 1.04) | 0.575 | 1.05 (1 – 1.1) | 0.051 |
| **Females** | | **3.25 (1.36 – 7.77)** | **0.008** | **3.24 (1.06 – 9.97)** | **0.041** |
| Unmarried | | 1.95 (0.86 – 4.44) | 0.112 | 1.78 (0.63 – 5.04) | 0.279 |
| **Medium/high biomass exposure** | | **3.29 (1.27 – 8.57)** | **0.015** | **3.22 (1.06 – 9.8)** | **0.039** |
| BMI (unit change) | | 0.99 (0.91 – 1.08) | 0.85 | 0.98 (0.88 – 1.09) | 0.685 |
| BMI categories | |  |  |  |  |
|  | Underweight | 0.41 (0.09 – 1.92) | 0.257 |  |  |
|  | Overweight | 1.23 (0.43 – 3.52) | 0.703 |  |  |
|  | Obese | 1.09 (0.32 – 3.73) | 0.89 |  |  |
| Respiratory symptoms | | 1.46 (0.67 – 3.14) | 0.341 | 1.16 (0.47 – 2.86) | 0.754 |
| **Alcohol intake** | | **2.09 (0.91 – 4.82)** | **0.083** | **3.36 (1.21 – 9.32)** | **0.02** |
| Current smoking | |  |  |  |  |
| CD4 count, per cubic root of unit increase | | 1.07 (0.73 – 1.58) | 0.722 | 1.27 (0.76 – 2.13) | 0.365 |

Table S6. Factors associated with restrictive lung pattern in non-HIV controls

|  | | Unadjusted model | | Adjusted model | |
| --- | --- | --- | --- | --- | --- |
|  | | OR (95% CI) | p | OR (95% CI) | p |
| Age | | 0.99 (0.96 – 1.01) | 0.312 | 0.99 (0.94 – 1.03) | 0.536 |
| Females | | 0.74 (0.33 – 1.66) | 0.46 | 0.8 (0.29 – 2.19) | 0.658 |
| Unmarried | | 0.77 (0.35 – 1.68) | 0.514 | 2.43 (0.97 – 1.23) | 0.129 |
| Employment | |  |  |  |  |
|  | Self-employed | 1.78 (0.51 – 6.21) | 0.364 | 6 (0.56 – 64.04) | 0.38 |
|  | Unemployed | 1.67 (0.59 – 4.76) | 0.336 | 4.78 (1.03 – 22.24) | 0.046 |
| Education: reference= tertiary | | |  |  |  |
|  | None | 3 (0.4 – 22.38) | 0.284 | 0.51 (0.02 – 12.18) | 0.677 |
|  | Basic/JHS | 0.27 (0.06 – 1.24) | 0.093 | 0.15 (0.01 – 1.93) | 0.145 |
|  | **SHS/Tech** | **0.27 (0.06 – 1.24)** | **0.093** | **0.14 (0.02 – 0.94)** | **0.043** |
| Medium/high biomass exposure | | 3.24 (0.72 – 14.55) | 0.126 | 1.13 (0.08 – 15.61) | 0.929 |
| BMI (unit change) | | 0.96 (0.88 – 1.04) | 0.329 | 0.92 (0.82 – 1.03) | 0.156 |
| Respiratory symptoms | | 0.54 (0.17 – 1.67) | 0.284 | 0.44 (0.11 – 1.74) | 0.24 |
| Alcohol intake | | 3.24 (1.42 – 7.42) | 0.005 |  |  |

Table S7. Respiratory symptoms and spirometric abnormalities among HAART-treated HIV patients

|  | | AZT/3TC/NVP or EFV (n=52) | TDF/3TC/NVP or EFV (n=94) | LPV/r-based treatment (n=12) | p |
| --- | --- | --- | --- | --- | --- |
| Obstructive pattern | | 2 (3.8) | 12 (12.8) | 2 (16.7) | 0.171 |
| Restrictive pattern | | 20 (38.5) | 44 (46.8) | 4 (33.3) | 0.484 |
| Any respiratory symptom | | 20 (38.5) | 48 (51.1) | 8 (66.7) | 0.141 |
|  | Cough | 16 (30.8) | 28 (29.8) | 4 (33.3) | 0.966 |
|  | Phlegm | 14 (26.9) | 40 (42.6) | 2 (16.7) | 0.062 |
|  | **Wheeze** | **2 (3.8)** | **16 (17)** | **4 (33.3)** | **0.012** |
|  | **Dyspnoea** | **6 (11.5)** | **14 (14.9)** | **6 (50)** | **0.004** |

AZT, zidovudine; 3TC, lamivudine; NVP, nevirapine; TDF, tenofovir; EFV, efavirenz; LPV/r, lopinavir/ritonavir, ABC, abacavir
