# Supplementary material for: Abnormal spirometric patterns and respiratory symptoms in HIV patients with no recent pulmonary infection in a periurban hospital in Ghana
Source: PLoS One. 2024 Oct 16;19(10):e0273063. doi: 10.1371/journal.pone.0273063 (PMC11482697; doi:10.1371/journal.pone.0273063)
Supplement: Attachment — Submitted filename: PONE-D-22-21464_R1_Annotated.pdf [file pone.0273063.s002.pdf]

PLOS ONE

Abnormal spirometric patterns and respiratory symptoms in HIV patients with no recent pulmonary infection in a periurban hospital in Ghana

--Manuscript Draft--

**Manuscript Number:**	PONE-D-22-21464R1
**Article Type:**	Research Article
**Full Title:**	Abnormal spirometric patterns and respiratory symptoms in HIV patients with no recent pulmonary infection in a periurban hospital in Ghana
**Short Title:**	Abnormal spirometric patterns and respiratory symptoms in HIV patients
**Corresponding Author:**	Kwame Yeboah, PhD<br>University of Ghana<br>Legon, Greater Accra GHANA
**Keywords:**	Abnormal spirometric patterns, Respiratory symptoms, HIV, HAART, Ghana
**Abstract:**	Background: Human immunodeficiency virus (HIV) infection is associated with chronic airway obstruction, even in those with viral suppression by combination antiretroviral treatment (cART). Spirometry is a supplementary test that aids in diagnosing pulmonary dysfunction in people living with HIV.<br>Aim: To compare the prevalence of spirometric abnormalities among cART-treated HIV patients and cART-naïve HIV patients with non-HIV controls with no recent history of pulmonary infection in a peri-urban hospital in Ghana.<br>Methods: In a case-control design, we recruited 158 cART-treated HIV patients, 150 cART-naïve HIV patients and 156 non-HIV controls for the study. Clinical, sociodemographic data and respiratory symptoms were collected using a structured questionnaire. Spirometric abnormalities were categorised as obstructive (OSP) or restrictive (RSP) spirometric patterns based on the Cameroonian reference equation.<br>Results: The prevalence of OSP was higher in the cART-treated and cART-naïve HIV patients compared to non-HIV controls (13.9% vs 10.7% vs 5.1% respectively, p=0.026), whereas that of RSP was similar among the study groups. Respiratory symptoms were common among cART-treated and cART-naïve HIV patients compared to non-HIV controls (48.1% vs 40% vs 19.2% respectively, p<0.001). The major determinants of OSP were female gender [OR (95% CI)=2.46 (1.09 – 5.13), p=0.031], former cigarette smoking [1.92 (1.04 – 3.89), p<0.001] exposure to medium-to-high levels of biomass [3.07 (1.16 – 8.73), p=0.019], presence of a respiratory symptom [1.89 (1.11 – 5.08), p=0.029] and unemployment [3.26 (1.19 – 8.95), p=0.042], and that of RSP were age, female gender [1.74 (1.05 – 4.29), p=0.041], former cigarette smoking [2.31 (1.27 – 6.77), p<0.001], medium-to-high biomass exposure [1.58 (1.06 – 5.37), p=0.043].<br>Conclusion: In HIV patients without any recent pulmonary infection in a peri-urban area of Ghana, there was a higher prevalence of OSP among cART-treated and cART naïve HIV patients compared to the non-HIV control. However, the prevalence of RSP was similar among various study groups.
**Order of Authors:**	Kwame Yeboah
	Latif Musah, MPhil
	Kweku Bedu-Addo, PhD
**Opposed Reviewers:**	
**Response to Reviewers:**	5. Review Comments to the Author<br><br>Reviewer #1: The authors address the issue of a comorbidity that often goes unnoticed.<br>1. The introduction is well developed.<br>2. In the M&M section there are some aspects that should be taken into account:<br>- Define better the inclusion and exclusion criteria of this proposal- Use (or not) of bronchodilators, previous history of tuberculosis, previous history of AIDS events, etc.<br>Response: We have included the eligibility criteria in the methodology section.

Powered by Editorial Manager® and ProduXion Manager® from Aries Systems Corporation

	- Please define former smoker. Please, could you provide information on the number of pack-year?<br>Response: Most of our former smokers were occasional smokers who used to smoke during ceremonies or with friends. Most of them found it difficult to recollect the estimated frequency and number of sticks they smoked previously. That is the reason we chose to analyze only current smoking to avoid high levels of recall bias.<br><br>- In PLWH on ART, how many had an undetectable viral load (define)? How long have they been on ART?<br>Response: Due to logistical constraints at the time of data collection, viral load measurement in southern Ghana was temporarily suspended and when they resume measurement, there was a long queue of samples to be analyzed. This hampered our ability to measure viral load. We have therefore acknowledged this in the limitation section.<br><br>- Could you provide information about physical activity? It has been implicated on lung health.<br>Response: We did not collect any data on physical activity levels. We have acknowledged this in our limitation section.<br><br>- Please assess FEV1/FVC ratio 80%.<br>Response: We have provided assessments of abnormal spirometric measurements based on the fixed ratio in Table 1.<br><br>- Prior to multivariate analysis it would be advisable to do a collinearity analysis.<br>Response: We performed collinearity analysis and in all cases, valence inflation factor was between 1.07 – 1.51 and tolerance was 0.71 – 0.96. Also, we excluded CD4 cell counts, HIV duration and cART drugs due to multicollinearity with other factors in the models.<br><br>3. The results and discussion section could be conditioned by the above questions.<br>Reviewer #2: Reviewer's Comments<br>Abstract<br>The authors should state what odd ratios and the p values were with regards to the major determinants of OSP and RSP.<br>Response: We have kindly provided the odds ratios and p-values in the abstract section.<br><br>Sample Size<br>Why was a prevalence study in Uganda used for the calculation of the sample size?<br>Response: We thank the reviewer for drawing our attention to this. We have corrected the sample size calculation to include two studies, one in HIV patients and the other in non-HIV population, conducted in rural Ugandan population. We believe that since lung abnormalities are related to urbanization, the rural setting of the Uganda studies closely resemble our study setting. Hence, that informed us to use the data from rural Uganda.<br><br>Results<br>Table 1<br>Past smoking looks like a predisposing factor for the abnormalities seen in the spirometry when the HIV patients are compared to the non-HIV patients. This looks like the major factor to account for the results that was gotten. How did the authors control for this since it seems to be a major confounding factor?<br>Response: We have kindly included previous smoking in our revised analysis.<br><br>Determinants of abnormal spirometry patterns<br>The authors found the female gender, unemployment, underweight to be associated with increased odds of having OSP. There were no explanations for these findings in the results section.<br>The authors also found female gender, being unmarried, being self-employed or unemployed to be associated with increased odds of restrictive lung pattern. However, there are no explanations for these findings in their results. Can they explain the association between these variables and the spirometry abnormalities.<br>Response: We have kindly expanded the discussion section to include these

Powered by Editorial Manager® and ProduXion Manager® from Aries Systems Corporation

determinants of abnormal spirometric patterns in the “Determinants of abnormal spirometric patterns” section of the discussion.
Reviewer #3: 1. Study title: is satisfactory

2. Abstract: is satisfactory but change the sentence : the Spirometry is an accurate method of diagnosing pulmonary dysfunction n people living with HIV (PLWH). Spirometry is a supplementary test that aids in the diagnosis a pulmonary disease
Response: We have kindly revised the abstract according to the recommendations of the reviewers.

Introduction : What is the rational for this study and clinical importance of the study? After the hypothesis , please restate the objective of your study
Response: We hae restated the objective after the rationale of the study.

Methodology
The definition of biomass use/exposure and sentence has no reference, the author should include a reference(s) -Medium-high biomass exposure was defined as those who reported using firewood, sawdust, cow-dung or corn cubs as a fuel source for work or cooking at home on most days
Response: We have kindly included reference to that statement and adjusted the statement to reflect that reference.
The author did not mention how the modified version of the American Thoracic Society respiratory questionnaire was administered, and whether it was in English or Local dialect, If in local dialect was the questionnaire validated.
Response: For the majority of the participants, they could comprehend basic English and hence, the questionnaire was administered in English. However, there were few participants with limited English conprehension that we translated the questionnaire into their local dialect before administration. The questionnaire, unfortunately, had not been validated. However, the symptoms being asked from the questionnaire are similar to what we normally ask the patients during routine clinical visits and hence, we expected them to understand.
what is reason for not collecting CD4 Count in the non HIV controls?
Response: We could not measure CD4 lymphocyte counts in non-HIV participants due to logistical constraints. There was a lot of pressure on the only device to assay CD4 cell count at the region where the study was conducted, with large quantities of samples and fewer reagents available. Therefore, we could only lobby the needed authorities for the assaying of only registered as HIV positive.

Is there any reason for using lower limit of normal than the fixed ratio of FEV/FVC
Response: We have presented both the results for the lower limit of normal and fixed ratios in the revise manuscript.

Sample size calculation lacks clarity, please include the sample size calculation with relevant reference. How did you arrived at the selected figures for the cases and controls
Response: We have kindly provided further information of the sample size calculation.

Results

The author needs to tell the reader how many where approached in both the cases and control , the attrition rate in each group and the reason for not completing the study. How many patients and controls had poor spirometry results and was there problem encountered during questionnaire administration?

Response: we have kindly provided information on the number of participants initially recruited and those who were not able to produce the required manoeuvre for accurate spirometric recordings.

Table 1 - the unit of the parameter should be in closed bracket
Response: We kindly prefer to use commas for units of continuous variables and counts (percentages) for categorical variables.

Table 2- There are 2 respiratory symptoms parameters with different results, the author should verify these results.

	Response: We have humbly removed the wrong data from the table.<br><br><br>Discussion<br>It is not a robust discussion, there were not enough explanation for incongruent result when making comparison. This sentence was not fully discussed and interpreted : Drummond et al reported in HIV patients in the United States that, out of 37% of HIV patients who had abnormal spirometry, 10% had RSP while 27% had OSP [8], a pattern contrary to what was observed in our study population with RSP higher than OSP.<br>Response: We have kindly rephrased the sentence to make it clearer and we have explained the sentence.<br><br>What is the reason for higher number of smoker in HIV? I would suggest you look for previous work on smoking in HIV in Sub-Saharan Africa to support your discussion.<br><br>Response: We have explained that the HIV patients who smoked were more likely to be from low socioeconomic status and we have also included participants who were former smokers in the multivariable analysis.<br><br>Regarding the respiratory symptoms , the Nigerian study you cited had an objective and design that were different from this study . I would suggest similar study below in Nigeria with almost similar methods to your study to support your discussion- pls note it is at your discretion<br><br>Onyedum, C. C., Chukwuka, J. C., Onwubere, B. J., Ulasi, I. I., & Onwuekwe, I. O. (2010). Respiratory symptoms and ventilatory function tests in Nigerians with HIV infection. African health sciences, 10(2), 130–137.<br><br>response: we appreciate the recommendation by the reviewer and have done so<br><br>The High prevalence respiratory symptom in HIV , Is there a relation between gender and, high biomass exposure?<br>Response: We have kindly modified that statement in the discussion section to reflect what is in the revised results. Only HIV status and high biomass exposure were associated with respiratory symptoms.<br><br>There discussion regarding these association should be more vibrant<br><br>It has been that about 20% and 28% of patients initially diagnosed with OSP reversed to normal pattern after one and two years, respectively. Pls reword this sentence for clarity and adduce reason for the reversal<br><br>Response: We have kindly rephrased the sentence to make it clearer and we have explained the sentence. The main reason is that one-time spirometry may misclassify patients and hence, cannot be fully relied on to diagnose COPD.<br><br>if the The GLI equation is reported to significantly underestimate FEV1 and FVC by 9% in the Cameroonian population [32], why not use the Cameroun study or other reference equation in West Africa rather the GLI?<br>Response: We have re-analyzed the data using the reference equation from the Cameroonian study to determine the lower limit of normal and hence abnormal spirometric patterns.
**Additional Information:**	
**Question**	**Response**
**Financial Disclosure**<br><br>Enter a financial disclosure statement that describes the sources of funding for the work included in this submission. Review the submission guidelines for detailed	There was no funding for this study

requirements. View published research articles from *PLOS ONE* for specific examples.<br><br>This statement is required for submission and **will appear in the published article** if the submission is accepted. Please make sure it is accurate.<br><br>**Unfunded studies**<br>Enter: *The author(s) received no specific funding for this work.*<br><br>**Funded studies**<br>Enter a statement with the following details:<br>• Initials of the authors who received each award<br>• Grant numbers awarded to each author<br>• The full name of each funder<br>• URL of each funder website<br>• Did the sponsors or funders play any role in the study design, data collection and analysis, decision to publish, or preparation of the manuscript?<br>• **NO** - Include this sentence at the end of your statement: *The funders had no role in study design, data collection and analysis, decision to publish, or preparation of the manuscript.*<br>• **YES** - Specify the role(s) played.<br><br>* typeset	
**Competing Interests**<br><br>Use the instructions below to enter a competing interest statement for this submission. On behalf of all authors, disclose any competing interests that could be perceived to bias this work—acknowledging all financial support and any other relevant financial or non-financial competing interests.<br><br>This statement is required for submission and **will appear in the published article** if the submission is accepted. Please make sure it is accurate and that any funding sources listed in your Funding Information later in the submission form are also declared in your Financial Disclosure	The authors have declared that no competing interests exist.

statement.<br><br>View published research articles from *PLOS ONE* for specific examples.<br><br>**NO authors have competing interests**<br><br>Enter: *The authors have declared that no competing interests exist.*<br><br>**Authors with competing interests**<br><br>Enter competing interest details beginning with this statement:<br><br>*I have read the journal's policy and the authors of this manuscript have the following competing interests: [insert competing interests here]*<br><br>* typeset	
**Ethics Statement**<br><br>Enter an ethics statement for this submission. This statement is required if the study involved:<br><br>• Human participants<br>• Human specimens or tissue<br>• Vertebrate animals or cephalopods<br>• Vertebrate embryos or tissues<br>• Field research<br><br>Write "N/A" if the submission does not require an ethics statement.<br><br>General guidance is provided below. Consult the submission guidelines for detailed instructions. **Make sure that all information entered here is included in the Methods section of the manuscript.**	Ethical approval was obtained from the Korle Bu Teaching Hospital Institutional Review Board (Protocol ID number: KBTH-IRB/000131-2018) and all participants provided written voluntary informed consent before joining the study.

**Format for specific study types**<br><br>**Human Subject Research (involving human participants and/or tissue)**<br>• Give the name of the institutional review board or ethics committee that approved the study<br>• Include the approval number and/or a statement indicating approval of this research<br>• Indicate the form of consent obtained (written/oral) or the reason that consent was not obtained (e.g. the data were analyzed anonymously)<br><br>**Animal Research (involving vertebrate animals, embryos or tissues)**<br>• Provide the name of the Institutional Animal Care and Use Committee (IACUC) or other relevant ethics board that reviewed the study protocol, and indicate whether they approved this research or granted a formal waiver of ethical approval<br>• Include an approval number if one was obtained<br>• If the study involved *non-human primates*, add *additional details* about animal welfare and steps taken to ameliorate suffering<br>• If anesthesia, euthanasia, or any kind of animal sacrifice is part of the study, include briefly which substances and/or methods were applied<br><br>**Field Research**<br><br>Include the following details if this study involves the collection of plant, animal, or other materials from a natural setting:<br>• Field permit number<br>• Name of the institution or relevant body that granted permission	
**Data Availability**<br><br>Authors are required to make all data underlying the findings described fully available, without restriction, and from the time of publication. PLOS allows rare exceptions to address legal and ethical concerns. See the PLOS Data Policy and FAQ for detailed information.	No - some restrictions will apply

A Data Availability Statement describing where the data can be found is required at submission. Your answers to this question constitute the Data Availability Statement and **will be published in the article**, if accepted.<br><br>**Important:** Stating 'data available on request from the author' is not sufficient. If your data are only available upon request, select 'No' for the first question and explain your exceptional situation in the text box.<br><br>Do the authors confirm that all data underlying the findings described in their manuscript are fully available without restriction?	
**Describe where the data may be found in full sentences. If you are copying our sample text, replace any instances of XXX with the appropriate details.**<br><br>• If the data are **held or will be held in a public repository**, include URLs, accession numbers or DOIs. If this information will only be available after acceptance, indicate this by ticking the box below. For example: *All XXX files are available from the XXX database (accession number(s) XXX, XXX.).*<br>• If the data are all contained **within the manuscript and/or Supporting Information files**, enter the following: *All relevant data are within the manuscript and its Supporting Information files.*<br>• If neither of these applies but you are able to provide **details of access elsewhere**, with or without limitations, please do so. For example:<br><br>*Data cannot be shared publicly because of [XXX]. Data are available from the XXX Institutional Data Access / Ethics Committee (contact via XXX) for researchers who meet the criteria for access to confidential data.*<br><br>*The data underlying the results presented in the study are available from (include the name of the third party*	The dataset supporting the conclusion of this study is available for systematic review and meta-analysis upon request from the corresponding author.

and contact information or URL).<br>• This text is appropriate if the data are owned by a third party and authors do not have permission to share the data.<br><br>* typeset	
Additional data availability information:	Tick here if your circumstances are not covered by the questions above and you need the journal’s help to make your data available.

Powered by Editorial Manager® and ProduXion Manager® from Aries Systems Corporation

Department of Physiology
University of Ghana Medical School
March 9, 2023

The Editor
PLOS ONE journal

Dear Prof,

Submission of revised manuscript (Manuscript ID: PONE-D-22-21464)

Title of Paper: Abnormal spirometric patterns and respiratory symptoms in HIV patients with no recent pulmonary infection in a periurban hospital in Ghana

We sincerely thank the editor and reviewers for their constructive comments on our manuscript. We have accordingly revised the manuscript based on their comments and we believe that the quality has improved, and hopefully to the level that can be accepted for publication.

We have provided a point-by-point explanation of how we addressed the reviewers' comments below.

Disclosure*: The authors received no specific funding for this work. The authors also declare that they have no competing interest as well.*

We once again thank you for considering our manuscript.

Yours faithfully,

Dr Kwame Yeboah

Manuscript
Click here to access/download;Manuscript;manuscript 1.docx

Abnormal spirometric patterns and respiratory symptoms in HIV patients with no recent pulmonary infection in a periurban hospital in Ghana

Kwame Yeboah[1], Latif Musa[1,2], Kweku Bedu-Addo[2]

1. Department of Physiology, University of Ghana Medical School, Accra, Ghana
2. Department of Physiology, School of Medicine and Dentistry, KNUST, Kumasi, Ghana

Corresponding author: Dr Kwame Yeboah, Department of Physiology, University of Ghana Medical School, P O Box 4236, Accra, Ghana.

Email: kyeboah@ug.edu.gh/melvinky@gmail.com.

Short title: Abnormal spirometric patterns and respiratory symptoms in HIV patients

Abbreviations: SSA, sub-Saharan Africa; HIV, human immunodeficiency virus; PLWH, people living with HIV; cART, combination antiretroviral therapy; FEV1, forced expiratory volume in 1s; FVC, forced vital capacity; LLN, lower limit of normal; OSP, obstructive spirometric pattern; RSP, restrictive spirometric patterns; GLI, Global Lung Initiative.

Keywords: Abnormal spirometric patterns, Respiratory symptoms, HIV, cART, Ghana

Abstract

Background: Human immunodeficiency virus (HIV) infection is associated with chronic airway obstruction, even in those with viral suppression by combination antiretroviral treatment (cART). Spirometry is a supplementary test that aids in diagnosing pulmonary dysfunction in people living with HIV.

Aim: To compare the prevalence of spirometric abnormalities among cART-treated HIV patients and cART-naïve HIV patients with non-HIV controls with no recent history of pulmonary infection in a peri-urban hospital in Ghana.

Methods: In a case-control design, we recruited 158 cART-treated HIV patients, 150 cART-naïve HIV patients and 156 non-HIV controls for the study. Clinical, sociodemographic data and respiratory symptoms were collected using a structured questionnaire. Spirometric abnormalities were categorised as obstructive (OSP) or restrictive (RSP) spirometric patterns based on the Cameroonian reference equation.

Results: The prevalence of OSP was higher in the cART-treated and cART-naïve HIV patients compared to non-HIV controls (13.9% vs 10.7% vs 5.1% respectively, p=0.026), whereas that of RSP was similar among the study groups. Respiratory symptoms were common among cART-treated and cART-naïve HIV patients compared to non-HIV controls (48.1% vs 40% vs 19.2% respectively, p<0.001). The major determinants of OSP were female gender [OR (95% CI)=2.46 (1.09 – 5.13), p=0.031], former cigarette smoking [1.92 (1.04 – 3.89), p<0.001] exposure to medium-to-high levels of biomass [3.07 (1.16 – 8.73), p=0.019], presence of a respiratory symptom [1.89 (1.11 – 5.08), p=0.029] and unemployment [3.26 (1.19 – 8.95), p=0.042], and that of RSP were age, female gender [1.74 (1.05 – 4.29), p=0.041], former cigarette smoking [2.31 (1.27 – 6.77), p<0.001], medium-to-high biomass exposure [1.58 (1.06 – 5.37), p=0.043].

Conclusion: In HIV patients without any recent pulmonary infection in a peri-urban area of Ghana, there was a higher prevalence of OSP among cART-treated and cART naïve HIV patients compared to the non-HIV control. However, the prevalence of RSP was similar among various study groups.

Introduction

Sub-Saharan Africa (SSA) has the largest population of people living with human immunodeficiency virus (HIV) and this is associated with healthcare, socio-economic and developmental challenges [1]. It was reported in 2017 that 71% of the global total number of people living with HIV (PLWH) resides in SSA, with 75% of deaths and 65% of new HIV infections occurring in the region [2]. In Ghana, the prevalence of HIV infection is 1.7% and is concentrated mostly in urban areas [3]. With the introduction of effective combination antiretroviral therapy (cART), PLWH now lives longer due to decreased morbidity and mortality from opportunistic infections [4]. This has increased the susceptibility of PLWH to chronic diseases at greater rates than those observed in HIV-uninfected persons, making HIV infection a manageable chronic disease [5]. Chronic obstructive pulmonary disease (COPD) is one of the major causes of death and disability worldwide. HIV infection has been associated with an increased prevalence of COPD, with some studies suggesting that the prevalence of COPD is 21% higher in PLWH compared to the general population [6]. The high burden of COPD persists in PLWH even after adjustments for risk factors such as smoking, opportunistic infections and injection drug use [4, 7-10].

Spirometry is a simple, effective and reproducible method that can aid the diagnosis of COPD, and the results can be categorized into two major patterns of abnormalities. An obstructive spirometric pattern (OSP) is characterized by a disproportional reduction in the forced expiratory volume in 1 s (FEV1) relative to the forced vital capacity (FVC), leading to a reduction in the FEV1/FVC ratio to less than the lower limit of normal (LLN) of a similar population [11, 12]. A restrictive spirometric pattern (RSP) is defined by a normal FEV1/FVC (>LLN), but an FVC is less than 80% of the predicted value. Both obstructive and restrictive

spirometry patterns are associated with increased mortality in the general population [12, 13]. Despite the high prevalence of PLWH in SSA, few studies have reported the prevalence and risk factors for these abnormal spirometric patterns with recent reports from the FRESH AIR study in Uganda [5-7, 14-17]. Most of these studies attribute abnormal spirometry in HIV to opportunistic pulmonary infection. We compared the prevalence of spirometric abnormalities among HIV patients on cART treatment and cART-naïve HIV patients with non-HIV controls with no recent history of pulmonary infection in a peri-urban hospital in Ghana. We hypothesize that HIV infection would increase the prevalence of spirometric abnormalities compared to those with no HIV infection. Also, we identified various factors associated with spirometric abnormalities in PLWH in Ghana.

Methods

Study participants and design

This study was a case-control design, conducted from October 2019 through February 2020, with HIV patients as cases and the controls were non-HIV individuals who visited the facility for voluntary testing of their HIV status. HIV patients were categorized as those on cART management and cART-naïve HIV patients who have not been on cART treatment for more than 4 weeks. The study was conducted at Atua Government Hospital, a 150-bed primary healthcare facility, located in Agormanya, a peri-urban town in the Eastern region of Ghana. The Agormanya area has a higher prevalence of HIV infection (7%) compared to the national prevalence which stands at 1.7% in 2020 [3]. The hospital has about 1,500 HIV patients on its register. Participants younger than 20 years, those diagnosed with severe heart diseases or hospitalized for cardiopulmonary complications within the past year, those with a diagnosis of lung infection or disease from their clinical case notes or answering positively to the question:

“Have you been told by your health worker that you have lung disease?” were excluded from the study. In addition, we excluded pregnant women, any individual with mental illness, active or recent (within the past 24 months) tuberculosis or lung infection and those with other conditions contraindicated for spirometry. We invited 180 cART-treated HIV patients, 170 cART-naïve controls and 170 non-HIV controls to join the study. However, 22 cART-treated HIV patients, 20 cART-naïve HIV patients and 12 non-HIV controls failed to produce the appropriate manoeuvres required for accurate spirometry recordings, and hence, they were excluded from the analysis. Ethical approval was granted by the Korle Bu Teaching Hospital Institutional Review Board (Protocol ID number: KBTH-IRB/000131-2018) and all participants provided voluntary informed consent before joining the study.

Data collection

A structured questionnaire was used to obtain data on sociodemographic factors like age, gender, lifestyle factors (smoking, alcohol intake), medical history, current HIV medication, occupation, education, marital status and biomass exposure. Medium-high biomass exposure was defined as those who reported using firewood, sawdust, cow dung or corn cubs as a fuel source for work or cooking at home on most days [18]. Respiratory symptoms were assessed using a modified version of the American Thoracic Society respiratory questionnaire, which included the following questions: “Do you usually have coughs without cold for more than 2 months in a year?”, “Do you usually bring up phlegm from your chest when you don’t have a cold?”, “Do you have difficulty breathing when you walk fast, use a staircase or run?” and “Have you ever had an attack of wheezing or whistling in your chest that made you feel short of breath?” [19]. These questions were administered in the local dialect for participants with limited formal education.

Body weight and height were measured using a stadiometer in light clothing with footwear removed, with body mass index (BMI) calculated as weight/height^2 and categorized as: underweight (< 18.5 kg/m^2), normal weight (18.5–24.9 kg/m^2), overweight (25–29.9 kg/m^2), and obese (≥ 30 kg/m^2). Hypertension was defined as self-reported use of antihypertensive treatment and/or systolic blood pressure ≥ 140 mm Hg and/or diastolic blood pressure ≥ 90 mm Hg. We collected blood samples and measured the levels of CD4+ lymphocytes at the time of the study.

Spirometry was performed by a qualified technician using a Vitalograph Pneumotrac desktop spirometer model 6800 following the ATS/ERS 2019 standard guidelines [20]. Using the Cameroonian spirometric equation [21], we computed abnormal spirometric patterns based on fixed ratio and lower limits of normal (LLN). The normal spirometric pattern was defined by FEV1, FVC, and FEV1/FVC all > LLN. Obstructive spirometric pattern (OSP) was defined as FEV1/FVC<0.7 with a percentage predicted FVC>80% using the fixed ratio or FEV1/FVC <LLN with FVC > LLN using the LLN method. A restrictive spirometric pattern was defined as FEV1/FVC>0.7 with a percentage predicted FVC<80% by fixed ratio or FVC < LLN with FEV1/FVC > LLN [21].

Sample size

The sample size required was calculated using two studies conducted in rural Uganda that reported the prevalence of OSP in HIV patients to be 6.2% [6] and that of the non-HIV population to be 16.2% [22]. Therefore, a minimum of 153 participants would be required to achieve a power of 80% at a 95% confidence level with an estimated odds ratio of 2.93.

Data analysis

Data were analysed using IBM SPSS version 28 software. Continuous descriptive data were presented as means and standard deviations for normally distributed data and comparisons between groups were made using one-way ANOVA, while non-normally distributed data were presented as medians and interquartile ranges and comparisons were made using the Kruskal-Wallis' test. Categorical descriptive data were presented as frequencies and percentages and analysed using the chi-square test. Univariate and multivariable logistic regression models were used to analyse the change in odds of abnormal spirometry and respiratory symptoms with socio-demographic and clinical factors. $p < 0.05$ was considered statistically significant.

Results

General characteristics of study participants

The age range of participants was 20 – 70 years with a mean age of 38.4±13.7 years. There was no difference in mean age among various categories of participants. There was a high proportion of females in this study participants and participants who currently or formerly smoked were mostly HIV patients. HIV patients were likely to be unemployed or self-employed and had no formal education or attended school up to junior high level. HIV patients had greater biomass exposure from work or home and were more likely to be underweight. Measurements from spirometry indicate that cART-treated HIV patients had reduced FEV_1, FVC and FEF_{25-75} l/min compared to cART-naïve HIV patients and non-HIV participants. Compared to the non-HIV controls, the prevalence of OSP was high in cART-naïve and cART-treated HIV patients using the LLN. There was no difference in the prevalence of RSP using the LLN. When the fixed ratio was used to determine spirometric abnormalities, there was no difference in the prevalence of OSP and RSP among the study groups (Table 1). The average duration of HIV infection in cART-treated HIV patients was 7.6±4.6 years and the average duration of

cART treatment was 7.2±4.5 years. For the cART medication regimen, 94 (59.5%) patients were treated with TDF/3TC/NVP or EFV regimens, 52 (32.9%) patients were on AZT/3TC/NVP or EFV regimens and 12 (7.6%) patients were on LPV/r-based regimens.

Table 1. General characteristics of study participants

	All Participants (n=464)	Non-HIV participants (n=156)	cART-naïve HIV patients (n=150)	cART-treated HIV patients (n=158)	p
Age, years	38.4±13.7	36.7±14.4	38.2±11.6	39±11.4	0.109
Females, n (%)	312 (67.2)	106 (67.9)	84 (56)	122 (77.2)	0.02
Smoking, n (%)					0.029
Current	16 (3.4)	2 (1.3)	4 (2.7)	10 (6.3)	
Former	57 (15.9)	9 (5.8)	22 (14.6)	26 (16.5)	
Never	187 (80.6)	71 (92.9)	124 (82.7)	132 (83.5)	
Alcohol intake, n (%)	102 (22)	38 (24.4)	36 (24)	28 (17.7)	0.55
Married, n (%)	198 (42.7)	70 (44.9)	62 (41.3)	66 (41.8)	0.79
Education, n (%)					<0.001
None	50 (10.8)	4 (2.6)	14 (9.3)	32 (20.3)	
Up to JHS	208 (44.8)	24 (15.4)	80 (53.3)	104 (65.8)	
SHS to tertiary	68 (14.7)	24 (15.4)	24 (16)	20 (12.7)	
Tertiary	138 (29.7)	104 (66.7)	32 (21.3)	2 (1.3)	
Employment, n (%)					<0.001
Unemployed	56 (12.1)	22 (14.1)	20 (13.3)	14 (8.9)	
Self-employed	148 (31.9)	14 (9)	54 (36)	80 (50.6)	
Formal employment	260 (56)	120 (76.9)	76 (50.7)	64 (40.5)	
Biomass exposure, n (%)					<0.001
Low	342 (73.7)	132 (84.6)	96 (64)	114 (72.2)	
Medium-high	122 (26.3)	24 (15.4)	54 (36)	44 (27.8)	
Weight, kg	60±13.2	68±13.3	64.4±7.3	65.8±14	0.204
BMI, kg/m^2	24.8±5	25.4±4.7	23.7±4.4	25.3±5.7	0.061
BMI categories, n (%)					0.024
Underweight	34 (7.4)	6 (3.9)	16 (10.7)	12 (7.6)	
Normal	238 (51.5)	70 (45.5)	94 (62.7)	74 (46.8)	
Overweight	116 (25.1)	46 (29.9)	18 (12)	52 (32.9)	
Obese	74 (16)	32 (20.8)	22 (14.7)	20 (12.7)	
Current CD4 count, $cells/mm^2$	405 (273 – 562)		403 (253 – 583)	430 (327 – 534)	0.804
Spirometric measurements					
FEV1, l	2.2±0.6	2.4±0.6	2.3±0.7	$1.9{\pm}0.9^{*\#}$	<0.001
FVC, l	2.7±0.7	3±0.7	2.9±0.8	$2.3{\pm}0.5^{*\#}$	<0.001

	FEF_{25-75}, l/min	2.3±0.9	2.5±0.9	2.4±1	1.9±0.8[*#]	<0.001
	FEV1/FVC, %	80.3±6.9	81.3±5.7	80±7.4	79.4±7.3	0.129
Abnormal spirometry by LLN, n (%)						
	Obstructive	46 (9.9)	8 (5.1)	16 (10.7)	22 (13.9)	0.026
	Restrictive	24 (19.8)	26 (16.7)	20 (13.3)	36 (22.8)	0.087
Abnormal spirometry by a fixed ratio, n (%)						
	Obstructive	26 (5.6)	4 (2.6)	8 (5.3)	14 (8.9)	0.052
	Restrictive	92 (19.8)	28 (17.9)	26 (17.3)	38 (24.1)	0.258
Respiratory symptoms, n (%)		166 (35.8)	30 (19.2)	60 (40)	76 (48.1)	<0.001

cART, combination antiretroviral therapy; JHS, junior high school; SHS, senior high school; BMI, body mass index; FEV1, forced expiratory volume in 1 second; FVC, forced vital capacity.

*: p<0.05 compared to non-HIV
#: p< 0.05 compared to untreated HIV patients

Respiratory symptoms

Among the entire study participants, 35.8% reported the presence of at least one respiratory symptom, with a high proportion in cART-treated and cART-naïve HIV patients compared to non-HIV participants (48.1% vs 40% vs 19.2 respectively, p<0.001). Phlegm production and regular cough were commonly reported in 26.4% and 19% of the entire study participants respectively, with a high proportion in cART-treated and cART-naïve HIV patients compared to non-HIV participants (phlegm: 35.4% vs 26.7% vs 6.4%, p<0.001; regular coughs: 30.4% vs 20% vs 11.5%, p<0.001 respectively). Similar trends were observed in participants who reported having dyspnoea on exertion and frequent wheeze symptoms (Fig. 1). Increasing age, being unemployed, former cigarette smoking, exposure to a high-medium level of biomass, being HIV patients with or without cART treatment and having basic or no education was associated with increased odds of having respiratory symptoms in unadjusted regression models. In the adjusted regression models, exposure to medium-high levels of biomass, being a cART-treated HIV or cART-naïve HIV patient were associated with increased odds of having respiratory symptoms (Table 2).

Fig. 1 Prevalence of respiratory symptoms in study participants by the HIV status

Table 2. Factors associated with respiratory symptoms from logistic regression analyses

	Unadjusted model		Adjusted model	
	OR (95% CI)	p	OR (95% CI)	p
Age, yrs	1.02 (1.01 – 1.03)	0.007	1 (0.98 – 1.02)	0.734
Females, n	1.32 (0.87 – 1.99)	0.189	1.14 (0.69 – 1.88)	0.623
Unmarried	1.34 (0.91 – 1.97)	0.14	1.18 (0.76 – 1.86)	0.462
Alcohol intake	0.97 (0.62 – 1.54)	0.909	1 (0.59 – 1.68)	0.986
Smoking (reference: never smoked)				
Current	1.84 (0.68 – 4.98)	0.233	1.52 (0.5 – 4.64)	0.463
Former	2.75 (1.12 – 5.08)	<0.001	1.94 (1 – 4.65)	0.071
Medium/high biomass exposure	2.72 (1.12 – 4.57)	0.008	1.87 (1.07 – 3.49)	0.037
HIV status: reference= non-HIV				
cART-naïve	2.8 (1.67 – 4.69)	<0.001	2.03 (1.1 – 3.74)	0.023
cART-treated	3.89 (2.35 – 6.46)	<0.001	2.61 (1.32 – 5.17)	0.006
Employment				
Self-employed	1.4 (0.92 – 2.13)	0.118	0.83 (0.5 – 1.36)	0.451
Unemployed	1.88 (1.05 – 3.38)	0.035	1.78 (0.91 – 3.48)	0.091
Education: reference= tertiary				
None	3.32 (1.67 – 6.6)	<0.001	2.22 (0.89 – 5.51)	0.086
Basic/JHS	3.21 (1.97 – 5.22)	<0.001	1.79 (0.89 – 3.62)	0.102
SHS/Tech	0.93 (0.46 – 1.91)	0.85	0.62 (0.27 – 1.43)	0.263
BMI categories				
Underweight	1.85 (0.9 – 3.81)	0.095	1.58 (0.73 – 3.42)	0.25
Overweight	0.68 (0.42 – 1.1)	0.116	0.6 (0.35 – 1.03)	0.062
Obese	0.79 (0.45 – 1.37)	0.789	0.82 (0.44 – 1.51)	0.517

cART, combination antiretroviral therapy; JHS, junior high school; SHS, senior high school; BMI, body mass index.

Determinants of abnormal spirometric patterns

In logistic regression analyses, being female, unmarried, a former smoker, unemployed, having no formal education, underweight and having respiratory symptoms were associated with increased odds of having OSP in the unadjusted models. In the adjusted models, female gender, former cigarette smoking, medium-to-high biomass exposure, presence of respiratory symptoms, unemployed and being underweight were associated with increased odds of having OSP (Table 3). The determinants of OSP among various categories of participants based on HIV status are shown in the supplementary attachment (Table S1 – S3).

Table 3. Factors associated with an obstructive spirometric pattern from logistic regression analyses

	Unadjusted model		Adjusted model	
	OR (95% CI)	p	OR (95% CI)	p
Age	0.79 (0.57 – 1.07)	0.745	0.91 (0.73 – 1.41)	0.576
Females	3.18 (1.24 – 8.85)	0.005	2.46 (1.09 – 5.13)	0.031
Unmarried	2.81 (1.35 – 5.83)	0.006	2.31 (0.86 – 5.19)	0.125
Alcohol intake	0.53 (0.22 – 1.3)	0.166	0.69 (0.19 – 1.489)	0.631
Smoking (Reference: never smoked)				
Current	1.38 (0.3 – 6.28)	0.676	1.87 (0.36 – 9.75)	0.457
Former	2.67 (1.13 – 4.07)	<0.002	1.92 (1.04 – 3.89)	<0.001
Medium/high biomass exposure	1.49 (1.13 – 3.25)	0.004	3.07 (1.16 – 8.73)	0.019
Respiratory symptoms	2.35 (1.26 – 4.4)	0.008	1.89 (1.11 – 5.08)	0.029
HIV status (reference: non-HIV)				
cART-naïve	2.24 (0.93 – 5.4)	0.073	1.29 (0.69 – 3.68)	0.548
cART-treated	3.08 (1.33 – 7.15)	0.009	1.79 (1.03 – 4.18)	0.037
Employment				
Self-employed	0.51 (0.23 – 1.17)	0.112	0.94 (0.43 – 2.51)	0.481
Unemployed	2.46 (1.15 – 5.23)	0.02	3.26 (1.19 – 8.95)	0.042

Education (reference: tertiary)				
None	2.11 (1.08 – 6.71)	0.044	1.72 (0.79 – 6.04)	0.533
Basic/JHS	0.91 (0.43 – 1.9)	0.632	0.77 (0.32 – 1.26)	0.257
SHS/Tech	1.02 (0.36 – 2.84)		0.63 (0.41 – 1.73)	0.358
BMI categories				
Underweight	3.11 (1.27 – 10.21)	<0.001	3.01 (1.12 – 10.16)	0.026
Overweight	1.09 (0.54 – 2.28)	0.691	0.98 (0.59 – 2.71)	0.864
Obese	1.12 (0.53 – 2.78)	0.748	1.06 (0.49 – 4.02)	0.877

cART, combination antiretroviral therapy; JHS, junior high school; SHS, senior high school; BMI, body mass index.

Concerning RSP, female gender, former smoking, medium-to-high biomass exposure and being a cART-treated HIV patient were associated with increased odds in unadjusted logistic regression models. In multivariable logistic regression models, age, female gender, being unmarried, previous cigarette smoking and medium-to-high biomass exposure were associated with increased odds of restrictive lung pattern (Table 4). The determinants of RSP among various patient groups are shown in the supplementary attachment (Tables S4 – S6).

Table 4. Factors associated with a restrictive spirometric pattern from logistic regression analyses

	Unadjusted model		Adjusted model	
	OR (95% CI)	p	OR (95% CI)	p
Age	0.97 (0.93 – 1.01)	0.061	0.97 (0.65 – 1.29)	0.114
Females	1.67 (1.09 – 3.42)	0.013	1.74 (1.05 – 4.29)	0.041
Unmarried	0.75 (0.5 – 1.14)	0.18	1.31 (0.68 – 3.88)	0.741
Alcohol intake	1.43 (0.88 – 2.310	0.151	1.68 (0.96 – 2.95)	0.069
Smoking (reference: never)				
Current	0.4 (0.09 – 1.78)	0.229	0.42 (0.09 – 2.95)	0.283
Former	3.07 (1.64 – 8.36)	<0.001	2.31 (1.27 – 6.77)	<0.001
Medium/high biomass exposure	2.15 (1.13 – 5.37)	<0.001	1.58 (1.06 – 5.37)	0.043
HIV status: reference= non-HIV				

cART-naïve	0.89 (0.53 – 1.73)	0.888	1.67 (0.84 – 3.33)	0.144
cART-treated	1.75 (1.04 – 2.59)	0.016	4.89 (2.15 – 11.11)	<0.001
Employment				
Self-employed	0.72 (0.44 – 1.17)	0.182	0.73 (0.23 – 1.79)	0.407
Unemployed	1.83 (1 – 3.34)	0.05	2.06 (0.92 – 4.14)	0.061
Education: reference= tertiary				
None	1.89 (0.99 – 3.73)	0.067	2.12 (0.8 – 5.65)	0.131
Basic/JHS	0.99 (0.61 – 1.62)	0.979	0.97 (0.45 – 2.09)	0.94
SHS/Tech	0.49 (0.23 – 1.06)	0.069	0.43 (0.17 – 1.08)	0.071
BMI categories				
Underweight	0.84 (0.36 – 1.94)	0.678	1.31 (0.52 – 3.29)	0.562
Overweight	0.95 (0.57 – 1.57)	0.837	0.65 (0.34 – 1.16)	0.148
Obese	0.87 (0.48 – 1.6)	0.661	0.71 (0.36 – 1.14)	0.333

cART, combination antiretroviral therapy; JHS, junior high school; SHS, senior high school; BMI, body mass index.

Association between respiratory symptoms and abnormal spirometric patterns

In the entire study participants with OSP, 14 (31.8%) reported having regular cough, 16 (36.4%) reported regular phlegm production, and 12 (16.7%) reported frequent wheezing or dyspnoea on exertion. Among the participants with RSP, those reporting regular cough or frequent wheeze or dyspnoea on exertion were 24 (16.7) while 34 (23.6%) reported regular phlegm production. In logistic regression analyses, regular cough, frequent wheeze and dyspnoea on exertion were associated with increased odds of having OSP in crude and adjusted models (Fig. 2). Participants with RSP had increased odds of frequent wheeze and dyspnoea on exertion in unadjusted logistic regression analysis, but there was no association between respiratory symptoms and RSP in multivariable analyses (Fig. 3).

Fig. 2 Association between respiratory symptoms and obstructive spirometric pattern from unadjusted (left) and adjusted (right) logistic regression models.

Fig. 3 Association between respiratory symptoms and restrictive spirometric pattern from unadjusted (left) and adjusted (right) logistic regression models.

Discussion

Major findings

This is the first study to report the prevalence of abnormal spirometry in HIV patients in Ghana. The major findings of the study were 1) The prevalence of OSP was higher among the HIV patient groups compared to non-HIV controls, whereas the prevalence of RSP is similar among the study groups; 2) respiratory symptoms were common among HIV patients compared to non-HIV controls and were associated with abnormal spirometry; and 3) the major ~~determinants~~ of OSP were female gender, former cigarette smoking, exposure to medium-to-high levels of biomass, presence of a respiratory symptom, unemployment and underweight and that of RSP were age, female gender, being unmarried, medium-to-high biomass exposure and being self-employed or unemployed.

Abnormal spirometric patterns.

The high prevalence of OSP cART-treated HIV patients in our study is consistent with studies from other countries in sub-Saharan Africa. In the South African population, van Riel *et al* reported a higher prevalence of OSP in cART-treated HIV patients (34.2%) compared to cART-naïve HIV patients (11.2%). However, they used a cut-off of 20%-LLN instead of the globally accepted 5%-LLN, which was used to characterize OSP in our study, and this may explain the usually high prevalence of OSP reported in that particular study [17]. In contrast to the findings of our study, Pefura-Yone *et al* reported lower prevalences of OSP, which was similar among the HIV patients and non-HIV controls using the LLN cut-off. [16]. Other cross-sectional studies in HIV patients in SSA have reported OSP prevalence between 3.1 – 15.4% [6, 10, 14-17], and a metanalysis reported the prevalence of OSP in HIV patients from 30 observational studies involving 151, 686 participants around the globe to be between 5.6% and 10.6% [7], similar to what was found in this study. Few studies have compared the prevalence

of OSP in cART-treated HIV patients to that of cART-naïve HIV patients and non-HIV controls.

We found that the prevalence of RSP was similar among cART-naïve HIV patients and non-HIV controls, but more common in cART-treated HIV patients. Most spirometry studies conducted in HIV patients and other populations in SSA do not report the prevalence and associated factors of RSP, limiting our ability to compare the prevalence of RSP found in our study to other spirometry studies in SSA. The patterns of abnormal spirometry that were reported in our study differed from what was reported by Drummond *et al* reported in HIV patients in the United States. He found that, in a total of 37% abnormal spirometry, there was a high prevalence of OSP (27%) compared to RSP (10%) [9]. The prevalence of RSP observed in our non-HIV controls (16.7%) was similar to 7 – 14% reported in the general US population [9], but higher than 5.9% reported in the older population [23] and 4.7% reported in Peruvian adults [24], and far lower than to 36% reported by Schultz *et al* in Brazilians [25]. The variation in the prevalence of abnormal spirometric patterns may be due to different reference equations applied in various studies [25] and the fact that various insults may affect pulmonary health across various geographical locations.

The accuracy of the definition of RSP used in this and many other studies, FVC<80% predicted and FEV_1/FVC>LLN, in diagnosing RSP have been reported to be poor with low sensitivity but high negative predictive value to exclude patients without restrictive lung disease [26]. On the other hand, some studies reported that using FVC<80% predicted alone, without FEV_1/FVC>LLN, improves specificity and negative prediction of diagnosing restrictive lung disease [12]. These inconsistent definitions of RSP in literature and the failure of current guidelines to address this have resulted in the underappreciation of RSP values in spirometric studies. However, the utility and importance of RSP have been highlighted in some studies as a predictor of cardiovascular morbidity and mortality. For instance, the Cardiovascular Health

Study reported that RSP is a significant predictor of CVD mortality and non-CVD mortality in older Caucasians [26]. Similarly, the TESOAD study reported that, although consistent RSP is found in 50% of patients with one-time spirometry, it is associated with an increased risk of all-cause mortality, and specifically, an increased risk of dying from diabetes and heart diseases [13]. Therefore, there is a need for further investigation into the relevance of the high burden of RSP found in HIV patients and non-HIV controls in our study to characterize them properly to prevent future CVD events and deaths.

~~Determinants~~ *of abnormal spirometry*

The female gender was associated with an increased likelihood of having abnormal spirometry in our study. A systematic review has shown that females are more likely to report asthma and airway obstruction compared to men, but abnormal spirometry is similar in both genders [27, 28]. The association between female sex and abnormal spirometry found in our study may be due to the high representation of females in our study population (67.2%). We found an association between unemployment and OSP, consistent with what has been reported in multicentre studies like BOLD [29] and PLATINO [30] studies. This may be due to the debilitating nature of airway obstruction that may lead to cessation of work in affected patients [29, 30]. Unlike many studies that reported an association between cigarette smoking and airway obstruction, we did not find any association between current smoking and abnormal spirometry, possibly due to the low proportion of smokers in all our study participants (3.4%), but higher in HIV patient groups. Analysis of Demographic Health Surveys of 25 countries in Africa indicated that HIV patients with low socioeconomic status, that is the poors, less educated and manual workers, are more likely to smoke [31]. The prevalence of smoking in our study is similar to that of the general Ghanaian population (4.85%) as reported by the 2014 Ghana Demographic Health Survey [32]. On the other hand, previous smoking was associated with abnormal spirometric patterns and this is consistent with other studies [9, 25]. However,

in agreement with the findings of other studies, biomass exposure was associated with abnormal spirometry in our participants [6, 14]. Biomass fumes contain about 50 air pollutants, with significant exposure similar to regular cigarette smoking [33]. These pollutants can irritate the respiratory tract by causing inflammation and tissue injury such as the excessive release of matrix metalloproteinases and increased hyaluronidase activity [34] that damage the lungs. We did not find any association between the cART regimen used, CD4 cell count and abnormal spirometry, similar to what was reported in other HIV studies [6, 9, 14].

Respiratory symptoms

The prevalence of respiratory symptoms in our HIV population is similar to what has been reported in other sub-Saharan African populations. In urban Ugandan HIV patients, Ddungu *et al* reported the prevalence of any respiratory symptom to be 45%, with 30% reporting chronic cough, 21% reporting frequent phlegm production and wheezing and 26% reporting dyspnoea [15]. Similarly, in Cameroonian HIV patients, Pefura-Yone *et al* reported the prevalence of any respiratory symptom to be 47.5% while that of cough and dyspnoea were 25.5% and 36.4% respectively [16]. Contrary to the finding of our study, the prevalence of respiratory symptoms reported in Nigerian HIV patients was low (17%) [14] and HIV patients in South Africa had <4% of any respiratory symptoms [17]. Systematic review and meta-analysis of studies conducted in developed countries have similarly reported a high prevalence of respiratory symptoms irrespective of access to cART and high viremic control [8]. In this study, we found that HIV status and medium-high biomass exposure were associated with respiratory symptoms in the adjusted model. Respiratory symptoms are known to be common with increasing age due to prolonged exposure to environmental toxins and decreased physiological capacity [11]. Our population has a low smoking burden but high biomass exposure and environmental pollution common in the peri-urban area might be responsible for the high prevalence of respiratory symptoms [33]. With the popularity of spirometry for screening

airway obstruction, respiratory symptoms are being given less attention. However, the symptoms experienced by HIV patients indicates functional status and quality of life, even if they correlate poorly with spirometry [8].

Limitations of the study

This study was conducted in a single primary healthcare facility in peri-urban Ghana, and hence, the findings may differ from the urban and rural populations, as well as other healthcare facilities in a different stratum. We cannot infer causation from the findings of the study due to the cross-sectional design. It may be possible that some of our participants may have had abnormal spirometry, especially RSP, before HIV infection. The data on chronic respiratory symptoms may be affected by recall bias, which may lead to the misclassification of some study participants. We did not assess important parameters like physical activity level, cART adherence, previous AIDS events and viral suppression which are reported to have an impact on pulmonary health. In this study, airway obstruction was defined by single spirometry. It has been reported, in patients diagnosed with COPD using spirometry, 20% reversed from OSP to normal spirometry after one year of diagnosis, and this increased to 28% after two years. Among patients diagnosed with normal spirometric patterns, 11% progressed to OSP after one year and this increased to 17% after two years [35]. Therefore, using a single spirometric measurement in this study, we cautiously avoided categorising our study participants as having COPD to avoid this misclassification. In addition, we used the reference equations for the Cameroonian population to compute prediction values and LLN for spirometric values, which yield different outcomes compared to the GLI reference equations for African-Americans. The GLI equation is reported to significantly underestimate FEV1 and FVC by 9% in the Cameroonian population [36], implying significant errors in studies conducted in SSA that applied the GLI reference equation.

Conclusion

In conclusion, our study has shown that PLWH without any recent pulmonary infection in a peri-urban area of Ghana, the prevalence of OSP was high among cART-treated HIV compared to cART-naïve HIV patients and non-HIV control. The prevalence of RSP was similar among various study groups. This study has also shed light on the various predictors of spirometric abnormalities with the hope that future studies may investigate the possibility of using interventions targeted at the various risk factors to reduce pulmonary dysfunction in HIV patients.

Supporting information

Table S1. Determinants of obstructive lung pattern in cART-treated HIV patients
Table S2. Determinants of obstructive lung pattern in cART-naïve HIV patients
Table S3. Determinants of obstructive lung pattern in non-HIV controls
Table S4. Determinants of restrictive lung pattern in cART-treated HIV patients
Table S5. Determinants of restrictive lung pattern in cART-naïve HIV patients
Table S6. Determinants of restrictive lung pattern in non-HIV controls
Table S7. Respiratory symptoms and spirometric abnormalities among cART-treated HIV patients

Acknowledgements: Our sincere thanks go to the staff and patients of Atua Government Hospital who assisted in data collection, especially Mr Samuel Essel and Mrs Nneka Essel.

Funding: There was no funding for this study.

Availability of data and materials: The dataset supporting the conclusion of this study is available for systematic review and meta-analysis upon request from the corresponding author.

Contributions:

KY contributed to the conception, design, data analysis, and drafting of the manuscript and bears the primary responsibility for the content of the manuscript.

LM collected the data and revised the manuscript.

KBA was involved in the supervision and revision of the manuscript.

All the authors read and approved the content of the manuscript.

Ethics approval and consent to participate

The study was conducted in conformity with the Helsinki Declaration on Human Experimentation, 1964 with subsequent revisions, latest Seoul, October 2008. Ethical approval for the study was granted by the Korle Bu Teaching Hospital Institutional Review Board (Protocol ID number: KBTH-IRB/000131-2018). All the participants provided voluntary written consent before being recruited into the study.

Consent for publication: Not applicable

Competing interests: The authors declare that they have no competing interests.

Reference

1. James SL, Abate D, Abate KH, Abay SM, Abbafati C, Abbasi N, Abbastabar H, Abd-Allah F, Abdela J, Abdelalim A: **Global, regional, and national incidence, prevalence, and years lived with disability for 354 diseases and injuries for 195 countries and territories, 1990–2017: a systematic analysis for the Global Burden of Disease Study 2017**. *The Lancet* 2018, **392**(10159):1789-1858.
2. Roth GA, Abate D, Abate KH, Abay SM, Abbafati C, Abbasi N, Abbastabar H, Abd-Allah F, Abdela J, Abdelalim A: **Global, regional, and national age-sex-specific mortality for 282 causes of death in 195 countries and territories, 1980–2017: a systematic analysis for the Global Burden of Disease Study 2017**. *The Lancet* 2018, **392**(10159):1736-1788.
3. Ghana AIDS Commission: **National and Sub-National HIV and AIDS Estimates and Projections 2020 Report**. In., vol. 2020. Accra: Ghana AIDS Commision; 2021.
4. Teeraananchai S, Kerr S, Amin J, Ruxrungtham K, Law M: **Life expectancy of HIV-positive people after starting combination antiretroviral therapy: a meta-analysis**. *HIV medicine* 2017, **18**(4):256-266.
5. Yang H-Y, Beymer MR, Suen S-c: **Chronic Disease Onset Among People Living with HIV and AIDS in a Large Private Insurance Claims Dataset**. *Scientific Reports* 2019, **9**(1):18514.
6. Kayongo A, Wosu AC, Naz T, Nassali F, Kalyesubula R, Kirenga B, Wise RA, Siddharthan T, Checkley W: **Chronic Obstructive Pulmonary Disease Prevalence and Associated Factors in a Setting of Well-Controlled HIV, A Cross-Sectional Study**. *COPD: Journal of Chronic Obstructive Pulmonary Disease* 2020, **17**(3):297-305.
7. Bigna JJ, Kenne AM, Asangbeh SL, Sibetcheu AT: **Prevalence of chronic obstructive pulmonary disease in the global population with HIV: a systematic review and meta-analysis**. *The Lancet Global Health* 2018, **6**(2):e193-e202.
8. Brown J, Roy A, Harris R, Filson S, Johnson M, Abubakar I, Lipman M: **Respiratory symptoms in people living with HIV and the effect of antiretroviral therapy: a systematic review and meta-analysis**. *Thorax* 2017, **72**(4):355-366.
9. Drummond MB, Huang L, Diaz PT, Kirk GD, Kleerup EC, Morris A, Rom W, Weiden MD, Zhao E, Thompson B *et al*: **Factors associated with abnormal spirometry among HIV-infected individuals**. *Aids* 2015, **29**(13):1691-1700.
10. Gupte AN, Wong ML, Msandiwa R, Barnes GL, Golub J, Chaisson RE, Hoffmann CJ, Martinson NA: **Factors associated with pulmonary impairment in HIV-infected South African adults**. *PLOS ONE* 2017, **12**(9):e0184530.

11. Vaz Fragoso CA, Gill TM: **Respiratory Impairment and the Aging Lung: A Novel Paradigm for Assessing Pulmonary Function**. *The Journals of Gerontology: Series A* 2011, **67A**(3):264-275.
12. Venkateshiah SB, Ioachimescu OC, McCarthy K, Stoller JK: **The Utility of Spirometry in Diagnosing Pulmonary Restriction**. *Lung* 2008, **186**(1):19-25.
13. Guerra S, Sherrill DL, Venker C, Halonen M, Martinez FD: **Chronic Restrictive Lung Pattern Is Associated with Increased Mortality Risk**. In: *American Thoracic Society 2009 International Conference.* vol. C34. COPD PHENOTYPES. San Diego Convention centre: American Journal of Respiratory and Critical Care Medicine; 2009: A4325.
14. Akanbi MO, Taiwo BO, Achenbach CJ, Ozoh OB, Obaseki DO, Sule H, Agbaji OO, Ukoli CO: **HIV Associated Chronic Obstructive Pulmonary Disease in Nigeria**. *J AIDS Clin Res* 2015, **6**(5).
15. Ddungu A, Semitala FC, Castelnuovo B, Sekaggya-Wiltshire C, Worodria W, Kirenga BJ: **Chronic obstructive pulmonary disease prevalence and associated factors in an urban HIV clinic in a low income country**. *PLOS ONE* 2021, **16**(8):e0256121.
16. Pefura-Yone EW, Fodjeu G, kengne AP, Roche N, Kuaban C: **Prevalence and determinants of chronic obstructive pulmonary disease in HIV infected patients in an African country with low level of tobacco smoking**. *Respiratory Medicine* 2015, **109**(2):247-254.
17. van Riel SE, Klipstein-Grobusch K, Barth RE, Grobbee DE, Feldman C, Shaddock E, Stacey SL, Venter WDF, Vos AG: **Predictors of impaired pulmonary function in people living with HIV in an urban African setting**. *South Afr J HIV Med* 2021, **22**(1):1252.
18. Sidhu MK, Ravindra K, Mor S, John S: **Household air pollution from various types of rural kitchens and its exposure assessment**. *Science of The Total Environment* 2017, **586**:419-429.
19. Ferris BG: **Epidemiology Standardization Project (American Thoracic Society)**. *Am Rev Respir Dis* 1978, **118**(6 Pt 2):1-120.
20. Graham BL, Steenbruggen I, Miller MR, Barjaktarevic IZ, Cooper BG, Hall GL, Hallstrand TS, Kaminsky DA, McCarthy K, McCormack MC *et al*: **Standardization of Spirometry 2019 Update. An Official American Thoracic Society and European Respiratory Society Technical Statement**. *American Journal of Respiratory and Critical Care Medicine* 2019, **200**(8):e70-e88.
21. Pefura-Yone EW, Balkissou AD, Poka-Mayap V, Djenabou A, Massongo M, Ofimboudem NA, Mayoh-Nguemfo CF, Tsala AG, Hadjara H, Amougou F: **Spirometric reference equations for Cameroonians aged 4 to 89 years derived using lambda, mu, sigma (LMS) method**. *BMC Pulmonary Medicine* 2021, **21**(1):344.
22. van Gemert F, Kirenga B, Chavannes N, Kamya M, Luzige S, Musinguzi P, Turyagaruka J, Jones R, Tsiligianni I, Williams S *et al*: **Prevalence of chronic obstructive pulmonary disease and associated risk factors in Uganda (FRESH AIR Uganda): a prospective cross-sectional observational study**. *Lancet Glob Health* 2015, **3**(1):e44-51.
23. Vaz Fragoso CA, Van Ness PH, Murphy TE, McAvay GJ: **Spirometric impairments, cardiovascular outcomes, and noncardiovascular death in older persons**. *Respiratory Medicine* 2018, **137**:40-47.
24. Siddharthan T, Grigsby M, Miele CH, Bernabe-Ortiz A, Miranda JJ, Gilman RH, Wise RA, Porter JC, Hurst JR, Checkley W: **Prevalence and risk factors of restrictive spirometry in a cohort of Peruvian adults**. *Int J Tuberc Lung Dis* 2017, **21**(9):1062-1068.
25. Schultz K, D'Aquino LC, Soares MR, Gimenez A, Pereira CAdC: **Lung volumes and airway resistance in patients with a possible restrictive pattern on spirometry**. *Jornal Brasileiro de Pneumologia* 2016, **42**:341-347.
26. Hyatt RE, Cowl CT, Bjoraker JA, Scanlon PD: **Conditions Associated With an Abnormal Nonspecific Pattern of Pulmonary Function Tests**. *CHEST* 2009, **135**(2):419-424.
27. Aryal S, Diaz-Guzman E, Mannino DM: **COPD and gender differences: an update**. *Translational Research* 2013, **162**(4):208-218.
28. Camp PG, Goring SM: **Gender and the Diagnosis, Management, and Surveillance of Chronic Obstructive Pulmonary Disease**. *Proceedings of the American Thoracic Society* 2007, **4**(8):686-691.
29. Grønseth R, Erdal M, Tan WC, Obaseki DO, Amaral Andre FS, Gislason T, Juvekar SK, Koul PA, Studnicka M, Salvi S *et al*: **Unemployment in chronic airflow obstruction around the world: results from the BOLD study**. *European Respiratory Journal* 2017, **50**(3):1700499.
30. Montes de Oca M, Halbert RJ, Talamo C, Perez-Padilla R, Lopez MV, Muiño A, Jardim JRB, Valdivia G, Pertuzé J, Moreno D *et al*: **Paid employment in subjects with and without chronic obstructive pulmonary disease in five Latin American cities: the PLATINO study**. *The International Journal of Tuberculosis and Lung Disease* 2011, **15**(9):1259-1264.
31. Murphy JD, Liu B, Parascandola M: **Smoking and HIV in Sub-Saharan Africa: A 25-Country Analysis of the Demographic Health Surveys**. *Nicotine & Tobacco Research* 2018, **21**(8):1093-1102.
32. Nketiah-Amponsah E, Afful-Mensah G, Ampaw S: **Determinants of cigarette smoking and smoking intensity among adult males in Ghana**. *BMC Public Health* 2018, **18**(1):941.
33. Torres-Duque C, Maldonado D, Pérez-Padilla R, Ezzati M, Viegi G: **Biomass Fuels and Respiratory Diseases**. *Proceedings of the American Thoracic Society* 2008, **5**(5):577-590.
34. Silva R, Oyarzún M, Olloquequi J: **Pathogenic Mechanisms in Chronic Obstructive Pulmonary Disease Due to Biomass Smoke Exposure**. *Archivos de Bronconeumología (English Edition)* 2015, **51**(6):285-292.

35. Schermer TR, Robberts B, Crockett AJ, Thoonen BP, Lucas A, Grootens J, Smeele IJ, Thamrin C, Reddel HK: **Should the diagnosis of COPD be based on a single spirometry test?** *npj Primary Care Respiratory Medicine* 2016, **26**(1):16059.
36. Pefura-Yone EW, Balkissou AD, Poka-Mayap V, Djenabou A, Massongo M, Ofimboudem NA, Mayoh-Nguemfo CF, Tsala AG, Hadjara H, Amougou F: **Spirometric reference equations for Cameroonians aged 4 to 89 years derived using lambda, mu, sigma (LMS) method**. *BMC Pulm Med* 2021, **21**(1):344.

 Click here to access/download;Figure;Figure 1 .docx

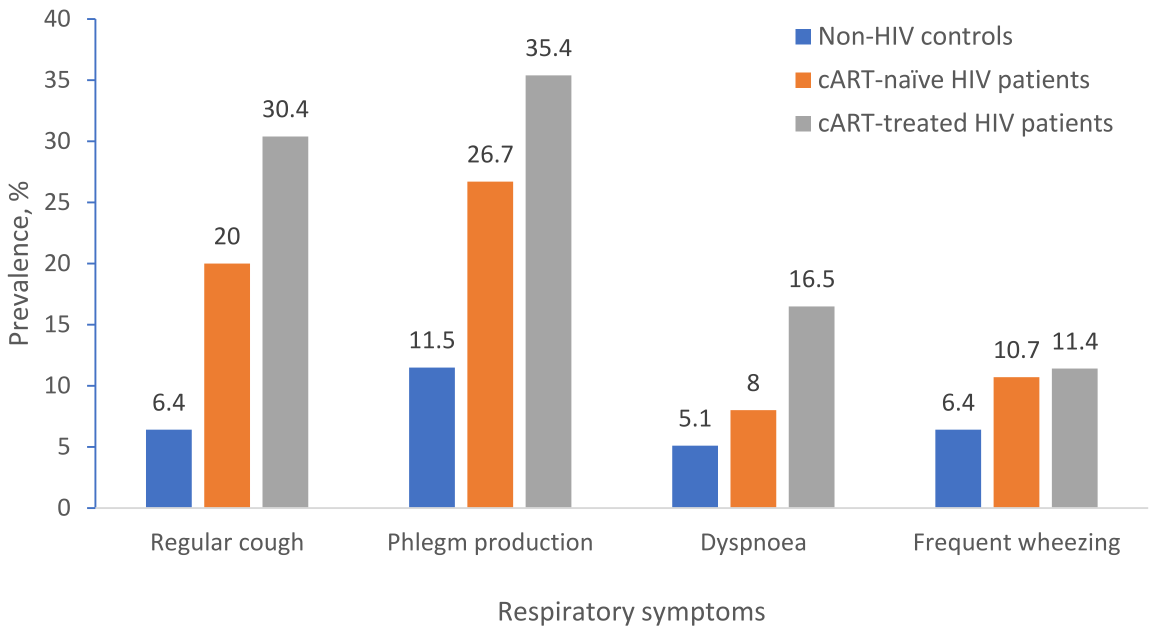

Fig 1. Prevalence of respiratory symptoms in study participants by the HIV status

HIV status was associated with regular cough (χ^2=29.5, p<0.001), phlegm production (χ^2=27.4, p<0.001) and dyspnoea (χ^2=12.2, p=0.002), but no association with frequent wheeze (χ^2=4.8, p=0.091).

Click here to access/download;Figure;Figure 2 .docx

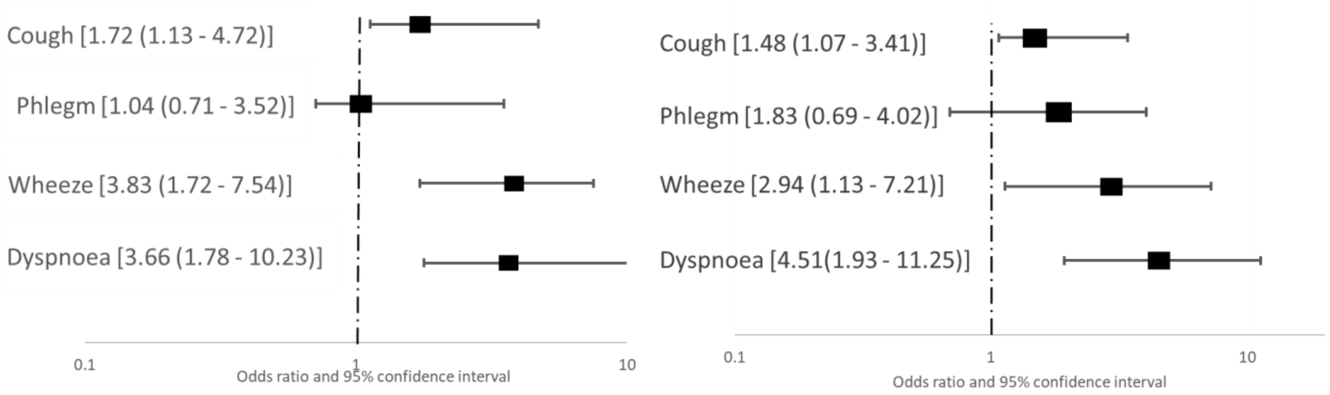

Fig. 2 Association between respiratory symptoms and obstructive spirometric pattern from unadjusted (left) and adjusted (right) logistic regression models.

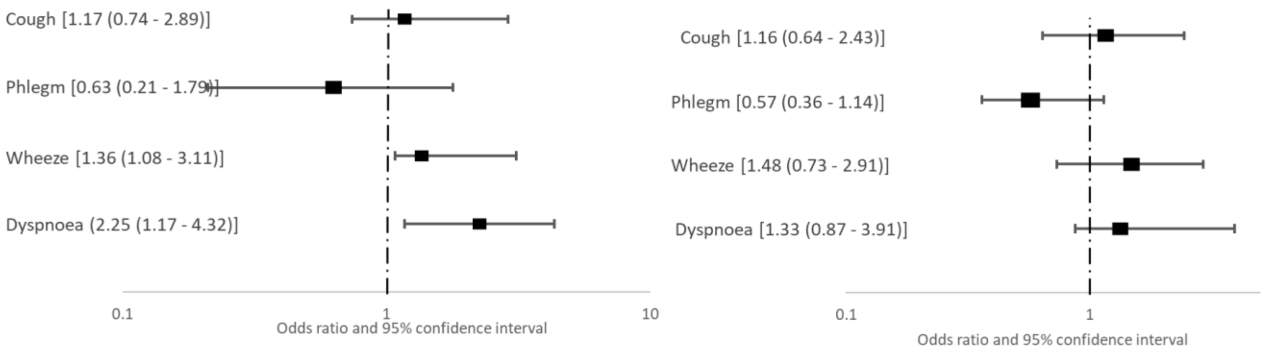

Fig. 3 Association between respiratory symptoms and restrictive spirometric pattern from unadjusted (left) and adjusted (right) logistic regression models.

Click here to access/download
Supporting Information
Supplementary Digital Content.docx

Click here to access/download
Other
jpg2pdf.pdf

Abnormal spirometric patterns and respiratory symptoms in HIV patients with no recent pulmonary infection in a periurban hospital in Ghana

Formatted: Left

Kwame Yeboah[1], Latif Musa[1,2], Kweku Bedu-Addo[2]

1. Department of Physiology, University of Ghana Medical School, Accra, Ghana
2. Department of Physiology, School of Medicine and Dentistry, KNUST, Kumasi, Ghana

Corresponding author: Dr Kwame Yeboah, Department of Physiology, University of Ghana Medical School, P O Box 4236, Accra, Ghana.

Email: kyeboah@ug.edu.gh/melvinky@gmail.com.

Short title: Abnormal spirometric patterns and respiratory symptoms in HIV patients

Abbreviations: SSA, sub-Saharan Africa; HIV, human immunodeficiency virus; PLWH, people living with HIV; ~~HAART, highly active~~cART, combination antiretroviral therapy; FEV1, forced expiratory volume in 1s; FVC, forced vital capacity; LLN, lower limit of normal; OSP, obstructive spirometric pattern; RSP, restrictive spirometric patterns; GLI, Global Lung Initiative.

Keywords: Abnormal spirometric patterns, Respiratory symptoms, HIV, ~~HAART~~cART, Ghana

Abstract

Background: Human immunodeficiency virus (HIV) infection is associated with chronic ~~pulmonary diseases~~airway obstruction, even in those with viral suppression by ~~highly active~~combination antiretroviral treatment (~~HAART~~cART). Spirometry is ~~an accurate method~~

~~of~~a supplementary test that aids in diagnosing pulmonary dysfunction in people living with HIV~~ (PLWH)~~..

Aim: To compare the prevalence of spirometric abnormalities among ~~HAART~~cART-treated HIV patients and ~~HAART~~ cART-naïve HIV patients with non-HIV controls with no recent history of pulmonary infection in a peri-urban hospital in Ghana.

Methods: In a case-control design, we recruited 158 ~~HAART~~cART-treated HIV patients, 150 ~~HAART~~cART-naïve HIV patients and 156 non-HIV controls for the study. Clinical, sociodemographic data and respiratory symptoms were collected using a structured questionnaire. ~~Spirometry was performed in all participants and~~Spirometric abnormalities were categorised as obstructive (OSP) or restrictive (RSP) spirometric patterns based on the ~~GLI definition~~Cameroonian reference equation.

Results: The prevalence of OSP was ~~similar among~~higher in the ~~HAART~~ cART-treated~~,~~ ~~HAART~~ and cART-naïve HIV patients ~~and~~compared to non-HIV controls (~~10.1~~13.9% vs ~~9.3~~10.7% vs ~~9~~5.1% respectively, p=0.~~994~~026), whereas that of RSP was ~~higher in HAART-treated HIV patients compared to HAART-naïve HIV patients and non-HIV controls (51.9% vs 32.1% vs 32% respectively, p=0.013).~~similar among the study groups. Respiratory symptoms were common among ~~HAART~~cART-treated and ~~HAART~~cART-naïve HIV patients compared to non-HIV controls (48.1% vs 40% vs 19.2% respectively, p<0.001). The major determinants of OSP were female gender~~,~~ [OR (95% CI)=2.46 (1.09 – 5.13), p=0.031], former cigarette smoking [1.92 (1.04 – 3.89), p<0.001] exposure to medium-to-high levels of biomass~~,~~ [3.07 (1.16 – 8.73), p=0.019], presence of a respiratory symptom~~,~~ [1.89 (1.11 – 5.08), p=0.029] and unemployment ~~and underweight,~~[3.26 (1.19 – 8.95), p=0.042], and that of RSP were age, female gender~~, being unmarried,~~ [1.74 (1.05 – 4.29), p=0.041], former cigarette smoking [2.31

(1.27 – 6.77), p<0.001], medium-to-high biomass exposure ~~and being self-employed or unemployed.~~[1.58 (1.06 – 5.37), p=0.043].

Conclusion: In HIV patients without any recent pulmonary infection in a peri-urban area of Ghana, there was ~~no difference in the~~ a higher prevalence of OSP among ~~HAART~~cART-treated and ~~HAART~~cART naïve HIV patients compared to the non-HIV control. However, the prevalence of RSP was ~~higher in HAART-treated HIV patients compared to the other~~similar among various study groups.

Introduction

Sub-Saharan Africa (SSA) has the largest population of people living with human immunodeficiency virus (HIV) and this is associated with healthcare, socio-economic and developmental challenges [1]. It was reported in 2017 that 71% of the global total number of people living with HIV (PLWH) resides in SSA, with 75% of deaths and 65% of new HIV infections occurring in the region [2]. In Ghana, the prevalence of HIV infection is 1.7% and is concentrated mostly in urban areas [3]. With the introduction of effective ~~highly active~~combination antiretroviral therapy (~~HAART~~cART), PLWH now ~~live~~lives longer due to decreased morbidity and mortality from opportunistic infections [4]. This has increased the susceptibility of PLWH to chronic diseases at greater rates than those observed in HIV-uninfected persons, making HIV infection a manageable chronic disease [5]. ~~Several studies have reported a high burden of chronic lung diseases in PLWH,~~Chronic obstructive pulmonary disease (COPD) is one of the major causes of death and disability worldwide. HIV infection has been associated with an increased prevalence of COPD, with some studies suggesting that

the prevalence of COPD is 21% higher in PLWH compared to the general population [6]. The high burden of COPD persists in PLWH even after adjustments for risk factors such as smoking, opportunistic infections and injection drug use [4, 6-97-10].

Spirometry is a simple, effective and reproducible method for diagnosing chronic lung diseasesthat can aid the diagnosis of COPD, and the results can be categorized into two major patterns of abnormalities. An obstructive spirometric pattern (OSP) is characterized by a disproportional reduction in the forced expiratory volume in 1 s (FEV1) relative to the forced vital capacity (FVC), leading to a reduction in the FEV1/FVC ratio to less than the lower limit of normal (LLN) of a similar population [10, 11, 12]. A restrictiveRestrictive spirometric pattern (RSP) is defined by a normal FEV1/FVC (>LLN), but an FVC is less than 80% of the predicted value. Both obstructive and restrictive spirometry patterns are associated with increased mortality in the general population [11, 12, 13]. Despite the high prevalence of PLWH in SSA, few studies have reported the prevalence and risk factors for these abnormal spirometric patterns with recent reports from the FRESH AIR study in Uganda [5, 6, 13-7, 14-17]. Most of these studies attribute abnormal spirometry in HIV to opportunistic pulmonary infection. We compared the prevalence of spirometric abnormalities among HIV patients on HAARTcART treatment and HAARTcART-naïve HIV patients with non-HIV controls with no recent history of pulmonary infection in a peri-urban hospital in Ghana. We hypothesize that HIV infection would increase the prevalence of spirometric abnormalities compared to those with no HIV infection. Also, we identified various factors associated with spirometric abnormalities in PLWH in Ghana.

Methods

Study participants and design

This study was a case-control design, conducted from October 2019 through February 2020, with HIV patients as cases and the controls were non-HIV individuals who visited the facility for voluntary testing of their HIV status. HIV patients were categorized as those on ~~HAART~~cART management and ~~HAART~~cART-naïve HIV patients who have not been on ~~HAART~~cART treatment for more than 4 weeks. The study was conducted at Atua Government Hospital, a 150-bed primary healthcare facility, located in Agormanya, a peri-urban town in the Eastern region of Ghana. The Agormanya area has a higher prevalence of HIV infection (~~11.6~~7%) compared to the national prevalence which stands at 1.7% in 2020 [3]. The hospital has about 1,500 HIV patients on its register. ~~Participants with conditions contraindicated for spirometry,~~Participants younger than 20 years, those diagnosed with severe heart diseases or hospitalized for cardiopulmonary complications within the past year, those with a diagnosis of lung infection or disease from their clinical case notes or answering positively to the question: "Have you been told by your health worker that you have lung disease ~~in the past years?" were excluded from the study.~~?" were excluded from the study. In addition, we excluded pregnant women, any individual with mental illness, active or recent (within the past 24 months) tuberculosis or lung infection and those with other conditions contraindicated for spirometry. We invited 180 cART-treated HIV patients, 170 cART-naïve controls and 170 non-HIV controls to join the study. However, 22 cART-treated HIV patients, 20 cART-naïve HIV patients and 12 non-HIV controls failed to produce the appropriate manoeuvres required for accurate spirometry recordings, and hence, they were excluded from the analysis. Ethical approval was granted by the Korle Bu Teaching Hospital Institutional Review Board (Protocol ID number: KBTH-IRB/000131-2018) and all participants provided voluntary informed consent before joining the study.

Formatted: Font: Georgia, 11 pt, Font color: Custom Color(RGB(46,46,46))

Data collection

A structured questionnaire was used to obtain data on sociodemographic factors like age, gender, lifestyle factors (smoking, alcohol intake), medical history (hypertension, diabetes, cardiovascular disease), current medication (pulmonary medication, antihypertensive agents, anti-diabetic drugs),, current HIV medication, occupation, education, marital status and biomass exposure. Medium-high biomass exposure was defined as those who reported using firewood, sawdust, cow dung or corn cubs as a fuel source for work or cooking at home on most days for more than 6 months. dung or corn cubs as a fuel source for work or cooking at home on most days [18]. Respiratory symptoms were assessed using a modified version of the American Thoracic Society respiratory questionnaire, which included the following questions: “Do you usually have coughs without cold for more than 2 months in a year?”, “Do you usually bring up phlegm from your chest when you don’t have a cold?”, “Do you have difficulty breathing when you walk fast, use a staircase or run?” and “Have you ever had an attack of wheezing or whistling in your chest that made you feel short of breath?” [18]. [19]. These questions were administered in the local dialect for participants with limited formal education.

Body weight and height were measured using a stadiometer in light clothing with footwear removed, with body mass index (BMI) calculated as weight/height^2 and categorized as: underweight (< 18.5 kg/m^2), normal weight (18.5–24.9 kg/m^2), overweight (25–29.9 kg/m^2), and obese ($\geq$ 30 kg/m^2). Hypertension was defined as self-reported use of antihypertensive treatment and/or systolic blood pressure $\geq$ 140 mm Hg and/or diastolic blood pressure $\geq$ 90 mm Hg. We collected blood samples and measured the levels of CD4+ lymphocytes at the time of the study.

Spirometry was performed by a qualified technician using a Vitalograph Pneumotrac desktop spirometer model 6800 following the ATS/ERS 2019 standard guidelines [19]. Using the Global Lung Initiative (GLI) 2012 reference equations for African Americans, normal lung function pattern was defined by FEV1, FVC, and FEV1/FVC all > LLN. Obstructive lung

function was determined by the ratio of Forced expiratory volume in the first second (FEV1) to Forced Vital Capacity (FVC) that was below the LLN with Forced Vital Capacity above LLN (FEV1/FVC <LLN with FVC > LLN), while restrictive lung function abnormalities were suggested by values less than the LLN for the FVC (FVC < LLN with FEV1/FVC > LLN) [20][20]. Using the Cameroonian spirometric equation [21], we computed abnormal spirometric patterns based on fixed ratio and lower limits of normal (LLN). The normal spirometric pattern was defined by FEV1, FVC, and FEV1/FVC all > LLN. Obstructive spirometric pattern (OSP) was defined as FEV1/FVC<0.7 with a percentage predicted FVC>80% using the fixed ratio or FEV1/FVC <LLN with FVC > LLN using the LLN method. A restrictive spirometric pattern was defined as FEV1/FVC>0.7 with a percentage predicted FVC<80% by fixed ratio or FVC < LLN with FEV1/FVC > LLN [21].

Sample size

The sample size required for the studies was calculated based on the prevalence of COPD reported in the rural Ugandan population to be 16.2% [21]. With the assumption that the prevalence ratio in the HIV population would be 2.2, we will need at least 147 participants in each group to achieve a power of 80% within a 95% significance level.The sample size required was calculated using two studies conducted in rural Uganda that reported the prevalence of OSP in HIV patients to be 6.2% [6] and that of the non-HIV population to be 16.2% [22]. Therefore, a minimum of 153 participants would be required to achieve a power of 80% at a 95% confidence level with an estimated odds ratio of 2.93.

Data analysis

Data were analysed using IBM SPSS version 28 software. Continuous descriptive data were presented as means and standard deviations for normally distributed data and comparisons between groups were made using one-way ANOVA, while non-normally distributed data were

presented as medians and interquartile ranges and comparisons were made using the Kruskal-Wallis' test. Categorical descriptive data were presented as frequencies and percentages and analysed using the chi-square test. Univariate and multivariable logistic regression models were used to analyse the change in odds of abnormal spirometry and respiratory symptoms with socio-demographic and clinical factors. $p< 0.05$ was considered statistically significant.

Results

General characteristics of study participants

The age range of participants was 20 – 70 years with a mean age of 38.4±13.7 years. There was no difference in mean age among various categories of participants. There was a high proportion of females in this study participants and participants who currently or formerly smoked were mostly HIV patients. HIV patients were likely to be unemployed or self-employed and had no formal education or attended school up to junior high level. HIV patients had greater biomass exposure from work or home and were more likely to be underweight. Measurements from spirometry indicate that ~~HAART~~cART-treated HIV patients had reduced FEV_1, FVC and FEF_{25-75} l/min compared to ~~HAART~~cART-naïve HIV patients and non-HIV participants. ~~HAART~~Compared to the non-HIV controls, the prevalence of OSP was high in cART-naïve and cART-treated HIV patients ~~had a high~~using the LLN. There was no difference in the prevalence of RSP ~~compared to HAART-naïve HIV patients and non-HIV controls, but~~using the LLN. When the fixed ratio was used to determine spirometric abnormalities, there was no difference in the prevalence of OSP ~~or undefined pulmonary patterns~~and RSP among ~~various~~the study groups ~~of participants~~ (Table 1). The average duration of HIV infection in cART-treated HIV patients was 7.6±4.6 years and the average duration of cART treatment was 7.2±4.5 years. For the cART medication regimen, 94 (59.5%) patients were treated with TDF/3TC/NVP or EFV

regimens, 52 (32.9%) patients were on AZT/3TC/NVP or EFV regimens and 12 (7.6%) patients were on LPV/r-based regimens.

Formatted: Font: 11 pt

Table 1. General characteristics of study participants

Formatted Table

	All Participants (n=464)	Non-HIV participants (n=156)	~~HAART~~cART-naïve HIV patients (n=150)	~~HAART~~cART-treated HIV patients (n=158)	p
Age, years	38.4±13.7	36.7±14.4	38.2±11.6	39±11.4	0.109
Females, n (%)	312 (67.2)	106 (67.9)	84 (56)	122 (77.2)	0.02
Smoking-, n (%)					0.029
Current	16 (3.4)	2 (1.3)	4 (2.7)	10 (6.3)	
Former	57 (15.9)	9 (5.8)	22 (14.6)	26 (16.5)	
Never	187 (80.6)	71 (92.9)	124 (82.7)	132 (83.5)	
Alcohol intake, n (%)	102 (22)	38 (24.4)	36 (24)	28 (17.7)	0.55
Married-, n (%)	198 (42.7)	70 (44.9)	62 (41.3)	66 (41.8)	0.79
Education-, n (%)					<0.001
None	50 (10.8)	4 (2.6)	14 (9.3)	32 (20.3)	
Up to JHS	208 (44.8)	24 (15.4)	80 (53.3)	104 (65.8)	
SHS to tertiary	68 (14.7)	24 (15.4)	24 (16)	20 (12.7)	
Tertiary	138 (29.7)	104 (66.7)	32 (21.3)	2 (1.3)	
Employment, n (%)					<0.001
Unemployed	56 (12.1)	22 (14.1)	20 (13.3)	14 (8.9)	
Self-employed	148 (31.9)	14 (9)	54 (36)	80 (50.6)	
Formal employment	260 (56)	120 (76.9)	76 (50.7)	64 (40.5)	
Biomass exposure, n (%)					<0.001
Low	342 (73.7)	132 (84.6)	96 (64)	114 (72.2)	
Medium–-high	122 (26.3)	24 (15.4)	54 (36)	44 (27.8)	
Weight, kg	60±13.2	68±13.3	64.4±7.3	65.8±14	0.204
BMI, kg/m^2	24.8±5	25.4±4.7	23.7±4.4	25.3±5.7	0.061
BMI categories, n (%)					0.024
Underweight	34 (7.4)	6 (3.9)	16 (10.7)	12 (7.6)	
Normal	238 (51.5)	70 (45.5)	94 (62.7)	74 (46.8)	
Overweight	116 (25.1)	46 (29.9)	18 (12)	52 (32.9)	
Obese	74 (16)	32 (20.8)	22 (14.7)	20 (12.7)	
Current CD4 count, cells/mm^2	405 (273 – 562)		403 (253 – 583)	430 (327 – 534)	0.804
Spirometric measurements					
FEV1, l	2.2±0.6	2.4±0.6	2.3±0.7	1.9±0.9$^{*\#}$	<0.001
FVC, l	2.7±0.7	3±0.7	2.9±0.8	2.3±0.5$^{*\#}$	<0.001

Deleted Cells

Deleted Cells

FEF_{25-75}, l/min	2.3±0.9	2.5±0.9	2.4±1	1.9±0.8*#	<0.001
FEV1/FVC, %	80.3±6.9	81.3±5.7	80±7.4	79.4±7.3	0.129
Abnormal spirometry by LLN, n (%)					
Obstructive	4446 (9.59)	14 (98 (5.1)	14 (9.316 (10.7)	16 (10.122 (13.9)	0.994026
Restrictive	180 (3824 (19.8)	50 (32.126 (16.7)	48 (3220 (13.3)	82 (51.936 (22.8)	0.013087
Abnormal spirometry by a fixed ratio, n (%)					
UndefinedObstructive	34 (7.326 (5.6)	10 (4 (2.6.4)	10 (6.78 (5.3)	14 (8.9)	0.811052
RestrictiveTotal	222 (4792 (19.8)	64 (4128 (17.9)	62 (4126 (17.3)	96 (60.838 (24.1)	0.018258
Respiratory symptoms, n (%)	166 (35.8)	30 (19.2)	60 (40)	76 (48.1)	<0.001

HAART, highly activecART, combination antiretroviral therapy; JHS, junior high school; SHS, senior high school; BMI, body mass index; FEV1, forced expiratory volume in 1 second; FVC, forced vital capacity.

*: p<0.05 compared to non-HIV
#: p< 0.05 compared to untreated HIV patients

Respiratory symptoms

Among the entire study participants, 35.8% reported the presence of at least one respiratory symptom, with a high proportion in HAARTcART-treated and HAARTcART-naïve HIV patients compared to non-HIV participants (48.1% vs 40% vs 19.2 respectively, p<0.001). Phlegm production and regular cough were commonly reported in 26.4% and 19% of the entire study participants respectively, with a high proportion in HAARTcART-treated and HAARTcART-naïve HIV patients compared to non-HIV participants (phlegm: 35.4% vs 26.7% vs 6.4%, p<0.001; regular coughs: 30.4% vs 20% vs 11.5%, p<0.001 respectively). Similar trends were observed in participants who reported having dyspnoea on exertion and frequent wheeze symptoms (Fig. 1). Increasing age, being unemployed, former cigarette smoking, exposure to a high-medium level of biomass, being HIV patients with or without HAARTcART treatment and having basic or no education was associated with increased odds of having respiratory symptoms in unadjusted regression models. In the adjusted regression

models, exposure to medium-high ~~level~~levels of biomass, being ~~HAART~~ a cART-treated HIV or ~~HAART~~cART-naïve HIV patient were associated with increased odds of having respiratory symptoms (Table 2).

Fig. 1 Prevalence of respiratory symptoms in study participants by the HIV status

Table 2. Factors associated with respiratory symptoms from logistic regression analyses

	Unadjusted model		Adjusted model	
	OR (95% CI)	p	OR (95% CI)	p
Age~~-~~, yrs	1.02 (1.01 – 1.03)	0.007	1 (0.98 – 1.02)	0.734
Females~~-~~, n	1.32 (0.87 – 1.99)	0.189	1.14 (0.69 – 1.88)	0.623
Unmarried	1.34 (0.91 – 1.97)	0.14	1.18 (0.76 – 1.86)	0.462
Alcohol intake	0.97 (0.62 – 1.54)	0.909	1 (0.59 – 1.68)	0.986
Smoking (reference: never smoked)				
Current ~~smoking~~	1.84 (0.68 – 4.98)	0.233	1.52 (0.5 – 4.64)	0.463
Former	2.75 (1.12 – 5.08)	<0.001	1.94 (1 – 4.65)	0.071
Medium/high biomass exposure	2.72 (1.12 – 4.57)	0.008	1.87 (1.~~05~~07 – 3.49)	0.~~017~~037
HIV status: reference= non-HIV				
~~Treatment~~ cART-naïve	2.8 (1.67 – 4.69)	<0.001	2.03 (1.1 – 3.74)	0.023
~~HAART treatment~~cART-treated	3.89 (2.35 – 6.46)	<0.001	2.61 (1.32 – 5.17)	0.006
Employment				
Self-employed	1.4 (0.92 – 2.13)	0.118	0.83 (0.5 – 1.36)	0.451
Unemployed	1.88 (1.05 – 3.38)	0.035	1.78 (0.91 – 3.48)	0.091
Education: reference= tertiary				
None	3.32 (1.67 – 6.6)	<0.001	2.22 (0.89 – 5.51)	0.086
Basic/JHS	3.21 (1.97 – 5.22)	<0.001	1.79 (0.89 – 3.62)	0.102
SHS/Tech	0.93 (0.46 – 1.91)	0.85	0.62 (0.27 – 1.43)	0.263
~~BMI (unit change)~~	~~1 (0.95 – 1.03)~~	~~0.587~~		
BMI categories				

Formatted Table

Inserted Cells

Formatted Table

Formatted Table

Formatted Table

	Underweight	1.85 (0.9 – 3.81)	0.095	1.58 (0.73 – 3.42)	0.25
	Overweight	0.68 (0.42 – 1.1)	0.116	0.6 (0.35 – 1.03)	0.062
	Obese	0.79 (0.45 – 1.37)	0.789	0.82 (0.44 – 1.51)	0.517

~~HAART, highly active~~cART, combination antiretroviral therapy; JHS, junior high school; SHS, senior high school; BMI, body mass index.

Formatted: Left, Line spacing: Multiple 1.15 li

Determinants of abnormal spirometric patterns

In logistic regression analyses, being female, unmarried, a former smoker, unemployed, having no formal education, underweight and having ~~a~~ respiratory ~~symptom~~symptoms were associated with increased odds of having OSP in the unadjusted models. In the adjusted models, female gender, former cigarette smoking, medium-to-high biomass exposure, presence of respiratory symptoms, unemployed and being underweight were associated with increased odds of having OSP (Table 3). The ~~determinant~~determinants of OSP among various categories of participants based on HIV status ~~is~~are shown in the supplementary attachment (Table S1 – S3).

Table 3. Factors associated with an obstructive spirometric pattern from logistic regression analyses

Formatted Table

	Unadjusted model		Adjusted model	
	OR (95% CI)	p	OR (95% CI)	p
Age	0.~~99~~79 (0.~~97~~57 – 1.~~02~~07)	0.~~555~~745	0.~~99~~91 (0.~~96~~73 – 1.~~02~~41)	0.~~888~~576
Females	3.~~38~~18 (1.~~39~~24 – 8.~~17~~85)	0.005	2.~~88~~46 (1.~~06 – 7.83~~09 – 5.13)	0.~~038~~031
Unmarried	2.81 (1.35 – 5.83)	0.006	2.~~1~~31 (0.~~93 – 4.73~~86 – 5.19)	0.~~075~~125

Alcohol intake			0.53 (0.22 – 1.3)	0.166	0.669 (0.2319 – 1.59489)	0.303631
Smoking (Reference: never smoked)Current smoking				1.38 (0.3 – 6.28)	0.676	1.87 (0.36 – 9.75) 0.457
Medium/high biomass exposure	Current 1.69 (0.89 – 3.25)	0.114	3.38 (1.38 – 8.33(0.3 – 6.28)	0.008676	1.87 (0.36 – 9.75)	0.457
Respiratory symptoms	Former		2.3567 (1.2613 – 4.407)	<0.008002	2.09 (1.01 – 4.3692 (1.04 – 3.89)	<0.049001
Medium/high biomass exposureRespiratory symptoms			0.96 (0.62 – 1.4849 (1.13 – 3.25)	0.837004	0.69 (0.42 – 3.07 (1.1416 – 8.73)	0.151019
Respiratory symptoms HIV status: reference= non-HIV			2.35 (1.26 – 4.4)	0.008	1.89 (1.11 – 5.08)	0.029
HIV status (reference: non-HIV)				Treatment naïve	1.04 (0.48 – 2.27)	0.913 0.89 (0.33 – 2.38) 0.809
	HAART treatmentcART-naïve		1.142.24 (0.54 – 2.4393 – 5.4)	0.729073	1.229 (0.3969 – 3.68)	0.751548
Employment	cART-treated		3.08 (1.33 – 7.15)	0.009	1.79 (1.03 – 4.18)	0.037
Employment			Self-employed	1.25 (0.61 – 2.56)	0.535	0.99 (0.41 – 2.43) 0.991
	Self-employedUnemployed		2.61 (0.51 (0.23 – 1.15 – 5.9417)	0.022112	0.94 (0.43 – 2.51)3.26 (1.19 – 8.95)	0.022481

Deleted Cells
Deleted Cells
Deleted Cells
Inserted Cells
Inserted Cells
Inserted Cells
Inserted Cells
Deleted Cells
Deleted Cells
Inserted Cells
Deleted Cells

Education: reference= tertiary	Unemployed	2.46 (1.15 – 5.23)	0.02	3.26 (1.19 – 8.95)	0.042
Education (reference: tertiary)			None	2.62 (1.06 – 6.53)	0.038 1.61 (0.38 – 6.75) 0.515
	NoneBasic/JHS	0.88 (0.4 –2.11 (1.9108 – 6.71)	0.738044	1.72 (0.31 (0.14 – 1.1479 – 6.04)	0.07533
	Basic/JHSSHS/Tech	0.91 (0.43 – 1.02 (0.36 – 2.849)	0.632	0.4577 (0.1232 – 1.6926)	0.235257
BMI (unit change)	SHS/Tech0.94 (0.87 – 1.01)	1.02 (0.07836 – 2.84)		0.63 (0.41 – 1.73)	0.358
BMI categories					
	Underweight	5.09 (23.11 – 12.28(1.27 – 10.21)	<0.001	5.083.01 (1.85 – 13.812 – 10.16)	0.002026
	Overweight	1.1509 (0.5154 – 2.5828)	0.729691	0.9698 (0.3959 – 2.3771)	0.926864
	Obese	1.0712 (0.4153 – 2.8278)	0.878748	1.1706 (0.49 – 4– 3.39.02)	0.77877

HAART, highly activecART, combination antiretroviral therapy; JHS, junior high school; SHS, senior high school; BMI, body mass index.

Concerning RSP, female gender, former smoking, medium-to-high biomass exposure and being HAARTa cART-treated HIV patient were associated with increased odds in unadjusted logistic regression models. In multivariable logistic regression models, age, female gender, being unmarried, previous cigarette smoking and medium-to-high biomass exposure and being self-employed or unemployed were associated with increased odds of restrictive lung pattern

Inserted Cells
Inserted Cells
Deleted Cells
Deleted Cells
Inserted Cells

(Table 4). The determinants of RSP among various patient groups are shown in the supplementary attachment (Tables S4 – S6).

Table 4. Factors associated with a restrictive spirometric pattern from logistic regression analyses

	Unadjusted model		Adjusted model	
	OR (95% CI)	p	OR (95% CI)	p
Age	0.~~99~~97 (0.~~98~~93 – 1.01)	0.~~438~~061	0.97 (0.~~95 – 0.99~~65 – 1.29)	0.~~014~~114
Females	~~2~~1.67 (1.09 ~~(1.29 –~~ 3.~~4~~42)	0.~~003~~013	~~2 (~~1.~~11 – 3.59~~74 (1.05 – 4.29)	0.~~021~~041
Unmarried	0.75 (0.5 – 1.14)	0.18	~~2~~1.31 (~~1.38~~0.68 – 3.88)	0.~~001~~741
Alcohol intake	1.43 (0.88 – 2.310	0.151	1.68 (0.96 – 2.95)	0.069
Smoking (reference: never)				
Current ~~smoking~~	0.4 (0.09 – 1.78)	0.229	0.42 (0.09 – 2.95)	0.283
Former	3.07 (1.64 – 8.36)	<0.001	2.31 (1.27 – 6.77)	<0.001
Medium/high biomass exposure	2.~~89~~15 (1.~~63~~13 – 5.~~14~~37)	<0.001	~~2.18 (~~1.~~11 – 4.29~~58 (1.06 – 5.37)	0.~~024~~043
HIV status: reference= non-HIV				
~~Treatment~~ cART-naïve	0.89 (0.53 – 1.~~14 (0.66 – 3.35~~73)	0.~~647~~888	1.67 (0.84 – 3.33)	0.144
~~HAART treatment~~cART-treated	1.75 (1.04 – 2.~~01 (1.21 – 3.35~~59)	0.~~007~~016	4.89 (2.15 – 11.11)	<0.001
Employment				
Self-employed	0.72 (0.44 – 1.17)	0.182	0.~~43~~73 (0.23 – ~~0~~1.79)	0.~~007~~407
Unemployed	1.83 (1 – 3.34)	0.05	2.06 (~~1.03~~0.92 – 4.14)	0.~~041~~061
Education: reference= tertiary				
None	1.89 (0.99 – 3.73)	0.067	2.12 (0.8 – 5.65)	0.131
Basic/JHS	0.99 (0.61 – 1.62)	0.979	0.97 (0.45 – 2.09)	0.94
SHS/Tech	0.49 (0.23 – 1.06)	0.069	0.43 (0.17 – 1.08)	0.071
~~BMI (unit change)~~	~~0.99 (0.95 – 1.03)~~	~~0.662~~		
BMI categories				
Underweight	0.84 (0.36 – 1.94)	0.678	1.31 (0.52 – 3.29)	0.562
Overweight	0.95 (0.57 – 1.57)	0.837	0.65 (0.34 – 1.16)	0.148

Formatted Table

Inserted Cells

Formatted Table

Formatted Table

Formatted Table

	Obese	0.87 (0.48 – 1.6)	0.661	0.71 (0.36 – 1.14)	0.333

~~HAART, highly active~~cART, combination antiretroviral therapy; JHS, junior high school; SHS, senior high school; BMI, body mass index.

Formatted: Left, Line spacing: Multiple 1.15 li

Association between respiratory symptoms and abnormal spirometric patterns

In the entire study participants with OSP, 14 (31.8%) reported having regular cough, 16 (36.4%) reported ~~of~~ regular phlegm production, and 12 (16.7%) reported frequent wheezing or dyspnoea on exertion. Among the participants with RSP, those reporting regular cough or frequent wheeze or dyspnoea on exertion were 24 (16.7) while 34 (23.6%) reported regular phlegm production. In logistic regression analyses, regular cough, frequent wheeze and dyspnoea on exertion were associated with increased odds of having OSP in crude and adjusted models (Fig. 2). Participants with RSP had increased odds of frequent wheeze and dyspnoea on exertion in unadjusted logistic regression analysis, but there was no association between respiratory symptoms and RSP in multivariable analyses (Fig. 3).

Fig. 2~~.~~ Association between respiratory symptoms and obstructive ~~pulmonary~~spirometric pattern from unadjusted (left) and adjusted (right) logistic regression models.

Fig. 3~~.~~ Association between respiratory symptoms and restrictive ~~pulmonary~~spirometric pattern from unadjusted (left) and adjusted (right) logistic regression models.

Discussion

Major findings

This is the first study to report the prevalence of abnormal spirometry in HIV patients in Ghana. The major findings of the study were 1) The prevalence of OSP was similarhigher among the HIV patient groups andcompared to non-HIV controls, whereas the prevalence of RSP is higher in HIV patients on HAART treatment compared to treatment naïve HIV patients and non-HIV controls, similar among the study groups; 2) respiratory symptoms were common among HIV patients compared to non-HIV controls, and were associated with abnormal spirometry; and 3) the major determinants of OSP were female gender, former cigarette smoking, exposure to medium-to-high levels of biomass, presence of a respiratory symptom, unemployment and underweight and that of RSP were age, female gender, being unmarried, medium-to-high biomass exposure and being self-employed or unemployed.

Abnormal spirometric patterns.

We did not find any difference in theThe high prevalence of OSP among the HIV patient groups and non-HIV controls. Few studies have compared the prevalence of OSP in HAARTcART-treated HIV patients to that of HAART-naïve HIV patients and non-HIV controls.in our study is consistent with studies from other countries in sub-Saharan Africa. In the South African population, van Riel *et al* reported a higher prevalence of OSP in HAARTcART-treated HIV patients (34.2%) compared to HAARTcART-naïve HIV patients (11.2%). However, they used a cut-off of 20%-LLN instead of the globally accepted 5%-LLN, thatwhich was used to characterize OSP in our study. This, and this may explain the disparity usually high prevalence of OSP reported in the prevalence of OSP in our study with that of van Riel *et al*that particular study [17]. In agreement withcontrast to the findings of our study, Pefura-Yone *et al* reported that there was no difference in the prevalence of OSP between HIV patients and non-HIV controls using the LLN cut-off. However, the prevalence of OSP in their HIV patients and non-HIV controls were lower than what is reported in our studyprevalences of OSP, which was similar among the HIV patients and non-HIV controls using the LLN cut-off. [16]. Other cross-

sectional studies in HIV patients in sub-Saharan AfricaSSA have reported OSP prevalence between 3.1 – 15.4% [9, 136, 10, 14-17], and a metanalysis reported the prevalence of OSP in HIV patients from 30 observational studies involving 151, 686 participants around the globe to be between 5.6% and 10.6% [6][7], similar to what was found in this study. Few studies have compared the prevalence of OSP in cART-treated HIV patients to that of cART-naïve HIV patients and non-HIV controls.

We found that the prevalence of RSP was similar among HAARTcART-naïve HIV patients and non-HIV controls, but more common in HAARTcART-treated HIV patients. Most spirometry studies conducted in HIV patients and other populations in SSA do not report the prevalence and associated factors of RSP, limiting our ability to compare the prevalence of RSP found in our study to other spirometry studies in SSA. However,The patterns of abnormal spirometry that were reported in our study differed from what was reported by Drummond *et al* reported in HIV patients in the United States. He found that, outin a total of 37% of HIV patients who had abnormal spirometry, 10% hadthere was a high prevalence of OSP (27%) compared to RSP while 27% had OSP (10%) [89], a pattern contrary to what was observed in our study population with RSP higher than OSP. The prevalence of RSP observed in our non-HIV controls (32.1%) was similar to 36% reported by Schultz *et al* in Brazilians [22], but very high compared to 7 – 14% reported in the general US population [8],. The prevalence of RSP observed in our non-HIV controls (16.7%) was similar to 7 – 14% reported in the general US population [9], but higher than 5.9% reported in the older population [23] and 4.7% reported in Peruvian adults [24]., and far lower than to 36% reported by Schultz *et al* in Brazilians [25]. The variation in the prevalence of abnormal spirometric patterns may be due to different reference equations applied in various studies [25] and the fact that various insults may affect pulmonary health across various geographical locations.

The accuracy of the definition of RSP used in this and many other studies, FVC<80% predicted and FEV_1/FVC>LLN, in diagnosing RSP hashave been reported to be poor with low sensitivity but high negative predictive value to exclude patients without restrictive lung disease [25]. On the other hand, some study[26]. On the other hand, some studies reported that using FVC<80% predicted alone, without FEV_1/FVC>LLN, improves specificity and negative prediction of diagnosing restrictive lung disease [11].[12]. These inconsistent definitions of RSP in literature and the failure of current guidelines to address this have resulted in the underappreciation of RSP values in spirometric studies. However, the utility and importance of RSP have been highlighted in some studies as a predictor of cardiovascular morbidity and mortality. For instance, the Cardiovascular Health Study reported that RSP is a significant predictor of CVD mortality and non-CVD mortality in older Caucasians [25].[26]. Similarly, the TESOAD study reported that, although consistent RSP is found in 50% of patients with one-time spirometry, it is associated with an increased risk of all-cause mortality, and specifically, an increased risk of dying from diabetes and heart diseases [12]. Therefore, there is a need for further investigation into the relevance of[13]. Therefore, there is a need for further investigation into the relevance of the high burden of RSP found in HIV patients and non-HIV controls in our study to characterize them properly to prevent future CVD events and deaths.

Determinants of abnormal spirometry

The female gender was associated with an increased likelihood of having abnormal spirometry in our study. This may be because of a high proportion of females in our study participants (67.2%). A systematic review has shown that females are more likely to report asthma and airway obstruction compared to men, but abnormal spirometry is commonsimilar in both genders [26, 27, 28]. Unlike many studies that reported an association between cigarette smoking and airway obstruction, we did not find any association between current smoking and abnormal spirometry, possibly due to the low proportion of smokers in all our study participants

(3.4%), but higher in HIV patient groups. The prevalence of smoking in our study is similar to that of the general Ghanaian population (4.85%) as reported by the 2014 Ghana Demographic Health Survey [28]. However, in agreement with the findings of other studies, biomass exposure was associated with abnormal spirometry in our participants [13, 15]. Biomass fumes contain about 50 air pollutants, with significant exposure similar to regular cigarette smoking [29]. These pollutants can irritate the respiratory tract by causing inflammation and tissue injury such as the excessive release of matrix metalloproteinases and increased hyaluronidase activity [30] that damage the lungs. We did not find any association between the HAART regimen used, CD4 cell count and abnormal spirometry, similar to what was reported in other HIV studies [8, 13, 15]. The association between female sex and abnormal spirometry found in our study may be due to the high representation of females in our study population (67.2%). We found an association between unemployment and OSP, consistent with what has been reported in multicentre studies like BOLD [29] and PLATINO [30] studies. This may be due to the debilitating nature of airway obstruction that may lead to cessation of work in affected patients [29, 30]. Unlike many studies that reported an association between cigarette smoking and airway obstruction, we did not find any association between current smoking and abnormal spirometry, possibly due to the low proportion of smokers in all our study participants (3.4%), but higher in HIV patient groups. Analysis of Demographic Health Surveys of 25 countries in Africa indicated that HIV patients with low socioeconomic status, that is the poors, less educated and manual workers, are more likely to smoke [31]. The prevalence of smoking in our study is similar to that of the general Ghanaian population (4.85%) as reported by the 2014 Ghana Demographic Health Survey [32]. On the other hand, previous smoking was associated with abnormal spirometric patterns and this is consistent with other studies [9, 25]. However, in agreement with the findings of other studies, biomass exposure was associated with abnormal spirometry in our participants [6, 14]. Biomass fumes contain about 50 air pollutants,

with significant exposure similar to regular cigarette smoking [33]. These pollutants can irritate the respiratory tract by causing inflammation and tissue injury such as the excessive release of matrix metalloproteinases and increased hyaluronidase activity [34] that damage the lungs. We did not find any association between the cART regimen used, CD4 cell count and abnormal spirometry, similar to what was reported in other HIV studies [6, 9, 14].

Respiratory symptoms

The prevalence of respiratory symptoms in our HIV population is similar to what has been reported in other sub-Saharan African populations. In urban Ugandan HIV patients, Ddungu *et al* reported the prevalence of any respiratory symptom to be 45%, with 30% reporting chronic cough, 21% reporting frequent phlegm production and wheezing and 26% reporting dyspnoea [14][15]. Similarly, in Cameroonian HIV patients, Pefura-Yone *et al* reported the prevalence of any respiratory symptom to be 47.5% while that of cough and dyspnoea were 25.5% and 36.4% respectively [16]. Contrary to the finding of our study, the prevalence of respiratory symptoms reported in Nigerian HIV patients was low (17%) [13]Contrary to the finding of our study, the prevalence of respiratory symptoms reported in Nigerian HIV patients was low (17%) [14] and HIV patients in South Africa had <4% of any respiratory symptoms [17]. Systematic review and metanalysismeta-analysis of studies conducted in developed countries have similarly reported a high prevalence of respiratory symptoms irrespective of access to HAARTcART and high viremic control [7]. In our study, we found that in addition to having HIV, increasing age, exposure to medium-high levels of biomass fumes, unemployment and no/basic education were predictors of respiratory symptoms.[8]. In this study, we found that HIV status and medium-high biomass exposure were associated with respiratory symptoms in the adjusted model. Respiratory symptoms are known to be common with increasing age due to prolonged exposure to environmental toxins and decreased physiological capacity [10].[11]. Our population has a low smoking burden but high biomass exposure and environmental

pollution common in the peri-urban area might be responsible for the high prevalence of respiratory symptoms [29].[33]. With the popularity of spirometry for screening airway obstruction, respiratory symptoms are being given less attention. However, the symptoms experienced by HIV patients indicates functional status and quality of life, even if they correlate poorly with spirometry [78].

Field Code Changed

Limitations of the study

This study was conducted in a single primary healthcare facility in peri-urban Ghana, and hence, the findings may differ from the urban and rural populations, as well as other healthcare facilities in a different stratum. We cannot infer causation from the findings of the study due to the cross-sectional design. It may be possible that some of our participants may have had abnormal spirometry, especially RSP, before HIV infection. The data on chronic respiratory symptoms may be affected by recall bias, which may lead to the misclassification of some study participants. We did not assess important parameters like physical activity level, cART adherence, previous AIDS events and viral suppression which are reported to have an impact on pulmonary health. In this study, airway obstruction was defined by single spirometry. It has been that about 20% and 28% of reported, in patients initially diagnosed with OSP COPD using spirometry, 20% reversed from OSP to normal patternspirometry after one year of diagnosis, and this increased to 28% after two years, respectively.. Among patients diagnosed with normal spirometric patterns, 11% and 17% recordedprogressed to OSP after one andyear and this increased to 17% after two years, respectively [31]. Therefore, with single spirometry as[35]. Therefore, using a single spirometric measurement in this study, we cautiously avoided categorising our study participants as having COPD to avoid this misclassification. In addition, we used the GLI reference equation based on African Americanequations for the Cameroonian population for computingto compute prediction values and LLN for spirometric values, which yield different outcomes compared to the GLI reference equations for African-Americans. The

GLI equation is reported to significantly underestimate FEV1 and FVC by 9% in the Cameroonian population ~~[32], implying that the performance of GLI equations in the SSA adult population may be limited. Therefore, there is a need for deriving locally reference values from the Ghanaian population for accurate interpretation of spirometric values~~[36], implying significant errors in studies conducted in SSA that applied the GLI reference equation.

Conclusion

In conclusion, our study has shown that PLWH without any recent pulmonary infection in a peri-urban area of Ghana, the prevalence of OSP was ~~similar~~high among ~~HAART~~cART-treated HIV ~~and HAART~~compared to cART-naïve HIV patients ~~compared to the~~ and non-HIV control. ~~However, the~~The prevalence of RSP ~~is higher in HIV patients on HAART treatment~~was similar among various study groups. This study has also shed light on the various predictors of spirometric abnormalities with the hope that future studies may investigate the possibility of using interventions targeted at the various risk factors to reduce pulmonary dysfunction in HIV patients.

Supporting information

Table S1. Determinants of obstructive lung pattern in ~~HAART~~cART-treated HIV patients
Table S2. Determinants of obstructive lung pattern in ~~HAART~~cART-naïve HIV patients
Table S3. Determinants of obstructive lung pattern in ~~treatment~~ non-HIV controls
Table S4. Determinants of restrictive lung pattern in ~~HAART~~cART-treated HIV patients
Table S5. Determinants of restrictive lung pattern in ~~HAART~~cART-naïve HIV patients
Table S6. Determinants of restrictive lung pattern in non-HIV controls
Table S7. Respiratory symptoms and spirometric abnormalities among ~~HAART~~cART-treated HIV patients

Acknowledgements: Our sincere thanks go to the staff and patients of Atua Government Hospital who assisted in data collection, especially Mr Samuel Essel and Mrs Nneka Essel.

Funding: There was no funding for this study.

Availability of data and materials: The dataset supporting the conclusion of this study is available for systematic review and meta-analysis upon request from the corresponding author.

Contributions:

KY contributed to the conception, design, data analysis, and drafting of the manuscript and bears the primary responsibility for the content of the manuscript.

LM collected the data and revised the manuscript.

KBA was involved in the supervision and revision of the manuscript.

All the authors read and approved the content of the manuscript.

Ethics approval and consent to participate

The study was conducted in conformity with the Helsinki Declaration on Human Experimentation, 1964 with subsequent revisions, latest Seoul, October 2008. Ethical approval for the study was granted by the Korle Bu Teaching Hospital Institutional Review Board (Protocol ID number: KBTH-IRB/000131-2018). All the participants provided voluntary written consent before being recruited into the study.

Consent for publication: Not applicable

Competing interests: The authors declare that they have no competing interests.

Reference

1. James SL, Abate D, Abate KH, Abay SM, Abbafati C, Abbasi N, Abbastabar H, Abd-Allah F, Abdela J, Abdelalim A: **Global, regional, and national incidence, prevalence, and years lived with disability for 354 diseases and injuries for 195 countries and territories, 1990–2017: a systematic analysis for the Global Burden of Disease Study 2017**. *The Lancet* 2018, **392**(10159):1789-1858.
2. Roth GA, Abate D, Abate KH, Abay SM, Abbafati C, Abbasi N, Abbastabar H, Abd-Allah F, Abdela J, Abdelalim A: **Global, regional, and national age-sex-specific mortality for 282 causes of death in 195 countries and territories, 1980–2017: a systematic analysis for the Global Burden of Disease Study 2017**. *The Lancet* 2018, **392**(10159):1736-1788.
3. Ghana AIDS Commission: **National and Sub-National HIV and AIDS Estimates and Projections 2020 Report**. In., vol. 2020. Accra: Ghana AIDS Commision; 2021.
4. Teeraananchai S, Kerr S, Amin J, Ruxrungtham K, Law M: **Life expectancy of HIV-positive people after starting combination antiretroviral therapy: a meta-analysis**. *HIV medicine* 2017, **18**(4):256-266.

Formatted: Left, Space After: 0 pt, Line spacing:

Formatted: Font: Calibri, 9 pt

Formatted: Left, Line spacing: single

Formatted: Left, Space After: 0 pt, Line spacing:

5. Yang H-Y, Beymer MR, Suen S-c: **Chronic Disease Onset Among People Living with HIV and AIDS in a Large Private Insurance Claims Dataset**. *Scientific Reports* 2019, **9**(1):18514.

~~6~~6. Kayongo A, Wosu AC, Naz T, Nassali F, Kalyesubula R, Kirenga B, Wise RA, Siddharthan T, Checkley W: **Chronic Obstructive Pulmonary Disease Prevalence and Associated Factors in a Setting of Well-Controlled HIV, A Cross-Sectional Study**. *COPD: Journal of Chronic Obstructive Pulmonary Disease* 2020, **17**(3):297-305.

7. Bigna JJ, Kenne AM, Asangbeh SL, Sibetcheu AT: **Prevalence of chronic obstructive pulmonary disease in the global population with HIV: a systematic review and meta-analysis**. *The Lancet Global Health* 2018, **6**(2):e193-e202.

~~7~~8. Brown J, Roy A, Harris R, Filson S, Johnson M, Abubakar I, Lipman M: **Respiratory symptoms in people living with HIV and the effect of antiretroviral therapy: a systematic review and meta-analysis**. *Thorax* 2017, **72**(4):355-366.

~~8~~9. Drummond MB, Huang L, Diaz PT, Kirk GD, Kleerup EC, Morris A, Rom W, Weiden MD, Zhao E, Thompson B *et al*: **Factors associated with abnormal spirometry among HIV-infected individuals**. *Aids* 2015, **29**(13):1691-1700.

~~9~~10. Gupte AN, Wong ML, Msandiwa R, Barnes GL, Golub J, Chaisson RE, Hoffmann CJ, Martinson NA: **Factors associated with pulmonary impairment in HIV-infected South African adults**. *PLOS ONE* 2017, **12**(9):e0184530.

~~10~~11. Vaz Fragoso CA, Gill TM: **Respiratory Impairment and the Aging Lung: A Novel Paradigm for Assessing Pulmonary Function**. *The Journals of Gerontology: Series A* 2011, **67A**(3):264-275.

~~11~~12. Venkateshiah SB, Ioachimescu OC, McCarthy K, Stoller JK: **The Utility of Spirometry in Diagnosing Pulmonary Restriction**. *Lung* 2008, **186**(1):19-25.

~~12~~13. Guerra S, Sherrill DL, Venker C, Halonen M, Martinez FD: **Chronic Restrictive Lung Pattern Is Associated with Increased Mortality Risk**. In: *American Thoracic Society 2009 International Conference.* vol. C34. COPD PHENOTYPES. San Diego Convention centre: American Journal of Respiratory and Critical Care Medicine; 2009: A4325.

~~13~~14. Akanbi MO, Taiwo BO, Achenbach CJ, Ozoh OB, Obaseki DO, Sule H, Agbaji OO, Ukoli CO: **HIV Associated Chronic Obstructive Pulmonary Disease in Nigeria**. *J AIDS Clin Res* 2015, **6**(5).

~~14~~15. Ddungu A, Semitala FC, Castelnuovo B, Sekaggya-Wiltshire C, Worodria W, Kirenga BJ: **Chronic obstructive pulmonary disease prevalence and associated factors in an urban HIV clinic in a low income country**. *PLOS ONE* 2021, **16**(8):e0256121.

~~15. Kayongo A, Wosu AC, Naz T, Nassali F, Kalyesubula R, Kirenga B, Wise RA, Siddharthan T, Checkley W: **Chronic Obstructive Pulmonary Disease Prevalence and Associated Factors in a Setting of Well-Controlled HIV, A Cross-Sectional Study**. *COPD: Journal of Chronic Obstructive Pulmonary Disease* 2020, **17**(3):297-305.~~

16. Pefura-Yone EW, Fodjeu G, kengne AP, Roche N, Kuaban C: **Prevalence and determinants of chronic obstructive pulmonary disease in HIV infected patients in an African country with low level of tobacco smoking**. *Respiratory Medicine* 2015, **109**(2):247-254.

17. van Riel SE, Klipstein-Grobusch K, Barth RE, Grobbee DE, Feldman C, Shaddock E, Stacey SL, Venter WDF, Vos AG: **Predictors of impaired pulmonary function in people living with HIV in an urban African setting**. *South Afr J HIV Med* 2021, **22**(1):1252.

~~18~~18. Sidhu MK, Ravindra K, Mor S, John S: **Household air pollution from various types of rural kitchens and its exposure assessment**. *Science of The Total Environment* 2017, **586**:419-429.

19. Ferris BG: **Epidemiology Standardization Project (American Thoracic Society)**. *Am Rev Respir Dis* 1978, **118**(6 Pt 2):1-120.

~~19~~20. Graham BL, Steenbruggen I, Miller MR, Barjaktarevic IZ, Cooper BG, Hall GL, Hallstrand TS, Kaminsky DA, McCarthy K, McCormack MC *et al*: **Standardization of Spirometry 2019 Update. An Official American Thoracic Society and European Respiratory Society Technical Statement**. *American Journal of Respiratory and Critical Care Medicine* 2019, **200**(8):e70-e88.

~~20. Quanjer PH, Stanojevic S, Cole TJ, Baur X, Hall GL, Culver BH, Enright PL, Hankinson JL, Ip MSM, Zheng J *et al*: **Multi-ethnic reference values for spirometry for the 3-95-yr age range: the global lung function 2012 equations**. *European Respiratory Journal* 2012, **40**(6):1324-1343.~~

~~21~~21. Pefura-Yone EW, Balkissou AD, Poka-Mayap V, Djenabou A, Massongo M, Ofimboudem NA, Mayoh-Nguemfo CF, Tsala AG, Hadjara H, Amougou F: **Spirometric reference equations for Cameroonians aged 4 to 89 years derived using lambda, mu, sigma (LMS) method**. *BMC Pulmonary Medicine* 2021, **21**(1):344.

22. van Gemert F, Kirenga B, Chavannes N, Kamya M, Luzige S, Musinguzi P, Turyagaruka J, Jones R, Tsiligianni I, Williams S *et al*: **Prevalence of chronic obstructive pulmonary disease and associated risk factors in Uganda (FRESH AIR Uganda): a prospective cross-sectional observational study**. *Lancet Glob Health* 2015, **3**(1):e44-51.

Formatted: Font: Calibri, 9 pt

Formatted: Font: Calibri, 9 pt

Formatted: Font: Calibri, 9 pt

Formatted: Font: Calibri, 9 pt

Formatted: Font: Calibri, 9 pt

Formatted: Font: Calibri, 9 pt

Formatted: Font: Calibri, 9 pt

Formatted: Font: Calibri, 9 pt

Formatted: Font: Calibri, 9 pt

Formatted: Font: Calibri, 9 pt

Formatted: Font: Calibri, 9 pt

Formatted: Left, Space After: 0 pt, Line spacing:

Formatted: Font: Calibri, 9 pt

Formatted: Font: Calibri, 9 pt

Formatted: Font: Calibri, 9 pt

Formatted: Left, Space After: 0 pt, Line spacing:

~~22. Schultz K, D'Aquino LC, Soares MR, Gimenez A, Pereira CAdC: **Lung volumes and airway resistance in patients with a possible restrictive pattern on spirometry**. *Jornal Brasileiro de Pneumologia* 2016, **42**:341-347.~~

23. Vaz Fragoso CA, Van Ness PH, Murphy TE, McAvay GJ: **Spirometric impairments, cardiovascular outcomes, and noncardiovascular death in older persons**. *Respiratory Medicine* 2018, **137**:40-47.

24. Siddharthan T, Grigsby M, Miele CH, Bernabe-Ortiz A, Miranda JJ, Gilman RH, Wise RA, Porter JC, Hurst JR, Checkley W: **Prevalence and risk factors of restrictive spirometry in a cohort of Peruvian adults**. *Int J Tuberc Lung Dis* 2017, **21**(9):1062-1068.

~~25~~25. Schultz K, D'Aquino LC, Soares MR, Gimenez A, Pereira CAdC: **Lung volumes and airway resistance in patients with a possible restrictive pattern on spirometry**. *Jornal Brasileiro de Pneumologia* 2016, **42**:341-347.

26. Hyatt RE, Cowl CT, Bjoraker JA, Scanlon PD: **Conditions Associated With an Abnormal Nonspecific Pattern of Pulmonary Function Tests**. *CHEST* 2009, **135**(2):419-424.

~~26~~27. Aryal S, Diaz-Guzman E, Mannino DM: **COPD and gender differences: an update**. *Translational Research* 2013, **162**(4):208-218.

~~27~~28. Camp PG, Goring SM: **Gender and the Diagnosis, Management, and Surveillance of Chronic Obstructive Pulmonary Disease**. *Proceedings of the American Thoracic Society* 2007, **4**(8):686-691.

~~28~~29. Grønseth R, Erdal M, Tan WC, Obaseki DO, Amaral Andre FS, Gislason T, Juvekar SK, Koul PA, Studnicka M, Salvi S *et al*: **Unemployment in chronic airflow obstruction around the world: results from the BOLD study**. *European Respiratory Journal* 2017, **50**(3):1700499.

30. Montes de Oca M, Halbert RJ, Talamo C, Perez-Padilla R, Lopez MV, Muiño A, Jardim JRB, Valdivia G, Pertuzé J, Moreno D *et al*: **Paid employment in subjects with and without chronic obstructive pulmonary disease in five Latin American cities: the PLATINO study**. *The International Journal of Tuberculosis and Lung Disease* 2011, **15**(9):1259-1264.

31. Murphy JD, Liu B, Parascandola M: **Smoking and HIV in Sub-Saharan Africa: A 25-Country Analysis of the Demographic Health Surveys**. *Nicotine & Tobacco Research* 2018, **21**(8):1093-1102.

32. Nketiah-Amponsah E, Afful-Mensah G, Ampaw S: **Determinants of cigarette smoking and smoking intensity among adult males in Ghana**. *BMC Public Health* 2018, **18**(1):941.

~~29~~33. Torres-Duque C, Maldonado D, Pérez-Padilla R, Ezzati M, Viegi G: **Biomass Fuels and Respiratory Diseases**. *Proceedings of the American Thoracic Society* 2008, **5**(5):577-590.

~~30~~34. Silva R, Oyarzún M, Olloquequi J: **Pathogenic Mechanisms in Chronic Obstructive Pulmonary Disease Due to Biomass Smoke Exposure**. *Archivos de Bronconeumología (English Edition)* 2015, **51**(6):285-292.

~~31~~35. Schermer TR, Robberts B, Crockett AJ, Thoonen BP, Lucas A, Grootens J, Smeele IJ, Thamrin C, Reddel HK: **Should the diagnosis of COPD be based on a single spirometry test?** *npj Primary Care Respiratory Medicine* 2016, **26**(1):16059.

~~32~~36. Pefura-Yone EW, Balkissou AD, Poka-Mayap V, Djenabou A, Massongo M, Ofimboudem NA, Mayoh-Nguemfo CF, Tsala AG, Hadjara H, Amougou F: **Spirometric reference equations for Cameroonians aged 4 to 89 years derived using lambda, mu, sigma (LMS) method**. *BMC Pulm Med* 2021, **21**(1):344.

Formatted: Font: Calibri, 9 pt

Formatted: Font: Calibri, 9 pt

Formatted: Font: Calibri, 9 pt

Formatted: Font: Calibri, 9 pt

Formatted: Font: Calibri, 9 pt

Formatted: Left, Space After: 0 pt, Line spacing:

Formatted: Font: Calibri, 9 pt

Formatted: Font: Calibri, 9 pt

Formatted: Font: Calibri, 9 pt

Formatted: Font: Calibri, 9 pt

Formatted: Left, Line spacing: single

Formatted: Font: Calibri, 9 pt
