# Supplementary material for: Abnormal spirometric patterns and respiratory symptoms in HIV patients with no recent pulmonary infection in a periurban hospital in Ghana
Source: PLoS One. 2024 Oct 16;19(10):e0273063. doi: 10.1371/journal.pone.0273063 (PMC11482697; doi:10.1371/journal.pone.0273063)
Supplement: Attachment — Submitted filename: PONE-D-22-21464_R4_Annotated .pdf [file pone.0273063.s007.pdf]

PLOS ONE

Abnormal spirometric patterns and respiratory symptoms in HIV patients with no recent pulmonary infection in a periurban hospital in Ghana

--Manuscript Draft--

**Manuscript Number:**	PONE-D-22-21464R4
**Article Type:**	Research Article
**Full Title:**	Abnormal spirometric patterns and respiratory symptoms in HIV patients with no recent pulmonary infection in a periurban hospital in Ghana
**Short Title:**	Spirometric patterns and respiratory symptoms in Ghanaian HIV patients
**Corresponding Author:**	Kwame Yeboah, PhD<br>University of Ghana<br>Legon, Greater Accra GHANA
**Keywords:**	Abnormal spirometric patterns; Respiratory symptoms; HIV; cART; Ghana
**Abstract:**	Background: Human immunodeficiency virus (HIV) infection is associated with chronic airway obstruction, even in patients who have achieved viral suppression from combination antiretroviral treatment (cART). Spirometry is a supplementary test that aids in diagnosing pulmonary dysfunction in people living with HIV.<br>Aim: To compare the prevalence of spirometric abnormalities among cART-treated HIV patients and cART-naïve HIV patients with non-HIV controls with no recent history of pulmonary infection in a peri-urban hospital in Ghana.<br>Methods: In a case-control design, spirometry was performed in 158 cART-treated HIV patients, 150 cART-naïve HIV patients and 156 non-HIV controls. Clinical, sociodemographic data and respiratory symptoms were collected using a structured questionnaire. Spirometric abnormalities were categorised as obstructive (OSP) or restrictive (RSP) spirometric patterns based on the Cameroonian reference equation.<br>Results: The prevalence of OSP was higher in the cART-treated and cART-naïve HIV patients compared to non-HIV controls (13.9% vs 10.7% vs 5.1% respectively, p = 0.026), whereas that of RSP was similar among the study groups. Respiratory symptoms were common among cART-treated and cART-naïve HIV patients compared to non-HIV controls (48.1% vs 40% vs 19.2% respectively, p<0.001). The major factors associated with OSP were female gender [OR (95% CI)=2.46 (1.09 – 5.13), p=0.031], former cigarette smoking [1.92 (1.04 – 3.89), p<0.001], exposure to medium-to-high levels of biomass [3.07 (1.16 – 8.73), p=0.019], presence of a respiratory symptom [1.89 (1.11 – 5.08), p=0.029] and unemployment [3.26 (1.19 – 8.95), p=0.042]. The major determinants of RSP were age, female gender [1.74 (1.05 – 4.29), p=0.041], former cigarette smoking [2.31 (1.27 – 6.77), p<0.001] and medium-to-high biomass exposure [1.58 (1.06 – 5.37), p=0.043].<br>Conclusion: In HIV patients without any recent pulmonary infection in a peri-urban area of Ghana, there was a higher prevalence of OSP among cART-treated and cART naïve HIV patients compared to the non-HIV control. However, the prevalence of RSP was similar among various study groups.
**Order of Authors:**	Kwame Yeboah
	Latif Musah, MPhil
	Kweku Bedu-Addo, PhD
**Opposed Reviewers:**	
**Response to Reviewers:**	I have kindly incorporated the comments of the Reviewer #4 as follows:<br><br>1.Areas for Improvements:<br>There are no major areas for improvement. There are however, a few minor issues the authors need to tackle. These are specified below.<br><br>1) Under “results” in “abstract” (page 2), line 9; review punctuation mark before the word “and”.<br>Response: We have kindly replaced the punctuation (,) with a (;) to make the two phrases distinct.

Powered by Editorial Manager® and ProduXion Manager® from Aries Systems Corporation

2) Under “results” in “abstract” (page 2), line 10; replace the punctuation mark before the word “medium” with the word –“and”.
Response: We have kindly replaced the punctuation mark before the word “medium” with the word “and” as suggested.

3) Under “study participants” in “methods” (page 4), line 6; review the location stated for Atua Government Hospital.
Response: We have kindly corrected the location with the formal name as the the Lower Manya Krobo Municipality.

4) Under “general characteristics of study participants” in “results” (page 7), line 4; review the phrase – “were likely to be” since you already found and know the result.
Response: We have kindly replaced the phrase “were likely to be” with “were mostly”.

5) Under “general characteristics of study participants” in “results” (page 7), line 7; review the phrase – “were likely to be” since you already found and know the result.
Response: We have kindly replaced the phrase “were likely to be” with “were mostly”.

6) Under “general characteristics of study participants” in “results” (page 7), line 7; indicate the proportion allocated to the underweights.
Response: We have kindly indicated the proportion of underweights in the study to be 9.1%

7) Under “general characteristics of study participants” in “results” (page 9), Table 1; indicate with Greek symbols, groups within which significant differences exist in the “obstructive” results under “abnormal spirometry by LLN, n (%)”
Response: We have kindly used asterisks to indicate group difference in abnormal spirometry as were done for other parameters.

8) Under “general characteristics of study participants” in “results” (page 9), Table 1; indicate with Greek symbols, groups within which significant differences exist in results for “Respiratory symptoms, n (%)”
Response: We have kindly used asterisks to indicate group difference in abnormal spirometry as were done for other parameters.

9) Under “Respiratory symptoms” in “results” (page 9), Fig 1; indicate where in the document this “fig 1” could be located as actual figure is omitted.
Response: We have kindly indicated the location in the manuscript that Fig 1 should be inserted at Page 9 line 22. Also, Fig 1 was uploaded separately as a file document as recommended by PLOS One submission guidelines.

10) Under “Association between respiratory symptoms and abnormal spirometric patterns” in “results” (page 13), Fig 2; indicate where in the document this “Fig 2” could be located as actual figure is omitted.
Response: We have kindly indicated the location in the manuscript that Fig 2 should be inserted at Page 14 line 14. Also, Fig 2 was uploaded separately as a file document as recommended by PLOS One submission guidelines.

11) Under “Association between respiratory symptoms and abnormal spirometric patterns” in “results” (page 13), Fig 3; indicate where in the document this “Fig 3” could be located as actual figure is omitted.
Response: We have kindly indicated the location in the manuscript that Fig 3 should be inserted at Page 14 line 16. Also, Fig 3 was uploaded separately as a file document as recommended by PLOS One submission guidelines.

12) Under “Abnormal spirometric patterns” in “discussion” (page 14), line 1; do you intend to say higher or high and in reference to which of the three groups of study?
Response: We have kindly revised the statement as “the prevalence of OSP was higher in both cART-treated and cART-naïve HIV patient groups compared to non-HIV control”.

13) Under “Limitations of study” (page 18), line 17; correct the word- “ad” before the wors –“this”.

	Response: We have kindly corrected the word “ad” to “and”.<br><br>14) Under “Conclusion” in “discussion” (page 18), line 2;- use the superlative of the word –“high”.<br>Response: We have kindly revised the statement as “the prevalence of OSP was higher in both cART-treated and cART-naïve HIV patient groups compared to non-HIV control”.
Additional Information:	
Question	Response
**Financial Disclosure**<br><br>Enter a financial disclosure statement that describes the sources of funding for the work included in this submission. Review the submission guidelines for detailed requirements. View published research articles from *PLOS ONE* for specific examples.<br><br>This statement is required for submission and **will appear in the published article** if the submission is accepted. Please make sure it is accurate.<br><br>**Unfunded studies**<br>Enter: *The author(s) received no specific funding for this work.*<br><br>**Funded studies**<br>Enter a statement with the following details:<br>• Initials of the authors who received each award<br>• Grant numbers awarded to each author<br>• The full name of each funder<br>• URL of each funder website<br>• Did the sponsors or funders play any role in the study design, data collection and analysis, decision to publish, or preparation of the manuscript?<br>• **NO** - Include this sentence at the end of your statement: *The funders had no role in study design, data collection and analysis, decision to publish, or preparation of the manuscript.*<br>• **YES** - Specify the role(s) played.<br><br>* typeset	The authors received no specific funding for this work
**Competing Interests**<br><br>Use the instructions below to enter a competing interest statement for this submission. On behalf of all authors,	The authors have declared that no competing interests exist.

disclose any competing interests that could be perceived to bias this work—acknowledging all financial support and any other relevant financial or non-financial competing interests.<br><br>This statement is required for submission and **will appear in the published article** if the submission is accepted. Please make sure it is accurate and that any funding sources listed in your Funding Information later in the submission form are also declared in your Financial Disclosure statement.<br><br>View published research articles from *PLOS ONE* for specific examples.<br><br>**NO authors have competing interests**<br><br>Enter: *The authors have declared that no competing interests exist.*<br><br>**Authors with competing interests**<br><br>Enter competing interest details beginning with this statement:<br><br>*I have read the journal's policy and the authors of this manuscript have the following competing interests: [insert competing interests here]*<br><br>* typeset	
**Ethics Statement**<br><br>Enter an ethics statement for this submission. This statement is required if the study involved:<br><br>• Human participants<br>• Human specimens or tissue<br>• Vertebrate animals or cephalopods<br>• Vertebrate embryos or tissues<br>• Field research<br><br>Write "N/A" if the submission does not require an ethics statement.	Ethical approval was obtained from the Korle Bu Teaching Hospital Institutional Review Board (Protocol ID number: KBTH-IRB/000131-2018) and all participants provided written voluntary informed consent before joining the study.

Powered by Editorial Manager® and ProduXion Manager® from Aries Systems Corporation

General guidance is provided below. Consult the submission guidelines for detailed instructions. **Make sure that all information entered here is included in the Methods section of the manuscript.**<br><br>**Format for specific study types**<br><br>**Human Subject Research (involving human participants and/or tissue)**<br>• Give the name of the institutional review board or ethics committee that approved the study<br>• Include the approval number and/or a statement indicating approval of this research<br>• Indicate the form of consent obtained (written/oral) or the reason that consent was not obtained (e.g. the data were analyzed anonymously)<br><br>**Animal Research (involving vertebrate animals, embryos or tissues)**<br>• Provide the name of the Institutional Animal Care and Use Committee (IACUC) or other relevant ethics board that reviewed the study protocol, and indicate whether they approved this research or granted a formal waiver of ethical approval<br>• Include an approval number if one was obtained<br>• If the study involved *non-human primates*, add *additional details* about animal welfare and steps taken to ameliorate suffering<br>• If anesthesia, euthanasia, or any kind of animal sacrifice is part of the study, include briefly which substances and/or methods were applied<br><br>**Field Research**<br><br>Include the following details if this study involves the collection of plant, animal, or other materials from a natural setting:<br>• Field permit number<br>• Name of the institution or relevant body that granted permission	
**Data Availability**<br><br>Authors are required to make all data	Yes - all data are fully available without restriction

Powered by Editorial Manager® and ProduXion Manager® from Aries Systems Corporation

underlying the findings described fully available, without restriction, and from the time of publication. PLOS allows rare exceptions to address legal and ethical concerns. See the PLOS Data Policy and FAQ for detailed information.<br><br>A Data Availability Statement describing where the data can be found is required at submission. Your answers to this question constitute the Data Availability Statement and **will be published in the article**, if accepted.<br><br>**Important:** Stating 'data available on request from the author' is not sufficient. If your data are only available upon request, select 'No' for the first question and explain your exceptional situation in the text box.<br><br>Do the authors confirm that all data underlying the findings described in their manuscript are fully available without restriction?	
**Describe where the data may be found in full sentences. If you are copying our sample text, replace any instances of XXX with the appropriate details.**<br><br>• If the data are **held or will be held in a public repository**, include URLs, accession numbers or DOIs. If this information will only be available after acceptance, indicate this by ticking the box below. For example: *All XXX files are available from the XXX database (accession number(s) XXX, XXX.).*<br>• If the data are all contained **within the manuscript and/or Supporting Information files**, enter the following: *All relevant data are within the manuscript and its Supporting Information files.*<br>• If neither of these applies but you are able to provide **details of access elsewhere**, with or without limitations, please do so. For example:<br><br>*Data cannot be shared publicly because of [XXX]. Data are available from the*	The dataset supporting the conclusion of this study is available at the OSF platform with the link: https://osf.io/bg7r2/?view_only=f349189f097c4f00bf46c13933760a30

XXX Institutional Data Access / Ethics Committee (contact via XXX) for researchers who meet the criteria for access to confidential data.<br><br>*The data underlying the results presented in the study are available from (include the name of the third party and contact information or URL).*<br>• This text is appropriate if the data are owned by a third party and authors do not have permission to share the data.<br><br>* typeset	
Additional data availability information:	

Powered by Editorial Manager® and ProduXion Manager® from Aries Systems Corporation

Department of Physiology
University of Ghana Medical School
July 18, 2024

The Editor
PLOS ONE journal

Dear Prof,

Submission of revised manuscript (Manuscript ID: PONE-D-22-21464R3)

Title of Paper: Abnormal spirometric patterns and respiratory symptoms in HIV patients with no recent pulmonary infection in a periurban hospital in Ghana

We sincerely thank the editor for the constructive comments on our revised manuscript. We have accordingly revised the manuscript based on the comments and we believe that the quality has improved, and hopefully to the level that can be accepted for publication.

We have provided a point-by-point explanation of how we addressed the reviewers' comments below.

Disclosure: *The authors received no specific funding for this work. The authors also declare that they have no competing interests as well.*

All the references are accurate and none has been retracted.

We once again thank you for considering our manuscript.

Yours faithfully,

Dr Kwame Yeboah

Manuscript

Click here to access/download;Manuscript;manuscript cleaned.docx

Abnormal spirometric patterns and respiratory symptoms in HIV patients with no recent pulmonary infection in a periurban hospital in Ghana

Kwame Yeboah[1], Latif Musa[1,2], Kweku Bedu-Addo[2]

1. Department of Physiology, University of Ghana Medical School, Accra, Ghana
2. Department of Physiology, School of Medicine and Dentistry, KNUST, Kumasi, Ghana

Corresponding author: Dr Kwame Yeboah, Department of Physiology, University of Ghana Medical School, P O Box 4236, Accra, Ghana.

Email: kyeboah@ug.edu.gh/melvinky@gmail.com.

Short title: Spirometric patterns and respiratory symptoms in Ghanaian HIV patients

Abbreviations: SSA, sub-Saharan Africa; HIV, human immunodeficiency virus; PLWH, people living with HIV; cART, combination antiretroviral therapy; FEV1, forced expiratory volume in 1s; FVC, forced vital capacity; LLN, lower limit of normal; OSP, obstructive spirometric pattern; RSP, restrictive spirometric patterns; GLI, Global Lung Initiative.

Keywords: Abnormal spirometric patterns, Respiratory symptoms, HIV, cART, Ghana

Abstract

Background: Human immunodeficiency virus (HIV) infection is associated with chronic airway obstruction, even in patients who have achieved viral suppression from combination antiretroviral treatment (cART). Spirometry is a supplementary test that aids in diagnosing pulmonary dysfunction in people living with HIV.

Aim: To compare the prevalence of spirometric abnormalities among cART-treated HIV patients and cART-naïve HIV patients with non-HIV controls with no recent history of pulmonary infection in a peri-urban hospital in Ghana.

Methods: In a case-control design, spirometry was performed in 158 cART-treated HIV patients, 150 cART-naïve HIV patients and 156 non-HIV controls. Clinical, sociodemographic data and respiratory symptoms were collected using a structured questionnaire. Spirometric abnormalities were categorised as obstructive (OSP) or restrictive (RSP) spirometric patterns based on the Cameroonian reference equation.

Results: The prevalence of OSP was higher in the cART-treated and cART-naïve HIV patients compared to non-HIV controls (13.9% vs 10.7% vs 5.1% respectively, $p = 0.026$), whereas that of RSP was similar among the study groups. Respiratory symptoms were common among cART-treated and cART-naïve HIV patients compared to non-HIV controls (48.1% vs 40% vs 19.2% respectively, $p<0.001$). The major factors associated with OSP were female gender [OR (95% CI)=2.46 (1.09 – 5.13), $p=0.031$], former cigarette smoking [1.92 (1.04 – 3.89), $p<0.001$], exposure to medium-to-high levels of biomass [3.07 (1.16 – 8.73), $p=0.019$], presence of a respiratory symptom [1.89 (1.11 – 5.08), $p=0.029$] and unemployment [3.26 (1.19 – 8.95), $p=0.042$]. The major determinants of RSP were age, female gender [1.74 (1.05 – 4.29), $p=0.041$], former cigarette smoking [2.31 (1.27 – 6.77), $p<0.001$] and medium-to-high biomass exposure [1.58 (1.06 – 5.37), $p=0.043$].

Conclusion: In HIV patients without any recent pulmonary infection in a peri-urban area of Ghana, there was a higher prevalence of OSP among cART-treated and cART naïve HIV patients compared to the non-HIV control. However, the prevalence of RSP was similar among various study groups.

Introduction

Sub-Saharan Africa (SSA) has the largest population of people living with human immunodeficiency virus (HIV); this is associated with healthcare, socio-economic and developmental challenges [1]. It was reported in 2017 that 71% of the global total number of people living with HIV (PLWH) resides in SSA, with 75% of deaths and 65% of new HIV infections occurring in the region [2]. In Ghana, the prevalence of HIV infection is 1.7% and is concentrated mostly in urban areas [3]. With the introduction of effective combination antiretroviral therapy (cART), PLWH now lives longer due to decreased morbidity and mortality from opportunistic infections [4]. This has increased the susceptibility of PLWH to chronic diseases at greater rates than those observed in HIV-uninfected persons, making HIV infection a manageable chronic disease [5]. Chronic obstructive pulmonary disease (COPD) is one of the major causes of death and disability [2] with an estimated global prevalence of 10.3% [6]. HIV infection has been associated with an increased prevalence of COPD, with some studies suggesting that the prevalence of COPD is 21% higher in PLWH compared to the general population [7]. The high burden of COPD persists in PLWH even after adjustments for risk factors such as smoking, opportunistic infections and injection drug use [4, 8-11].

Spirometry is a simple, effective and reproducible method that can aid the diagnosis of COPD, and the results can be categorized into two major patterns of abnormalities. An obstructive spirometric pattern (OSP) is characterized by a disproportional reduction in the forced expiratory volume in 1 second (FEV1) relative to the forced vital capacity (FVC), leading to a reduction in the FEV1/FVC ratio to less than the lower limit of normal (LLN) of a similar population [12, 13]. A restrictive spirometric pattern (RSP) is defined by a normal FEV1/FVC (>LLN), but an FVC is less than 80% of the predicted value. Both OSP and RSP are associated with increased mortality in the general population [13, 14]. Despite the high prevalence of PLWH in SSA, few studies have reported the prevalence and risk factors for these abnormal

spirometric patterns with recent reports from the FRESH AIR study in Uganda [5, 7, 8, 15-18]. Most of these studies attributed abnormal spirometry in HIV to opportunistic pulmonary infection. This study compared the prevalence of spirometric abnormalities among HIV patients on cART treatment and cART-naïve HIV patients with non-HIV controls with no recent history of pulmonary infection in a peri-urban hospital in Ghana. The study hypothesized that HIV infection would increase the prevalence of spirometric abnormalities compared to those with no HIV infection. Also, the study investigated various factors associated with spirometric abnormalities in PLWH in Ghana.

Methods

Study participants and design

The study used a case-control design, conducted from October 2019 through February 2020, with HIV patients as cases and the controls were non-HIV individuals who visited the facility for voluntary testing of their HIV status. HIV patients were categorized as those on cART management and cART-naïve HIV patients who have not been on cART treatment for more than 4 weeks. The study was conducted at Atua Government Hospital, a 150-bed primary healthcare facility, located in the Lower Manya Krobo Municipality in the Eastern region of Ghana. The Lower Manya Krobo Municipality has a higher prevalence of HIV infection (7%) compared to the national prevalence which stands at 1.7% in 2020 [3]. The hospital has about 3000 HIV patients on its register. Participants younger than 20 years, those diagnosed with severe heart diseases or hospitalized for cardiopulmonary complications within the past year, those with a diagnosis of lung infection or disease from their clinical case notes or answering ~~positively~~ to the question: “Have you been told by your health worker that you have lung disease?” were excluded from the study. In addition, pregnant women, any individual with

mental illness, active or recent (within the past 24 months) tuberculosis or lung infection and those with other conditions contraindicated for spirometry were excluded from the study. Initially, 180 cART-treated HIV patients, 170 cART-naïve controls and 170 non-HIV controls were recruited into the study. However, 22 cART-treated HIV patients, 20 cART-naïve HIV patients and 12 non-HIV controls failed to produce the appropriate manoeuvres required for accurate spirometry recordings, and hence, they were excluded from the analysis. Ethical approval was granted by the Korle Bu Teaching Hospital Institutional Review Board (Protocol ID number: KBTH-IRB/000131-2018) and all participants provided voluntary informed consent before joining the study.

Data collection

A structured questionnaire was used to obtain data on sociodemographic factors like age, gender, lifestyle factors (smoking, alcohol intake), medical history, current HIV medication, occupation, education, marital status and biomass exposure. Medium-high biomass exposure was defined as those who reported using firewood, sawdust, cow dung or corn cubs as a fuel source for work or cooking at home on most days [19]. Respiratory symptoms were assessed using a modified version of the American Thoracic Society respiratory questionnaire, which included the following questions: “Do you usually have coughs without cold for more than 2 months in a year?”, “Do you usually bring up phlegm from your chest when you don’t have a cold?”, “Do you have difficulty breathing when you walk fast, use a staircase or run?” and “Have you ever had an attack of wheezing or whistling in your chest that made you feel short of breath?” [20]. These questions were administered in the local dialect for participants with limited formal education.

Body weight and height were measured using a stadiometer in light clothing with footwear removed, with body mass index (BMI) calculated as weight/height^2 and categorized as

underweight (< 18.5 kg/m^2), normal weight (18.5–24.9 kg/m^2), overweight (25–29.9 kg/m^2), and obese (≥ 30 kg/m^2). Hypertension was defined as self-reported use of antihypertensive treatment and/or systolic blood pressure ≥ 140 mm Hg and/or diastolic blood pressure ≥ 90 mm Hg. Venous blood samples were collected from HIV patients to measure CD4+ lymphocytes.

Spirometry was performed by a qualified technician using a Vitalograph Pneumotrac desktop spirometer model 6800 following the ATS/ERS 2019 standard guidelines [21]. Abnormal spirometric patterns were computed based on fixed ratio and lower limits of normal (LLN) using the Cameroonian spirometric equation [22]. The normal spirometric pattern was defined by FEV1, FVC, and FEV1/FVC all > LLN. Obstructive spirometric pattern (OSP) was defined as FEV1/FVC<0.7 with a percentage predicted FVC>80% using the fixed ratio or FEV1/FVC <LLN with FVC > LLN using the LLN method. A restrictive spirometric pattern was defined as FEV1/FVC>0.7 with a percentage predicted FVC<80% by fixed ratio or FVC < LLN with FEV1/FVC > LLN [22].

Sample size

The sample size required was calculated using two studies conducted in rural Uganda that reported the prevalence of OSP in HIV patients to be 6.2% [7] and that of the non-HIV population to be 16.2% [23]. Therefore, a minimum of 153 participants would be required to achieve a power of 80% at a 95% confidence level with an estimated odds ratio of 2.93.

Data analysis

Data were analysed using IBM SPSS version 28 ~~software.~~ Continuous descriptive data were presented as means and standard deviations for normally distributed data and comparisons between groups were made using one-way ANOVA, while non-normally distributed data were presented as medians and interquartile ranges and comparisons were made using the Kruskal-

Wallis' test. Categorical descriptive data were presented as frequencies and percentages and analysed using the chi-square test. Univariate and multivariable logistic regression models were used to analyse the odds of participants with various socio-demographic and clinical factors having abnormal spirometry and respiratory symptoms. A p-value < 0.05 was considered statistically significant.

Results

General characteristics of study participants

The age range of participants was 20 – 70 years with a mean age of 38.4±13.7 years. There was no difference in mean age among various categories of participants. There was a high proportion of females (67.2%) in this study participants and participants who currently (5.2%) or formerly (15.6%) smoked were mostly HIV patients. HIV patients were mostly unemployed (11%) or self-employed (43.5%) and had no formal education (14.9%) or attended school up to junior high level (59.7%). HIV patients were mostly underweight (9.1%) and had greater biomass exposure from work or home (31.8%). Measurements from spirometry indicated that cART-treated HIV patients had reduced FEV_1, FVC and FEF_{25-75} l/min compared to cART-naïve HIV patients and non-HIV participants. Compared to the non-HIV controls, the prevalence of OSP was high in cART-naïve and cART-treated HIV patients using the LLN. There was no difference in the prevalence of RSP using the LLN. When the fixed ratio was used to determine spirometric abnormalities, there was no difference in the prevalence of OSP and RSP among the study groups (Table 1). The average duration of HIV infection in cART-treated HIV patients was 7.6±4.6 years and the average duration of cART treatment was 7.2±4.5 years. For the cART medication regimen, 94 (59.5%) patients were treated with TDF/3TC/NVP or EFV regimens, 52

(32.9%) patients were on AZT/3TC/NVP or EFV regimens and 12 (7.6%) patients were on LPV/r-based regimens.

Table 1. General characteristics of study participants

	All Participants (n=464)	Non-HIV participants (n=156)	cART-naïve HIV patients (n=150)	cART-treated HIV patients (n=158)	p
Age, years	38.4±13.7	36.7±14.4	38.2±11.6	39±11.4	0.109
Females, n (%)	312 (67.2)	106 (67.9)	84 (56)	122 (77.2)	0.02
Smoking, n (%)					0.029
Current	16 (3.4)	2 (1.3)	4 (2.7)	10 (6.3)	
Former	57 (15.9)	9 (5.8)	22 (14.6)	26 (16.5)	
Never	187 (80.6)	71 (92.9)	124 (82.7)	132 (83.5)	
Alcohol intake, n (%)	102 (22)	38 (24.4)	36 (24)	28 (17.7)	0.55
Married, n (%)	198 (42.7)	70 (44.9)	62 (41.3)	66 (41.8)	0.79
Education, n (%)					<0.001
None	50 (10.8)	4 (2.6)	14 (9.3)	32 (20.3)	
Up to JHS	208 (44.8)	24 (15.4)	80 (53.3)	104 (65.8)	
SHS to tertiary	68 (14.7)	24 (15.4)	24 (16)	20 (12.7)	
Tertiary	138 (29.7)	104 (66.7)	32 (21.3)	2 (1.3)	
Employment, n (%)					<0.001
Unemployed	56 (12.1)	22 (14.1)	20 (13.3)	14 (8.9)	
Self-employed	148 (31.9)	14 (9)	54 (36)	80 (50.6)	
Employed	260 (56)	120 (76.9)	76 (50.7)	64 (40.5)	
Biomass exposure, n (%)					<0.001
Low	342 (73.7)	132 (84.6)	96 (64)	114 (72.2)	
Medium-high	122 (26.3)	24 (15.4)	54 (36)	44 (27.8)	
Weight, kg	60±13.2	68±13.3	64.4±7.3	65.8±14	0.204
BMI, kg/m^2	24.8±5	25.4±4.7	23.7±4.4	25.3±5.7	0.061
BMI categories, n (%)					0.024
Underweight	34 (7.4)	6 (3.9)	16 (10.7)	12 (7.6)	
Normal	238 (51.5)	70 (45.5)	94 (62.7)	74 (46.8)	
Overweight	116 (25.1)	46 (29.9)	18 (12)	52 (32.9)	
Obese	74 (16)	32 (20.8)	22 (14.7)	20 (12.7)	
Current CD4 count, cells/mm^2	405 (273 – 562)		403 (253 – 583)	430 (327 – 534)	0.804
Spirometric measurements					
FEV1, l	2.2±0.6	2.4±0.6	2.3±0.7	1.9±0.9[*#]	<0.001
FVC, l	2.7±0.7	3±0.7	2.9±0.8	2.3±0.5[*#]	<0.001
FEF_{25-75}, l/min	2.3±0.9	2.5±0.9	2.4±1	1.9±0.8[*#]	<0.001
FEV1/FVC, %	80.3±6.9	81.3±5.7	80±7.4	79.4±7.3	0.129
Abnormal spirometry by LLN, n (%)					

Obstructive	46 (9.9)	8 (5.1)	16 (10.7)*	22 (13.9)*	0.026
Restrictive	24 (19.8)	26 (16.7)	20 (13.3)	36 (22.8)	0.087
Abnormal spirometry by a fixed ratio, n (%)					
Obstructive	26 (5.6)	4 (2.6)	8 (5.3)	14 (8.9)	0.052
Restrictive	92 (19.8)	28 (17.9)	26 (17.3)	38 (24.1)	0.258
Respiratory symptoms, n (%)	166 (35.8)	30 (19.2)	60 (40)	76 (48.1)	<0.001

cART, combination antiretroviral therapy; JHS, junior high school; SHS, senior high school; BMI, body mass index; FEV1, forced expiratory volume in 1 second; FVC, forced vital capacity.

*: p<0.05 compared to non-HIV
#: p< 0.05 compared to untreated HIV patients

Respiratory symptoms

Among the entire study participants, 35.8% reported the presence of at least one respiratory symptom, with a higher proportion in cART-treated and cART-naïve HIV patients compared to non-HIV participants (48.1% vs 40% vs 19.2 respectively, p<0.001). Phlegm production and regular cough were commonly reported in 26.4% and 19% of the entire study participants respectively, with a higher proportion in cART-treated and cART-naïve HIV patients compared to non-HIV participants (phlegm: 35.4% vs 26.7% vs 6.4%, p<0.001; regular coughs: 30.4% vs 20% vs 11.5%, p<0.001 respectively). Similar trends were observed in participants who reported having dyspnoea on exertion and frequent wheeze symptoms (Fig. 1). Increasing age, being unemployed, former cigarette smoking, exposure to a high-medium level of biomass, being HIV patients with or without cART treatment and having basic or no education was associated with increased odds of having respiratory symptoms in unadjusted regression models. In the adjusted regression models, exposure to medium-high levels of biomass, being a cART-treated HIV or cART-naïve HIV patient were associated with increased odds of having respiratory symptoms (Table 2).

[*Please insert Fig. 1 here*]

Table 2. Factors associated with respiratory symptoms from logistic regression analyses

	Unadjusted model		Adjusted model	
	OR (95% CI)	p	OR (95% CI)	p
Age (per 1 yr increase)	1.02 (1.01 – 1.03)	0.007	1 (0.98 – 1.02)	0.734
Females (reference = males)	1.32 (0.87 – 1.99)	0.189	1.14 (0.69 – 1.88)	0.623
Unmarried (reference = married)	1.34 (0.91 – 1.97)	0.14	1.18 (0.76 – 1.86)	0.462
Alcohol intake (reference = non-drinkers)	0.97 (0.62 – 1.54)	0.909	1 (0.59 – 1.68)	0.986
Smoking (reference: never smoked)				
Current	1.84 (0.68 – 4.98)	0.233	1.52 (0.5 – 4.64)	0.463
Former	2.75 (1.12 – 5.08)	<0.001	1.94 (1 – 4.65)	0.071
Medium/high biomass exposure (reference = low biomass exposure)	2.72 (1.12 – 4.57)	0.008	1.87 (1.07 – 3.49)	0.037
HIV status (reference = non-HIV)				
cART-naïve	2.8 (1.67 – 4.69)	<0.001	2.03 (1.1 – 3.74)	0.023
cART-treated	3.89 (2.35 – 6.46)	<0.001	2.61 (1.32 – 5.17)	0.006
Employment (reference = Employed)				
Self-employed	1.4 (0.92 – 2.13)	0.118	0.83 (0.5 – 1.36)	0.451
Unemployed	1.88 (1.05 – 3.38)	0.035	1.78 (0.91 – 3.48)	0.091
Education (reference = tertiary)				
None	3.32 (1.67 – 6.6)	<0.001	2.22 (0.89 – 5.51)	0.086
Basic/JHS	3.21 (1.97 – 5.22)	<0.001	1.79 (0.89 – 3.62)	0.102
SHS/Tech	0.93 (0.46 – 1.91)	0.85	0.62 (0.27 – 1.43)	0.263
BMI categories (reference: normal)				
Underweight	1.85 (0.9 – 3.81)	0.095	1.58 (0.73 – 3.42)	0.25
Overweight	0.68 (0.42 – 1.1)	0.116	0.6 (0.35 – 1.03)	0.062
Obese	0.79 (0.45 – 1.37)	0.789	0.82 (0.44 – 1.51)	0.517

cART, combination antiretroviral therapy; JHS, junior high school; SHS, senior high school; BMI, body mass index.

Factors associated with abnormal spirometric patterns

In logistic regression analyses, being female, unmarried, a former smoker, unemployed, having no formal education, underweight and having respiratory symptoms were associated with increased odds of having OSP in the unadjusted models. In the adjusted models, female gender,

former cigarette smoking, medium-to-high biomass exposure, presence of respiratory symptoms, unemployed and being underweight were associated with increased odds of having OSP (Table 3). The associated factors of OSP among various categories of participants based on HIV status are shown in the supplementary attachment (Table S1 – S3).

Table 3. Factors associated with an obstructive spirometric pattern from logistic regression analyses

		Unadjusted model		Adjusted model	
		OR (95% CI)	p	OR (95% CI)	p
Age (per 1 yr increase)		0.79 (0.57 – 1.07)	0.745	0.91 (0.73 – 1.41)	0.576
Females (reference = males)		3.18 (1.24 – 8.85)	0.005	2.46 (1.09 – 5.13)	0.031
Unmarried (reference = married)		2.81 (1.35 – 5.83)	0.006	2.31 (0.86 – 5.19)	0.125
Alcohol intake (reference = non-drinkers)		0.53 (0.22 – 1.3)	0.166	0.69 (0.19 – 1.489)	0.631
Smoking (Reference: never smoked)					
	Current	1.38 (0.3 – 6.28)	0.676	1.87 (0.36 – 9.75)	0.457
	Former	2.67 (1.13 – 4.07)	<0.002	1.92 (1.04 – 3.89)	<0.001
Medium/high biomass exposure (reference = low biomass exposure)		1.49 (1.13 – 3.25)	0.004	3.07 (1.16 – 8.73)	0.019
Respiratory symptoms (reference = no respiratory symptom)		2.35 (1.26 – 4.4)	0.008	1.89 (1.11 – 5.08)	0.029
HIV status (reference: non-HIV)					
	cART-naïve	2.24 (0.93 – 5.4)	0.073	1.29 (0.69 – 3.68)	0.548
	cART-treated	3.08 (1.33 – 7.15)	0.009	1.79 (1.03 – 4.18)	0.037
Employment (reference = employed)					
	Self-employed	0.51 (0.23 – 1.17)	0.112	0.94 (0.43 – 2.51)	0.481
	Unemployed	2.46 (1.15 – 5.23)	0.02	3.26 (1.19 – 8.95)	0.042
Education (reference: tertiary)					
	None	2.11 (1.08 – 6.71)	0.044	1.72 (0.79 – 6.04)	0.533
	Basic/JHS	0.91 (0.43 – 1.9)	0.632	0.77 (0.32 – 1.26)	0.257
	SHS/Tech	1.02 (0.36 – 2.84)	0.583	0.63 (0.41 – 1.73)	0.358
BMI categories (reference = normal)					
	Underweight	3.11 (1.27 – 10.21)	<0.001	3.01 (1.12 – 10.16)	0.026
	Overweight	1.09 (0.54 – 2.28)	0.691	0.98 (0.59 – 2.71)	0.864
	Obese	1.12 (0.53 – 2.78)	0.748	1.06 (0.49 – 4.02)	0.877

cART, combination antiretroviral therapy; JHS, junior high school; SHS, senior high school; BMI, body mass index.

Concerning RSP, female gender, former smoking, medium-to-high biomass exposure and being a cART-treated HIV patient were associated with increased odds in unadjusted logistic regression models. In multivariable logistic regression models, age, female gender, being unmarried, previous cigarette smoking and medium-to-high biomass exposure were associated with increased odds of restrictive lung pattern (Table 4). The associated factors of RSP among various patient groups are shown in the supplementary attachment (Tables S4 – S6).

Table 4. Factors associated with a restrictive spirometric pattern from logistic regression analyses

	Unadjusted model		Adjusted model	
	OR (95% CI)	p	OR (95% CI)	p
Age (per 1 yr increase)	0.97 (0.93 – 1.01)	0.061	0.97 (0.65 – 1.29)	0.114
Females (reference = males)	1.67 (1.09 – 3.42)	0.013	1.74 (1.05 – 4.29)	0.041
Unmarried (reference = married)	0.75 (0.5 – 1.14)	0.18	1.31 (0.68 – 3.88)	0.741
Alcohol intake (reference = non-drinkers)	1.43 (0.88 – 2.310	0.151	1.68 (0.96 – 2.95)	0.069
Smoking (reference: never)				
Current	0.4 (0.09 – 1.78)	0.229	0.42 (0.09 – 2.95)	0.283
Former	3.07 (1.64 – 8.36)	<0.001	2.31 (1.27 – 6.77)	<0.001
Medium/high biomass exposure (reference = low biomass exposure)	2.15 (1.13 – 5.37)	<0.001	1.58 (1.06 – 5.37)	0.043
HIV status (reference= non-HIV controls)				
cART-naïve	0.89 (0.53 – 1.73)	0.888	1.67 (0.84 – 3.33)	0.144
cART-treated	1.75 (1.04 – 2.59)	0.016	4.89 (2.15 – 11.11)	<0.001
Employment (reference = employed)				
Self-employed	0.72 (0.44 – 1.17)	0.182	0.73 (0.23 – 1.79)	0.407
Unemployed	1.83 (1 – 3.34)	0.05	2.06 (0.92 – 4.14)	0.061
Education (reference= tertiary)				
None	1.89 (0.99 – 3.73)	0.067	2.12 (0.8 – 5.65)	0.131
Basic/JHS	0.99 (0.61 – 1.62)	0.979	0.97 (0.45 – 2.09)	0.94
SHS/Tech	0.49 (0.23 – 1.06)	0.069	0.43 (0.17 – 1.08)	0.071
BMI categories (reference = normal BMI)				
Underweight	0.84 (0.36 – 1.94)	0.678	1.31 (0.52 – 3.29)	0.562

	Overweight	0.95 (0.57 – 1.57)	0.837	0.65 (0.34 – 1.16)	0.148
	Obese	0.87 (0.48 – 1.6)	0.661	0.71 (0.36 – 1.14)	0.333

cART, combination antiretroviral therapy; JHS, junior high school; SHS, senior high school; BMI, body mass index.

Association between respiratory symptoms and abnormal spirometric patterns

In ~~the entire~~ study participants with OSP, 14 (31.8%) reported having regular cough, 16 (36.4%) reported regular phlegm production, and 12 (16.7%) reported frequent wheezing or dyspnoea on exertion. Among the participants with RSP, those reporting regular cough or frequent wheeze or dyspnoea on exertion were 24 (16.7) while 34 (23.6%) reported regular phlegm production. In logistic regression analyses, regular cough, frequent wheeze and dyspnoea on exertion were associated with increased odds of having OSP ~~in crude and adjusted models~~ (Fig. 2). Participants with RSP had increased odds of frequent wheeze and dyspnoea on exertion in unadjusted logistic regression analysis, but there was no association between respiratory symptoms and RSP in multivariable analyses (Fig. 3).

[Please insert Fig. 2 here]

[Please insert Fig. 3 here]

Discussion

Major findings

This is the first study to report the prevalence of abnormal spirometry in HIV patients in Ghana. The major findings of the study were: 1) The prevalence of OSP was higher among the higher in both cART-treated and cART-naïve HIV patient groups compared to non-HIV control, whereas the prevalence of RSP is similar among the study groups; 2) respiratory symptoms

were common among HIV patients compared to non-HIV controls and were associated with abnormal spirometry; and 3) the major factors associated with OSP were female gender, former cigarette smoking, exposure to medium-to-high levels of biomass, presence of a respiratory symptom, unemployment and underweight and that of RSP were age, female gender, being unmarried, medium-to-high biomass exposure and being self-employed or unemployed.

Abnormal spirometric patterns.

The higher prevalence of OSP found in cART-treated HIV patients in our study is consistent with studies from other countries in sub-Saharan Africa. In the South African population, van Riel *et al* reported a higher prevalence of OSP in cART-treated HIV patients (34.2%) compared to cART-naïve HIV patients (11.2%). However, that study used a cut-off of 20%-LLN instead of the globally accepted 5%-LLN, which was used to characterize OSP in our study, and this may explain the usually higher prevalence of OSP reported in that particular study [18]. In contrast to the findings of the current study, Pefura-Yone *et al* reported lower prevalences of OSP, which was similar among the HIV patients and non-HIV controls using the 5%-LLN cut-off. [17]. Other cross-sectional studies in HIV patients in SSA have reported OSP prevalence between 3.1 – 15.4% [7, 11, 15-18], and a metanalysis reported the prevalence of OSP in HIV patients from 30 observational studies involving 151, 686 participants around the globe to be between 5.6% and 10.6% [8], similar to what was found in this study. Few studies have compared the prevalence of OSP in cART-treated HIV patients to that of cART-naïve HIV patients and non-HIV controls.

The findings of this study indicated that the prevalence of RSP was similar among cART-naïve HIV patients and non-HIV controls, but more common in cART-treated HIV patients. Most spirometry studies conducted in HIV patients and other populations in SSA do not report the prevalence and associated factors of RSP, limiting our ability to compare the prevalence of

RSP found in our study to other spirometry studies in SSA. The patterns of abnormal spirometry that were reported in this study differed from what was reported by Drummond *et al* reported in HIV patients in the United States. He found that, in a total of 37% abnormal spirometry, the majority of the patients had OSP (27% for OSP, compared to 10% for RSP) [10]. The prevalence of RSP observed in our non-HIV controls (16.7%) was similar to 7 – 14% reported in the general US population [10], but higher than 5.9% reported in the older population [24] and 4.7% reported in Peruvian adults [25], and far lower than to 36% reported by Schultz *et al* in Brazilians [26]. The variation in the prevalence of abnormal spirometric patterns may be due to different reference equations applied in various studies [26] and the fact that various insults may affect pulmonary health across various geographical locations.

The accuracy of the definition of RSP used in this and many other studies, FVC<80% predicted and FEV_1/FVC>LLN, in diagnosing RSP has been reported to be poor with low sensitivity but high negative predictive value to exclude patients without restrictive lung disease [27]. On the other hand, some studies reported that using FVC<80% predicted alone, without FEV_1/FVC>LLN, improves specificity and negative prediction of diagnosing restrictive lung disease [13]. These inconsistent definitions of RSP in literature and the failure of current guidelines to address this have resulted in the underappreciation of RSP values in spirometric studies. However, the utility and importance of RSP have been highlighted in some studies as a predictor of cardiovascular morbidity and mortality. For instance, the Cardiovascular Health Study reported that RSP is a significant predictor of CVD mortality and non-CVD mortality in older Caucasians [27]. Similarly, the TESOAD study reported that, although consistent RSP is found in 50% of patients with one-time spirometry, it is associated with an increased risk of all-cause mortality, and specifically, an increased risk of dying from diabetes and heart diseases [14]. Therefore, there is a need for further investigation into the relevance of the high burden

of RSP found in HIV patients and non-HIV controls in our study to characterize them properly to prevent future CVD events and deaths.

Factors associated with abnormal spirometry

The female gender was associated with an increased likelihood of having abnormal spirometry in this study. A systematic review has shown that females are more likely to report asthma and airway obstruction compared to men, but abnormal spirometry is similar in both genders [28, 29]. The association between female sex and abnormal spirometry found in our study may be due to the high representation of females in our study population (67.2%). This study found an association between unemployment and OSP, consistent with what has been reported in multicentre studies like BOLD [30] and PLATINO [31] studies. The debilitating nature of airway obstruction may have led to the cessation of work in those patients [30, 31]. Unlike many studies that reported an association between cigarette smoking and airway obstruction, this study did not find any association between current smoking and abnormal spirometry, possibly due to the low proportion of smokers in all our study participants (3.4%), but higher in HIV patient groups. Analysis of Demographic Health Surveys of 25 countries in Africa indicated that HIV patients with low socioeconomic status, that is the poor, less educated and manual workers, are more likely to smoke [32]. The prevalence of smoking in this study is similar to that of the general Ghanaian population (4.85%) as reported by the 2014 Ghana Demographic Health Survey [33]. On the other hand, previous smoking was associated with abnormal spirometric patterns and this is consistent with other studies [10, 26]. The findings of this study indicate that biomass exposure was associated with abnormal spirometry consistent with other reported studies [7, 15]. Biomass fumes contain about 50 air pollutants, with significant exposure similar to regular cigarette smoking [34]. These pollutants can irritate the respiratory tract by causing inflammation and tissue injury such as the excessive release of matrix metalloproteinases and increased hyaluronidase activity [35] that damage the lungs. In

this study, no association was found between abnormal spirometry and cART regimen and CD4 cell count, similar to what was reported in other HIV studies [7, 10, 15].

Respiratory symptoms

The prevalence of respiratory symptoms in our HIV population is similar to what has been reported in other sub-Saharan African populations. In urban Ugandan HIV patients, Ddungu *et al* reported the prevalence of any respiratory symptom to be 45%, with 30% reporting chronic cough, 21% reporting frequent phlegm production and wheezing and 26% reporting dyspnoea [16]. Similarly, in Cameroonian HIV patients, Pefura-Yone *et al* reported the prevalence of any respiratory symptom to be 47.5% while that of cough and dyspnoea were 25.5% and 36.4% respectively [17]. Contrary to the finding of our study, the prevalence of respiratory symptoms reported in Nigerian HIV patients was low (17%) [15] and HIV patients in South Africa had <4% of any respiratory symptoms [18]. Systematic review and meta-analysis of studies conducted in developed countries have similarly reported a high prevalence of respiratory symptoms irrespective of access to cART and high viremic control [9]. In this study, HIV status and medium-high biomass exposure were associated with respiratory symptoms in the adjusted model. Respiratory symptoms are known to be common with increasing age due to prolonged exposure to environmental toxins and decreased physiological capacity [12]. The participants in this study had a low smoking burden but high biomass exposure and environmental pollution common in the peri-urban area might be responsible for the high prevalence of respiratory symptoms [34]. With the popularity of spirometry for screening airway obstruction, respiratory symptoms are being given less attention. However, the symptoms experienced by HIV patients indicates functional status and quality of life, even if they correlate poorly with spirometry [9].

Limitations of the study

This study was conducted in a single primary healthcare facility in peri-urban Ghana, and hence, the findings may differ from the urban and rural populations, as well as other healthcare facilities in a different stratum. An inference of causality cannot be made from the findings of the study due to the cross-sectional design. It may be possible that some of the study participants had abnormal spirometry before HIV infection. The use of questionnaires in data collection of chronic respiratory symptoms may be affected by recall bias, which may lead to the misclassification of some study participants. This study did not assess important parameters like physical activity level, cART adherence, previous AIDS events and viral suppression which are reported to have an impact on pulmonary health. In this study, airway obstruction was defined by single spirometry. It has been reported in patients diagnosed with COPD using spirometry, 20% reversed from OSP to normal spirometry after one year of diagnosis, and this increased to 28% after two years. Among patients diagnosed with normal spirometric patterns, 11% progressed to OSP after one year and this increased to 17% after two years [36]. Therefore, categorising patients with abnormal spirometric patterns as COPD using a single spirometric measurement, as done in this study, may lead to potential misclassification. In addition, the reference equations for the Cameroonian population were employed to compute prediction values and LLN for spirometric values, and this yielded different outcomes compared to the GLI reference equations for African-Americans. The GLI equation is reported to significantly underestimate FEV1 and FVC by 9% in the Cameroonian population [37], implying significant errors in studies conducted in SSA that applied the GLI reference equation.

Conclusion

In conclusion, this study has shown that in PLWH without any recent pulmonary infection in a peri-urban area of Ghana, the prevalence of OSP was higher among cART-treated and cART-naïve HIV patients groups compared non-HIV control. The prevalence of RSP was similar among various study groups. This study has also shed light on the various factors

associated with spirometric abnormalities with the hope that future studies may investigate the possibility of using interventions targeted at these factors to reduce pulmonary dysfunction in HIV patients.

Supporting information

Table S1. Factors associated with obstructive lung pattern in cART-treated HIV patients
Table S2. Factors associated with obstructive lung pattern in cART-naïve HIV patients
Table S3. Factors associated with obstructive lung pattern in non-HIV controls
Table S4. Factors associated with restrictive lung pattern in cART-treated HIV patients
Table S5. Factors associated with restrictive lung pattern in cART-naïve HIV patients
Table S6. Factors associated with restrictive lung pattern in non-HIV controls
Table S7. Respiratory symptoms and spirometric abnormalities among cART-treated HIV patients

Acknowledgements: Our sincere thanks go to the staff and patients of Atua Government Hospital who assisted in data collection, especially Mr Samuel Essel and Mrs Nneka Essel.

Funding: There was no funding for this study.

Availability of data and materials: The dataset supporting the conclusion of this study is available at the OSF platform with the link: https://osf.io/bg7r2/?view_only=f349189f097c4f00bf46c13933760a30

Contributions:

KY contributed to the conception, design, data analysis, and drafting of the manuscript and bears the primary responsibility for the content of the manuscript.

LM collected the data and revised the manuscript.

KBA was involved in the supervision and revision of the manuscript.

All the authors read and approved the content of the manuscript.

Consent for publication: Not applicable

Competing interests: The authors declare that they have no competing interests.

Reference

1. James SL, Abate D, Abate KH, Abay SM, Abbafati C, Abbasi N, Abbastabar H, Abd-Allah F, Abdela J, Abdelalim A: **Global, regional, and national incidence, prevalence, and years lived with disability for 354 diseases and injuries for 195 countries and territories, 1990–2017: a systematic analysis for the Global Burden of Disease Study 2017**. *The Lancet* 2018, **392**(10159):1789-1858.
2. Roth GA, Abate D, Abate KH, Abay SM, Abbafati C, Abbasi N, Abbastabar H, Abd-Allah F, Abdela J, Abdelalim A: **Global, regional, and national age-sex-specific mortality for 282 causes of death in 195 countries and territories, 1980–2017: a systematic analysis for the Global Burden of Disease Study 2017**. *The Lancet* 2018, **392**(10159):1736-1788.
3. Ghana AIDS Commission: **National and Sub-National HIV and AIDS Estimates and Projections 2020 Report**. In., vol. 2020. Accra: Ghana AIDS Commision; 2021.
4. Teeraananchai S, Kerr S, Amin J, Ruxrungtham K, Law M: **Life expectancy of HIV-positive people after starting combination antiretroviral therapy: a meta-analysis**. *HIV medicine* 2017, **18**(4):256-266.
5. Yang H-Y, Beymer MR, Suen S-c: **Chronic Disease Onset Among People Living with HIV and AIDS in a Large Private Insurance Claims Dataset**. *Scientific Reports* 2019, **9**(1):18514.
6. Adeloye D, Song P, Zhu Y, Campbell H, Sheikh A, Rudan I: **Global, regional, and national prevalence of, and risk factors for, chronic obstructive pulmonary disease (COPD) in 2019: a systematic review and modelling analysis**. *The Lancet Respiratory Medicine* 2022, **10**(5):447-458.
7. Kayongo A, Wosu AC, Naz T, Nassali F, Kalyesubula R, Kirenga B, Wise RA, Siddharthan T, Checkley W: **Chronic Obstructive Pulmonary Disease Prevalence and Associated Factors in a Setting of Well-Controlled HIV, A Cross-Sectional Study**. *COPD: Journal of Chronic Obstructive Pulmonary Disease* 2020, **17**(3):297-305.
8. Bigna JJ, Kenne AM, Asangbeh SL, Sibetcheu AT: **Prevalence of chronic obstructive pulmonary disease in the global population with HIV: a systematic review and meta-analysis**. *The Lancet Global Health* 2018, **6**(2):e193-e202.
9. Brown J, Roy A, Harris R, Filson S, Johnson M, Abubakar I, Lipman M: **Respiratory symptoms in people living with HIV and the effect of antiretroviral therapy: a systematic review and meta-analysis**. *Thorax* 2017, **72**(4):355-366.
10. Drummond MB, Huang L, Diaz PT, Kirk GD, Kleerup EC, Morris A, Rom W, Weiden MD, Zhao E, Thompson B *et al*: **Factors associated with abnormal spirometry among HIV-infected individuals**. *Aids* 2015, **29**(13):1691-1700.
11. Gupte AN, Wong ML, Msandiwa R, Barnes GL, Golub J, Chaisson RE, Hoffmann CJ, Martinson NA: **Factors associated with pulmonary impairment in HIV-infected South African adults**. *PLOS ONE* 2017, **12**(9):e0184530.
12. Vaz Fragoso CA, Gill TM: **Respiratory Impairment and the Aging Lung: A Novel Paradigm for Assessing Pulmonary Function**. *The Journals of Gerontology: Series A* 2011, **67A**(3):264-275.
13. Venkateshiah SB, Ioachimescu OC, McCarthy K, Stoller JK: **The Utility of Spirometry in Diagnosing Pulmonary Restriction**. *Lung* 2008, **186**(1):19-25.
14. Guerra S, Sherrill DL, Venker C, Halonen M, Martinez FD: **Chronic Restrictive Lung Pattern Is Associated with Increased Mortality Risk**. In: *American Thoracic Society 2009 International Conference.* vol. C34. COPD PHENOTYPES. San Diego Convention centre: American Journal of Respiratory and Critical Care Medicine; 2009: A4325.
15. Akanbi MO, Taiwo BO, Achenbach CJ, Ozoh OB, Obaseki DO, Sule H, Agbaji OO, Ukoli CO: **HIV Associated Chronic Obstructive Pulmonary Disease in Nigeria**. *J AIDS Clin Res* 2015, **6**(5).
16. Ddungu A, Semitala FC, Castelnuovo B, Sekaggya-Wiltshire C, Worodria W, Kirenga BJ: **Chronic obstructive pulmonary disease prevalence and associated factors in an urban HIV clinic in a low income country**. *PLOS ONE* 2021, **16**(8):e0256121.
17. Pefura-Yone EW, Fodjeu G, kengne AP, Roche N, Kuaban C: **Prevalence and determinants of chronic obstructive pulmonary disease in HIV infected patients in an African country with low level of tobacco smoking**. *Respiratory Medicine* 2015, **109**(2):247-254.

18. van Riel SE, Klipstein-Grobusch K, Barth RE, Grobbee DE, Feldman C, Shaddock E, Stacey SL, Venter WDF, Vos AG: **Predictors of impaired pulmonary function in people living with HIV in an urban African setting**. *South Afr J HIV Med* 2021, **22**(1):1252.
19. Sidhu MK, Ravindra K, Mor S, John S: **Household air pollution from various types of rural kitchens and its exposure assessment**. *Science of The Total Environment* 2017, **586**:419-429.
20. Ferris BG: **Epidemiology Standardization Project (American Thoracic Society)**. *Am Rev Respir Dis* 1978, **118**(6 Pt 2):1-120.
21. Graham BL, Steenbruggen I, Miller MR, Barjaktarevic IZ, Cooper BG, Hall GL, Hallstrand TS, Kaminsky DA, McCarthy K, McCormack MC *et al*: **Standardization of Spirometry 2019 Update. An Official American Thoracic Society and European Respiratory Society Technical Statement**. *American Journal of Respiratory and Critical Care Medicine* 2019, **200**(8):e70-e88.
22. Pefura-Yone EW, Balkissou AD, Poka-Mayap V, Djenabou A, Massongo M, Ofimboudem NA, Mayoh-Nguemfo CF, Tsala AG, Hadjara H, Amougou F: **Spirometric reference equations for Cameroonians aged 4 to 89 years derived using lambda, mu, sigma (LMS) method**. *BMC Pulmonary Medicine* 2021, **21**(1):344.
23. van Gemert F, Kirenga B, Chavannes N, Kamya M, Luzige S, Musinguzi P, Turyagaruka J, Jones R, Tsiligianni I, Williams S *et al*: **Prevalence of chronic obstructive pulmonary disease and associated risk factors in Uganda (FRESH AIR Uganda): a prospective cross-sectional observational study**. *Lancet Glob Health* 2015, **3**(1):e44-51.
24. Vaz Fragoso CA, Van Ness PH, Murphy TE, McAvay GJ: **Spirometric impairments, cardiovascular outcomes, and noncardiovascular death in older persons**. *Respiratory Medicine* 2018, **137**:40-47.
25. Siddharthan T, Grigsby M, Miele CH, Bernabe-Ortiz A, Miranda JJ, Gilman RH, Wise RA, Porter JC, Hurst JR, Checkley W: **Prevalence and risk factors of restrictive spirometry in a cohort of Peruvian adults**. *Int J Tuberc Lung Dis* 2017, **21**(9):1062-1068.
26. Schultz K, D'Aquino LC, Soares MR, Gimenez A, Pereira CAdC: **Lung volumes and airway resistance in patients with a possible restrictive pattern on spirometry**. *Jornal Brasileiro de Pneumologia* 2016, **42**:341-347.
27. Hyatt RE, Cowl CT, Bjoraker JA, Scanlon PD: **Conditions Associated With an Abnormal Nonspecific Pattern of Pulmonary Function Tests**. *CHEST* 2009, **135**(2):419-424.
28. Aryal S, Diaz-Guzman E, Mannino DM: **COPD and gender differences: an update**. *Translational Research* 2013, **162**(4):208-218.
29. Camp PG, Goring SM: **Gender and the Diagnosis, Management, and Surveillance of Chronic Obstructive Pulmonary Disease**. *Proceedings of the American Thoracic Society* 2007, **4**(8):686-691.
30. Grønseth R, Erdal M, Tan WC, Obaseki DO, Amaral Andre FS, Gislason T, Juvekar SK, Koul PA, Studnicka M, Salvi S *et al*: **Unemployment in chronic airflow obstruction around the world: results from the BOLD study**. *European Respiratory Journal* 2017, **50**(3):1700499.
31. Montes de Oca M, Halbert RJ, Talamo C, Perez-Padilla R, Lopez MV, Muiño A, Jardim JRB, Valdivia G, Pertuzé J, Moreno D *et al*: **Paid employment in subjects with and without chronic obstructive pulmonary disease in five Latin American cities: the PLATINO study**. *The International Journal of Tuberculosis and Lung Disease* 2011, **15**(9):1259-1264.
32. Murphy JD, Liu B, Parascandola M: **Smoking and HIV in Sub-Saharan Africa: A 25-Country Analysis of the Demographic Health Surveys**. *Nicotine & Tobacco Research* 2018, **21**(8):1093-1102.
33. Nketiah-Amponsah E, Afful-Mensah G, Ampaw S: **Determinants of cigarette smoking and smoking intensity among adult males in Ghana**. *BMC Public Health* 2018, **18**(1):941.
34. Torres-Duque C, Maldonado D, Pérez-Padilla R, Ezzati M, Viegi G: **Biomass Fuels and Respiratory Diseases**. *Proceedings of the American Thoracic Society* 2008, **5**(5):577-590.
35. Silva R, Oyarzún M, Olloquequi J: **Pathogenic Mechanisms in Chronic Obstructive Pulmonary Disease Due to Biomass Smoke Exposure**. *Archivos de Bronconeumología (English Edition)* 2015, **51**(6):285-292.
36. Schermer TR, Robberts B, Crockett AJ, Thoonen BP, Lucas A, Grootens J, Smeele IJ, Thamrin C, Reddel HK: **Should the diagnosis of COPD be based on a single spirometry test?** *npj Primary Care Respiratory Medicine* 2016, **26**(1):16059.
37. Pefura-Yone EW, Balkissou AD, Poka-Mayap V, Djenabou A, Massongo M, Ofimboudem NA, Mayoh-Nguemfo CF, Tsala AG, Hadjara H, Amougou F: **Spirometric reference equations for Cameroonians aged 4 to 89 years derived using lambda, mu, sigma (LMS) method**. *BMC Pulm Med* 2021, **21**(1):344.

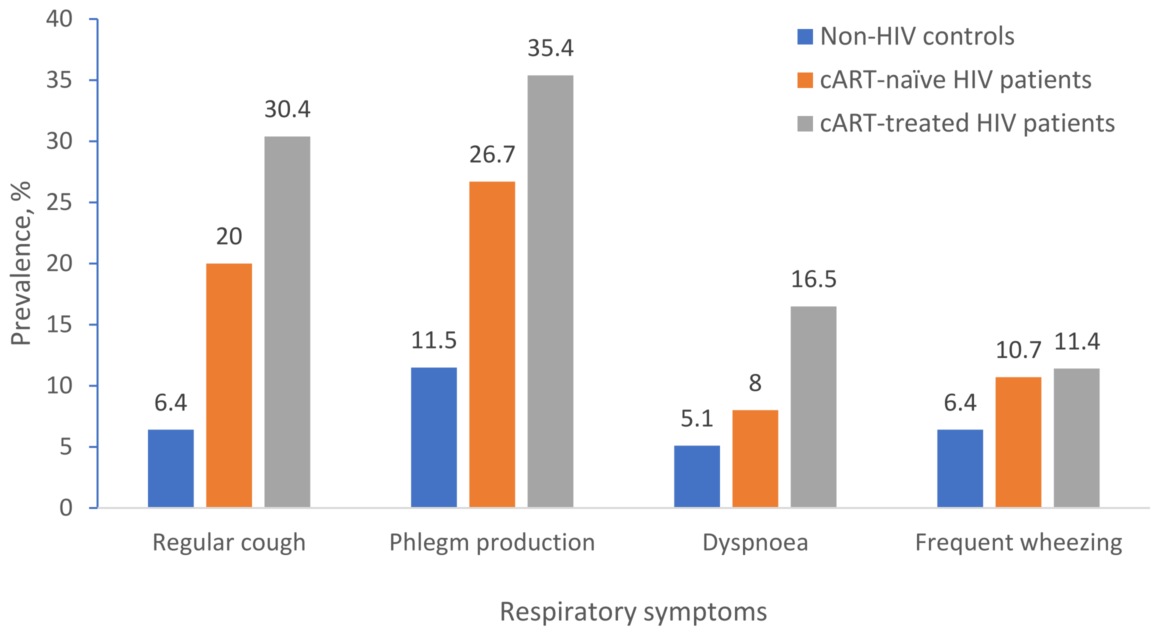

Fig 1. Prevalence of respiratory symptoms in study participants by the HIV status

HIV status was associated with regular cough (χ^2=29.5, p<0.001), phlegm production (χ^2=27.4, p<0.001) and dyspnoea (χ^2=12.2, p=0.002), but no association with frequent wheeze (χ^2=4.8, p=0.091).

 Click here to access/download;Figure;Figure 2 .docx

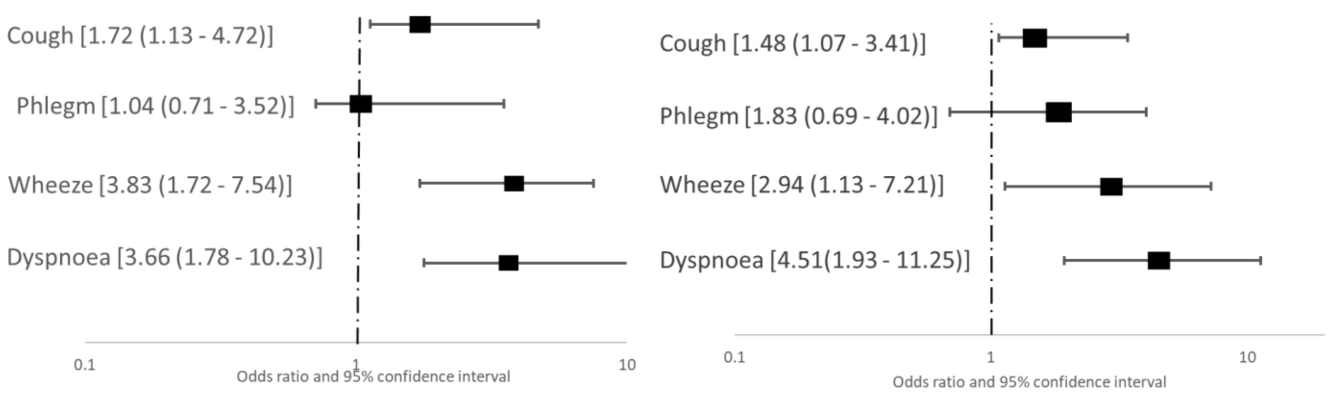

Fig. 2 Association between respiratory symptoms and obstructive spirometric pattern from unadjusted (left) and adjusted (right) logistic regression models.

Click here to access/download;Figure;Figure 3 .docx

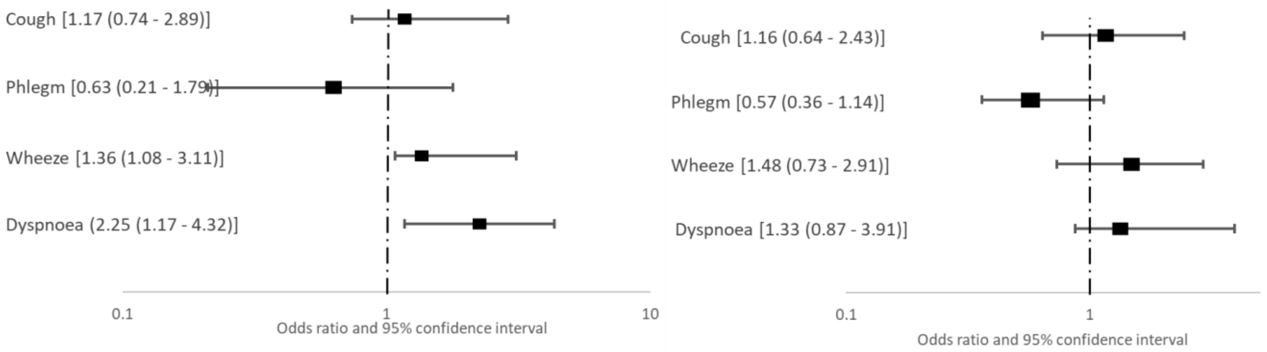

Fig. 3 Association between respiratory symptoms and restrictive spirometric pattern from unadjusted (left) and adjusted (right) logistic regression models.

Click here to access/download
Supporting Information
Supplementary Digital Content.docx

Click here to access/download
Other
jpg2pdf.pdf

Abnormal spirometric patterns and respiratory symptoms in HIV patients with no recent pulmonary infection in a periurban hospital in Ghana

Kwame Yeboah[1], Latif Musa[1,2], Kweku Bedu-Addo[2]

1. Department of Physiology, University of Ghana Medical School, Accra, Ghana
2. Department of Physiology, School of Medicine and Dentistry, KNUST, Kumasi, Ghana

Corresponding author: Dr Kwame Yeboah, Department of Physiology, University of Ghana Medical School, P O Box 4236, Accra, Ghana.

Email: kyeboah@ug.edu.gh/melvinky@gmail.com.

Short title: Spirometric patterns and respiratory symptoms in Ghanaian HIV patients

Abbreviations: SSA, sub-Saharan Africa; HIV, human immunodeficiency virus; PLWH, people living with HIV; cART, combination antiretroviral therapy; FEV1, forced expiratory volume in 1s; FVC, forced vital capacity; LLN, lower limit of normal; OSP, obstructive spirometric pattern; RSP, restrictive spirometric patterns; GLI, Global Lung Initiative.

Keywords: Abnormal spirometric patterns, Respiratory symptoms, HIV, cART, Ghana

Abstract

Background: Human immunodeficiency virus (HIV) infection is associated with chronic airway obstruction, even in patients who have achieved viral suppression from combination antiretroviral treatment (cART). Spirometry is a supplementary test that aids in diagnosing pulmonary dysfunction in people living with HIV.

Aim: To compare the prevalence of spirometric abnormalities among cART-treated HIV patients and cART-naïve HIV patients with non-HIV controls with no recent history of pulmonary infection in a peri-urban hospital in Ghana.

Methods: In a case-control design, spirometry was performed in 158 cART-treated HIV patients, 150 cART-naïve HIV patients and 156 non-HIV controls. Clinical, sociodemographic data and respiratory symptoms were collected using a structured questionnaire. Spirometric abnormalities were categorised as obstructive (OSP) or restrictive (RSP) spirometric patterns based on the Cameroonian reference equation.

Results: The prevalence of OSP was higher in the cART-treated and cART-naïve HIV patients compared to non-HIV controls (13.9% vs 10.7% vs 5.1% respectively, p = 0.026), whereas that of RSP was similar among the study groups. Respiratory symptoms were common among cART-treated and cART-naïve HIV patients compared to non-HIV controls (48.1% vs 40% vs 19.2% respectively, p<0.001). The major factors associated with OSP were female gender [OR (95% CI)=2.46 (1.09 – 5.13), p=0.031], former cigarette smoking [1.92 (1.04 – 3.89), p<0.001], exposure to medium-to-high levels of biomass [3.07 (1.16 – 8.73), p=0.019], presence of a respiratory symptom [1.89 (1.11 – 5.08), p=0.029] and unemployment [3.26 (1.19 – 8.95), p=0.042].~~, and that~~The major determinants of RSP were age, female gender [1.74 (1.05 – 4.29), p=0.041], former cigarette smoking [2.31 (1.27 – 6.77), p<0.001] and~~,~~ medium-to-high biomass exposure [1.58 (1.06 – 5.37), p=0.043].

Conclusion: In HIV patients without any recent pulmonary infection in a peri-urban area of Ghana, there was a higher prevalence of OSP among cART-treated and cART naïve HIV patients compared to the non-HIV control. However, the prevalence of RSP was similar among various study groups.

Introduction

Sub-Saharan Africa (SSA) has the largest population of people living with human immunodeficiency virus (HIV); this is associated with healthcare, socio-economic and developmental challenges [1]. It was reported in 2017 that 71% of the global total number of people living with HIV (PLWH) resides in SSA, with 75% of deaths and 65% of new HIV infections occurring in the region [2]. In Ghana, the prevalence of HIV infection is 1.7% and is concentrated mostly in urban areas [3]. With the introduction of effective combination antiretroviral therapy (cART), PLWH now lives longer due to decreased morbidity and mortality from opportunistic infections [4]. This has increased the susceptibility of PLWH to chronic diseases at greater rates than those observed in HIV-uninfected persons, making HIV infection a manageable chronic disease [5]. Chronic obstructive pulmonary disease (COPD) is one of the major causes of death and disability [2] with an estimated global prevalence of 10.3% [6]. HIV infection has been associated with an increased prevalence of COPD, with some studies suggesting that the prevalence of COPD is 21% higher in PLWH compared to the general population [7]. The high burden of COPD persists in PLWH even after adjustments for risk factors such as smoking, opportunistic infections and injection drug use [4, 8-11].

Spirometry is a simple, effective and reproducible method that can aid the diagnosis of COPD, and the results can be categorized into two major patterns of abnormalities. An obstructive spirometric pattern (OSP) is characterized by a disproportional reduction in the forced expiratory volume in 1 second (FEV1) relative to the forced vital capacity (FVC), leading to a reduction in the FEV1/FVC ratio to less than the lower limit of normal (LLN) of a similar population [12, 13]. A restrictive spirometric pattern (RSP) is defined by a normal FEV1/FVC (>LLN), but an FVC is less than 80% of the predicted value. Both OSP and RSP are associated with increased mortality in the general population [13, 14]. Despite the high prevalence of PLWH in SSA, few studies have reported the prevalence and risk factors for these abnormal

spirometric patterns with recent reports from the FRESH AIR study in Uganda [5, 7, 8, 15-18]. Most of these studies attributed abnormal spirometry in HIV to opportunistic pulmonary infection. This study compared the prevalence of spirometric abnormalities among HIV patients on cART treatment and cART-naïve HIV patients with non-HIV controls with no recent history of pulmonary infection in a peri-urban hospital in Ghana. The study hypothesized that HIV infection would increase the prevalence of spirometric abnormalities compared to those with no HIV infection. Also, the study investigated various factors associated with spirometric abnormalities in PLWH in Ghana.

Methods

Study participants and design

The study used a case-control design, conducted from October 2019 through February 2020, with HIV patients as cases and the controls were non-HIV individuals who visited the facility for voluntary testing of their HIV status. HIV patients were categorized as those on cART management and cART-naïve HIV patients who have not been on cART treatment for more than 4 weeks. The study was conducted at Atua Government Hospital, a 150-bed primary healthcare facility, located in the Lower Manya Krobo Municipality ~~Agormanya, a peri-urban town~~ in the Eastern region of Ghana. The Lower Manya Krobo Municipality ~~Agormanya area~~ has a higher prevalence of HIV infection (7%) compared to the national prevalence which stands at 1.7% in 2020 [3]. The hospital has about 3000 HIV patients on its register. Participants younger than 20 years, those diagnosed with severe heart diseases or hospitalized for cardiopulmonary complications within the past year, those with a diagnosis of lung infection or disease from their clinical case notes or answering positively to the question: “Have you been told by your health worker that you have lung disease?” were excluded from the study. In

addition, pregnant women, any individual with mental illness, active or recent (within the past 24 months) tuberculosis or lung infection and those with other conditions contraindicated for spirometry were excluded from the study. Initially, 180 cART-treated HIV patients, 170 cART-naïve controls and 170 non-HIV controls were recruited into the study. However, 22 cART-treated HIV patients, 20 cART-naïve HIV patients and 12 non-HIV controls failed to produce the appropriate manoeuvres required for accurate spirometry recordings, and hence, they were excluded from the analysis. Ethical approval was granted by the Korle Bu Teaching Hospital Institutional Review Board (Protocol ID number: KBTH-IRB/000131-2018) and all participants provided voluntary informed consent before joining the study.

Data collection

A structured questionnaire was used to obtain data on sociodemographic factors like age, gender, lifestyle factors (smoking, alcohol intake), medical history, current HIV medication, occupation, education, marital status and biomass exposure. Medium-high biomass exposure was defined as those who reported using firewood, sawdust, cow dung or corn cubs as a fuel source for work or cooking at home on most days [19]. Respiratory symptoms were assessed using a modified version of the American Thoracic Society respiratory questionnaire, which included the following questions: “Do you usually have coughs without cold for more than 2 months in a year?”, “Do you usually bring up phlegm from your chest when you don’t have a cold?”, “Do you have difficulty breathing when you walk fast, use a staircase or run?” and “Have you ever had an attack of wheezing or whistling in your chest that made you feel short of breath?” [20]. These questions were administered in the local dialect for participants with limited formal education.

Body weight and height were measured using a stadiometer in light clothing with footwear removed, with body mass index (BMI) calculated as weight/height^2 and categorized as

underweight (< 18.5 kg/m^2), normal weight (18.5–24.9 kg/m^2), overweight (25–29.9 kg/m^2), and obese (≥ 30 kg/m^2). Hypertension was defined as self-reported use of antihypertensive treatment and/or systolic blood pressure ≥ 140 mm Hg and/or diastolic blood pressure ≥ 90 mm Hg. Venous blood samples were collected from HIV patients to measure CD4+ lymphocytes.

Spirometry was performed by a qualified technician using a Vitalograph Pneumotrac desktop spirometer model 6800 following the ATS/ERS 2019 standard guidelines [21]. Abnormal spirometric patterns were computed based on fixed ratio and lower limits of normal (LLN) using the Cameroonian spirometric equation [22]. The normal spirometric pattern was defined by FEV1, FVC, and FEV1/FVC all > LLN. Obstructive spirometric pattern (OSP) was defined as FEV1/FVC<0.7 with a percentage predicted FVC>80% using the fixed ratio or FEV1/FVC <LLN with FVC > LLN using the LLN method. A restrictive spirometric pattern was defined as FEV1/FVC>0.7 with a percentage predicted FVC<80% by fixed ratio or FVC < LLN with FEV1/FVC > LLN [22].

Sample size

The sample size required was calculated using two studies conducted in rural Uganda that reported the prevalence of OSP in HIV patients to be 6.2% [7] and that of the non-HIV population to be 16.2% [23]. Therefore, a minimum of 153 participants would be required to achieve a power of 80% at a 95% confidence level with an estimated odds ratio of 2.93.

Data analysis

Data were analysed using IBM SPSS version 28 software. Continuous descriptive data were presented as means and standard deviations for normally distributed data and comparisons between groups were made using one-way ANOVA, while non-normally distributed data were presented as medians and interquartile ranges and comparisons were made using the Kruskal-

Wallis' test. Categorical descriptive data were presented as frequencies and percentages and analysed using the chi-square test. Univariate and multivariable logistic regression models were used to analyse the odds of participants with various socio-demographic and clinical factors having abnormal spirometry and respiratory symptoms. A p-value < 0.05 was considered statistically significant.

Results

General characteristics of study participants

The age range of participants was 20 – 70 years with a mean age of 38.4±13.7 years. There was no difference in mean age among various categories of participants. There was a high proportion of females (67.2%) in this study participants and participants who currently (5.2%) or formerly (15.6%) smoked were mostly HIV patients. HIV patients were ~~likely to be~~mostly unemployed (11%) or self-employed (43.5%) and had no formal education (14.9%) or attended school up to junior high level (59.7%). HIV patients were mostly underweight (9.1%) and had greater biomass exposure from work or home (31.8%) ~~and were more likely to be underweight~~. Measurements from spirometry indicated that cART-treated HIV patients had reduced FEV_1, FVC and FEF_{25-75} l/min compared to cART-naïve HIV patients and non-HIV participants. Compared to the non-HIV controls, the prevalence of OSP was high in cART-naïve and cART-treated HIV patients using the LLN. There was no difference in the prevalence of RSP using the LLN. When the fixed ratio was used to determine spirometric abnormalities, there was no difference in the prevalence of OSP and RSP among the study groups (Table 1). The average duration of HIV infection in cART-treated HIV patients was 7.6±4.6 years and the average duration of cART treatment was 7.2±4.5 years. For the cART medication regimen, 94 (59.5%) patients were treated

with TDF/3TC/NVP or EFV regimens, 52 (32.9%) patients were on AZT/3TC/NVP or EFV regimens and 12 (7.6%) patients were on LPV/r-based regimens.

Table 1. General characteristics of study participants

	All Participants (n=464)	Non-HIV participants (n=156)	cART-naïve HIV patients (n=150)	cART-treated HIV patients (n=158)	p
Age, years	38.4±13.7	36.7±14.4	38.2±11.6	39±11.4	0.109
Females, n (%)	312 (67.2)	106 (67.9)	84 (56)	122 (77.2)	0.02
Smoking, n (%)					0.029
Current	16 (3.4)	2 (1.3)	4 (2.7)	10 (6.3)	
Former	57 (15.9)	9 (5.8)	22 (14.6)	26 (16.5)	
Never	187 (80.6)	71 (92.9)	124 (82.7)	132 (83.5)	
Alcohol intake, n (%)	102 (22)	38 (24.4)	36 (24)	28 (17.7)	0.55
Married, n (%)	198 (42.7)	70 (44.9)	62 (41.3)	66 (41.8)	0.79
Education, n (%)					<0.001
None	50 (10.8)	4 (2.6)	14 (9.3)	32 (20.3)	
Up to JHS	208 (44.8)	24 (15.4)	80 (53.3)	104 (65.8)	
SHS to tertiary	68 (14.7)	24 (15.4)	24 (16)	20 (12.7)	
Tertiary	138 (29.7)	104 (66.7)	32 (21.3)	2 (1.3)	
Employment, n (%)					<0.001
Unemployed	56 (12.1)	22 (14.1)	20 (13.3)	14 (8.9)	
Self-employed	148 (31.9)	14 (9)	54 (36)	80 (50.6)	
Employed	260 (56)	120 (76.9)	76 (50.7)	64 (40.5)	
Biomass exposure, n (%)					<0.001
Low	342 (73.7)	132 (84.6)	96 (64)	114 (72.2)	
Medium-high	122 (26.3)	24 (15.4)	54 (36)	44 (27.8)	
Weight, kg	60±13.2	68±13.3	64.4±7.3	65.8±14	0.204
BMI, kg/m^2	24.8±5	25.4±4.7	23.7±4.4	25.3±5.7	0.061
BMI categories, n (%)					0.024
Underweight	34 (7.4)	6 (3.9)	16 (10.7)	12 (7.6)	
Normal	238 (51.5)	70 (45.5)	94 (62.7)	74 (46.8)	
Overweight	116 (25.1)	46 (29.9)	18 (12)	52 (32.9)	
Obese	74 (16)	32 (20.8)	22 (14.7)	20 (12.7)	
Current CD4 count, cells/mm^2	405 (273 – 562)		403 (253 – 583)	430 (327 – 534)	0.804
Spirometric measurements					
FEV1, l	2.2±0.6	2.4±0.6	2.3±0.7	1.9±0.9$^{*\#}$	<0.001
FVC, l	2.7±0.7	3±0.7	2.9±0.8	2.3±0.5$^{*\#}$	<0.001
FEF_{25-75}, l/min	2.3±0.9	2.5±0.9	2.4±1	1.9±0.8$^{*\#}$	<0.001
FEV1/FVC, %	80.3±6.9	81.3±5.7	80±7.4	79.4±7.3	0.129
Abnormal spirometry by LLN, n (%)					

Obstructive	46 (9.9)	8 (5.1)	16 (10.7)*	22 (13.9)*	0.026
Restrictive	24 (19.8)	26 (16.7)	20 (13.3)	36 (22.8)	0.087
Abnormal spirometry by a fixed ratio, n (%)					
Obstructive	26 (5.6)	4 (2.6)	8 (5.3)	14 (8.9)	0.052
Restrictive	92 (19.8)	28 (17.9)	26 (17.3)	38 (24.1)	0.258
Respiratory symptoms, n (%)	166 (35.8)	30 (19.2)	60 (40)	76 (48.1)	<0.001

cART, combination antiretroviral therapy; JHS, junior high school; SHS, senior high school; BMI, body mass index; FEV1, forced expiratory volume in 1 second; FVC, forced vital capacity.

*: p<0.05 compared to non-HIV
#: p< 0.05 compared to untreated HIV patients

Respiratory symptoms

Among the entire study participants, 35.8% reported the presence of at least one respiratory symptom, with a higher proportion in cART-treated and cART-naïve HIV patients compared to non-HIV participants (48.1% vs 40% vs 19.2 respectively, p<0.001). Phlegm production and regular cough were commonly reported in 26.4% and 19% of the entire study participants respectively, with a higher proportion in cART-treated and cART-naïve HIV patients compared to non-HIV participants (phlegm: 35.4% vs 26.7% vs 6.4%, p<0.001; regular coughs: 30.4% vs 20% vs 11.5%, p<0.001 respectively). Similar trends were observed in participants who reported having dyspnoea on exertion and frequent wheeze symptoms (Fig. 1). Increasing age, being unemployed, former cigarette smoking, exposure to a high-medium level of biomass, being HIV patients with or without cART treatment and having basic or no education was associated with increased odds of having respiratory symptoms in unadjusted regression models. In the adjusted regression models, exposure to medium-high levels of biomass, being a cART-treated HIV or cART-naïve HIV patient were associated with increased odds of having respiratory symptoms (Table 2).

[*Please insert Fig. 1 here*]~~Prevalence of respiratory symptoms in study participants by the HIV status~~

Formatted: Superscript
Formatted: Superscript
Formatted: Font: Italic
Formatted: Font: Italic
Formatted: Font: Italic

Table 2. Factors associated with respiratory symptoms from logistic regression analyses

		Unadjusted model		Adjusted model	
		OR (95% CI)	p	OR (95% CI)	p
Age (per 1 yr increase)		1.02 (1.01 – 1.03)	0.007	1 (0.98 – 1.02)	0.734
Females (reference = males)		1.32 (0.87 – 1.99)	0.189	1.14 (0.69 – 1.88)	0.623
Unmarried (reference = married)		1.34 (0.91 – 1.97)	0.14	1.18 (0.76 – 1.86)	0.462
Alcohol intake (reference = non-drinkers)		0.97 (0.62 – 1.54)	0.909	1 (0.59 – 1.68)	0.986
Smoking (reference: never smoked)					
	Current	1.84 (0.68 – 4.98)	0.233	1.52 (0.5 – 4.64)	0.463
	Former	2.75 (1.12 – 5.08)	<0.001	1.94 (1 – 4.65)	0.071
Medium/high biomass exposure (reference = low biomass exposure)		2.72 (1.12 – 4.57)	0.008	1.87 (1.07 – 3.49)	0.037
HIV status (reference = non-HIV)					
	cART-naïve	2.8 (1.67 – 4.69)	<0.001	2.03 (1.1 – 3.74)	0.023
	cART-treated	3.89 (2.35 – 6.46)	<0.001	2.61 (1.32 – 5.17)	0.006
Employment (reference = Employed)					
	Self-employed	1.4 (0.92 – 2.13)	0.118	0.83 (0.5 – 1.36)	0.451
	Unemployed	1.88 (1.05 – 3.38)	0.035	1.78 (0.91 – 3.48)	0.091
Education (reference = tertiary)					
	None	3.32 (1.67 – 6.6)	<0.001	2.22 (0.89 – 5.51)	0.086
	Basic/JHS	3.21 (1.97 – 5.22)	<0.001	1.79 (0.89 – 3.62)	0.102
	SHS/Tech	0.93 (0.46 – 1.91)	0.85	0.62 (0.27 – 1.43)	0.263
BMI categories (reference: normal)					
	Underweight	1.85 (0.9 – 3.81)	0.095	1.58 (0.73 – 3.42)	0.25
	Overweight	0.68 (0.42 – 1.1)	0.116	0.6 (0.35 – 1.03)	0.062
	Obese	0.79 (0.45 – 1.37)	0.789	0.82 (0.44 – 1.51)	0.517

cART, combination antiretroviral therapy; JHS, junior high school; SHS, senior high school; BMI, body mass index.

Factors associated with abnormal spirometric patterns

In logistic regression analyses, being female, unmarried, a former smoker, unemployed, having no formal education, underweight and having respiratory symptoms were associated with increased odds of having OSP in the unadjusted models. In the adjusted models, female gender,

former cigarette smoking, medium-to-high biomass exposure, presence of respiratory symptoms, unemployed and being underweight were associated with increased odds of having OSP (Table 3). The associated factors of OSP among various categories of participants based on HIV status are shown in the supplementary attachment (Table S1 – S3).

Table 3. Factors associated with an obstructive spirometric pattern from logistic regression analyses

	Unadjusted model		Adjusted model	
	OR (95% CI)	p	OR (95% CI)	p
Age (per 1 yr increase)	0.79 (0.57 – 1.07)	0.745	0.91 (0.73 – 1.41)	0.576
Females (reference = males)	3.18 (1.24 – 8.85)	0.005	2.46 (1.09 – 5.13)	0.031
Unmarried (reference = married)	2.81 (1.35 – 5.83)	0.006	2.31 (0.86 – 5.19)	0.125
Alcohol intake (reference = non-drinkers)	0.53 (0.22 – 1.3)	0.166	0.69 (0.19 – 1.489)	0.631
Smoking (Reference: never smoked)				
Current	1.38 (0.3 – 6.28)	0.676	1.87 (0.36 – 9.75)	0.457
Former	2.67 (1.13 – 4.07)	<0.002	1.92 (1.04 – 3.89)	<0.001
Medium/high biomass exposure (reference = low biomass exposure)	1.49 (1.13 – 3.25)	0.004	3.07 (1.16 – 8.73)	0.019
Respiratory symptoms (reference = no respiratory symptom)	2.35 (1.26 – 4.4)	0.008	1.89 (1.11 – 5.08)	0.029
HIV status (reference: non-HIV)				
cART-naïve	2.24 (0.93 – 5.4)	0.073	1.29 (0.69 – 3.68)	0.548
cART-treated	3.08 (1.33 – 7.15)	0.009	1.79 (1.03 – 4.18)	0.037
Employment (reference = employed)				
Self-employed	0.51 (0.23 – 1.17)	0.112	0.94 (0.43 – 2.51)	0.481
Unemployed	2.46 (1.15 – 5.23)	0.02	3.26 (1.19 – 8.95)	0.042
Education (reference: tertiary)				
None	2.11 (1.08 – 6.71)	0.044	1.72 (0.79 – 6.04)	0.533
Basic/JHS	0.91 (0.43 – 1.9)	0.632	0.77 (0.32 – 1.26)	0.257
SHS/Tech	1.02 (0.36 – 2.84)	0.583	0.63 (0.41 – 1.73)	0.358
BMI categories (reference = normal)				
Underweight	3.11 (1.27 – 10.21)	<0.001	3.01 (1.12 – 10.16)	0.026
Overweight	1.09 (0.54 – 2.28)	0.691	0.98 (0.59 – 2.71)	0.864
Obese	1.12 (0.53 – 2.78)	0.748	1.06 (0.49 – 4.02)	0.877

cART, combination antiretroviral therapy; JHS, junior high school; SHS, senior high school; BMI, body mass index.

Concerning RSP, female gender, former smoking, medium-to-high biomass exposure and being a cART-treated HIV patient were associated with increased odds in unadjusted logistic regression models. In multivariable logistic regression models, age, female gender, being unmarried, previous cigarette smoking and medium-to-high biomass exposure were associated with increased odds of restrictive lung pattern (Table 4). The associated factors of RSP among various patient groups are shown in the supplementary attachment (Tables S4 – S6).

Table 4. Factors associated with a restrictive spirometric pattern from logistic regression analyses

	Unadjusted model		Adjusted model	
	OR (95% CI)	p	OR (95% CI)	p
Age (per 1 yr increase)	0.97 (0.93 – 1.01)	0.061	0.97 (0.65 – 1.29)	0.114
Females (reference = males)	1.67 (1.09 – 3.42)	0.013	1.74 (1.05 – 4.29)	0.041
Unmarried (reference = married)	0.75 (0.5 – 1.14)	0.18	1.31 (0.68 – 3.88)	0.741
Alcohol intake (reference = non-drinkers)	1.43 (0.88 – 2.310	0.151	1.68 (0.96 – 2.95)	0.069
Smoking (reference: never)				
Current	0.4 (0.09 – 1.78)	0.229	0.42 (0.09 – 2.95)	0.283
Former	3.07 (1.64 – 8.36)	<0.001	2.31 (1.27 – 6.77)	<0.001
Medium/high biomass exposure (reference = low biomass exposure)	2.15 (1.13 – 5.37)	<0.001	1.58 (1.06 – 5.37)	0.043
HIV status (reference= non-HIV controls)				
cART-naïve	0.89 (0.53 – 1.73)	0.888	1.67 (0.84 – 3.33)	0.144
cART-treated	1.75 (1.04 – 2.59)	0.016	4.89 (2.15 – 11.11)	<0.001
Employment (reference = employed)				
Self-employed	0.72 (0.44 – 1.17)	0.182	0.73 (0.23 – 1.79)	0.407
Unemployed	1.83 (1 – 3.34)	0.05	2.06 (0.92 – 4.14)	0.061
Education (reference= tertiary)				
None	1.89 (0.99 – 3.73)	0.067	2.12 (0.8 – 5.65)	0.131
Basic/JHS	0.99 (0.61 – 1.62)	0.979	0.97 (0.45 – 2.09)	0.94
SHS/Tech	0.49 (0.23 – 1.06)	0.069	0.43 (0.17 – 1.08)	0.071
BMI categories (reference = normal BMI)				
Underweight	0.84 (0.36 – 1.94)	0.678	1.31 (0.52 – 3.29)	0.562

	Overweight	0.95 (0.57 – 1.57)	0.837	0.65 (0.34 – 1.16)	0.148
	Obese	0.87 (0.48 – 1.6)	0.661	0.71 (0.36 – 1.14)	0.333

cART, combination antiretroviral therapy; JHS, junior high school; SHS, senior high school; BMI, body mass index.

Association between respiratory symptoms and abnormal spirometric patterns

In the entire study participants with OSP, 14 (31.8%) reported having regular cough, 16 (36.4%) reported regular phlegm production, and 12 (16.7%) reported frequent wheezing or dyspnoea on exertion. Among the participants with RSP, those reporting regular cough or frequent wheeze or dyspnoea on exertion were 24 (16.7) while 34 (23.6%) reported regular phlegm production. In logistic regression analyses, regular cough, frequent wheeze and dyspnoea on exertion were associated with increased odds of having OSP in crude and adjusted models (Fig. 2). Participants with RSP had increased odds of frequent wheeze and dyspnoea on exertion in unadjusted logistic regression analysis, but there was no association between respiratory symptoms and RSP in multivariable analyses (Fig. 3).

[Please insert Fig. 2 here]~~Association between respiratory symptoms and obstructive spirometric pattern from unadjusted (left) and adjusted (right) logistic regression models.~~

[Please insert Fig. 3 here]~~Association between respiratory symptoms and restrictive spirometric pattern from unadjusted (left) and adjusted (right) logistic regression models.~~

Discussion

Major findings

This is the first study to report the prevalence of abnormal spirometry in HIV patients in Ghana. The major findings of the study were: 1) The prevalence of OSP was higher among the higher in both cART-treated and cART-naïve HIV patient groups compared to non-HIV control~~HIV~~

patient groups compared to non-HIV controls, whereas the prevalence of RSP is similar among the study groups; 2) respiratory symptoms were common among HIV patients compared to non-HIV controls and were associated with abnormal spirometry; and 3) the major factors associated with OSP were female gender, former cigarette smoking, exposure to medium-to-high levels of biomass, presence of a respiratory symptom, unemployment and underweight and that of RSP were age, female gender, being unmarried, medium-to-high biomass exposure and being self-employed or unemployed.

Abnormal spirometric patterns.

The higher prevalence of OSP found in cART-treated HIV patients in our study is consistent with studies from other countries in sub-Saharan Africa. In the South African population, van Riel *et al* reported a higher prevalence of OSP in cART-treated HIV patients (34.2%) compared to cART-naïve HIV patients (11.2%). However, that study used a cut-off of 20%-LLN instead of the globally accepted 5%-LLN, which was used to characterize OSP in our study, and this may explain the usually higher prevalence of OSP reported in that particular study [18]. In contrast to the findings of the current study, Pefura-Yone *et al* reported lower prevalences of OSP, which was similar among the HIV patients and non-HIV controls using the 5%-LLN cut-off. [17]. Other cross-sectional studies in HIV patients in SSA have reported OSP prevalence between 3.1 – 15.4% [7, 11, 15-18], and a metanalysis reported the prevalence of OSP in HIV patients from 30 observational studies involving 151, 686 participants around the globe to be between 5.6% and 10.6% [8], similar to what was found in this study. Few studies have compared the prevalence of OSP in cART-treated HIV patients to that of cART-naïve HIV patients and non-HIV controls.

The findings of this study indicated that the prevalence of RSP was similar among cART-naïve HIV patients and non-HIV controls, but more common in cART-treated HIV patients. Most

spirometry studies conducted in HIV patients and other populations in SSA do not report the prevalence and associated factors of RSP, limiting our ability to compare the prevalence of RSP found in our study to other spirometry studies in SSA. The patterns of abnormal spirometry that were reported in this study differed from what was reported by Drummond *et al* reported in HIV patients in the United States. He found that, in a total of 37% abnormal spirometry, the majority of the patients had OSP (27% for OSP, compared to 10% for RSP) [10]. The prevalence of RSP observed in our non-HIV controls (16.7%) was similar to 7 – 14% reported in the general US population [10], but higher than 5.9% reported in the older population [24] and 4.7% reported in Peruvian adults [25], and far lower than to 36% reported by Schultz *et al* in Brazilians [26]. The variation in the prevalence of abnormal spirometric patterns may be due to different reference equations applied in various studies [26] and the fact that various insults may affect pulmonary health across various geographical locations.

The accuracy of the definition of RSP used in this and many other studies, FVC<80% predicted and FEV_1/FVC>LLN, in diagnosing RSP has been reported to be poor with low sensitivity but high negative predictive value to exclude patients without restrictive lung disease [27]. On the other hand, some studies reported that using FVC<80% predicted alone, without FEV_1/FVC>LLN, improves specificity and negative prediction of diagnosing restrictive lung disease [13]. These inconsistent definitions of RSP in literature and the failure of current guidelines to address this have resulted in the underappreciation of RSP values in spirometric studies. However, the utility and importance of RSP have been highlighted in some studies as a predictor of cardiovascular morbidity and mortality. For instance, the Cardiovascular Health Study reported that RSP is a significant predictor of CVD mortality and non-CVD mortality in older Caucasians [27]. Similarly, the TESOAD study reported that, although consistent RSP is found in 50% of patients with one-time spirometry, it is associated with an increased risk of all-cause mortality, and specifically, an increased risk of dying from diabetes and heart diseases

[14]. Therefore, there is a need for further investigation into the relevance of the high burden of RSP found in HIV patients and non-HIV controls in our study to characterize them properly to prevent future CVD events and deaths.

Factors associated with abnormal spirometry

The female gender was associated with an increased likelihood of having abnormal spirometry in this study. A systematic review has shown that females are more likely to report asthma and airway obstruction compared to men, but abnormal spirometry is similar in both genders [28, 29]. The association between female sex and abnormal spirometry found in our study may be due to the high representation of females in our study population (67.2%). This study found an association between unemployment and OSP, consistent with what has been reported in multicentre studies like BOLD [30] and PLATINO [31] studies. The debilitating nature of airway obstruction may have led to the cessation of work in those patients [30, 31]. Unlike many studies that reported an association between cigarette smoking and airway obstruction, this study did not find any association between current smoking and abnormal spirometry, possibly due to the low proportion of smokers in all our study participants (3.4%), but higher in HIV patient groups. Analysis of Demographic Health Surveys of 25 countries in Africa indicated that HIV patients with low socioeconomic status, that is the poor, less educated and manual workers, are more likely to smoke [32]. The prevalence of smoking in this study is similar to that of the general Ghanaian population (4.85%) as reported by the 2014 Ghana Demographic Health Survey [33]. On the other hand, previous smoking was associated with abnormal spirometric patterns and this is consistent with other studies [10, 26]. The findings of this study indicate that biomass exposure was associated with abnormal spirometry consistent with other reported studies [7, 15]. Biomass fumes contain about 50 air pollutants, with significant exposure similar to regular cigarette smoking [34]. These pollutants can irritate the respiratory tract by causing inflammation and tissue injury such as the excessive release of

matrix metalloproteinases and increased hyaluronidase activity [35] that damage the lungs. In this study, no association was found between abnormal spirometry and cART regimen and CD4 cell count, similar to what was reported in other HIV studies [7, 10, 15].

Respiratory symptoms

The prevalence of respiratory symptoms in our HIV population is similar to what has been reported in other sub-Saharan African populations. In urban Ugandan HIV patients, Ddungu *et al* reported the prevalence of any respiratory symptom to be 45%, with 30% reporting chronic cough, 21% reporting frequent phlegm production and wheezing and 26% reporting dyspnoea [16]. Similarly, in Cameroonian HIV patients, Pefura-Yone *et al* reported the prevalence of any respiratory symptom to be 47.5% while that of cough and dyspnoea were 25.5% and 36.4% respectively [17]. Contrary to the finding of our study, the prevalence of respiratory symptoms reported in Nigerian HIV patients was low (17%) [15] and HIV patients in South Africa had <4% of any respiratory symptoms [18]. Systematic review and meta-analysis of studies conducted in developed countries have similarly reported a high prevalence of respiratory symptoms irrespective of access to cART and high viremic control [9]. In this study, HIV status and medium-high biomass exposure were associated with respiratory symptoms in the adjusted model. Respiratory symptoms are known to be common with increasing age due to prolonged exposure to environmental toxins and decreased physiological capacity [12]. The participants in this study had a low smoking burden but high biomass exposure and environmental pollution common in the peri-urban area might be responsible for the high prevalence of respiratory symptoms [34]. With the popularity of spirometry for screening airway obstruction, respiratory symptoms are being given less attention. However, the symptoms experienced by HIV patients indicates functional status and quality of life, even if they correlate poorly with spirometry [9].

Limitations of the study

This study was conducted in a single primary healthcare facility in peri-urban Ghana, and hence, the findings may differ from the urban and rural populations, as well as other healthcare facilities in a different stratum. An inference of causality cannot be made from the findings of the study due to the cross-sectional design. It may be possible that some of the study participants had abnormal spirometry before HIV infection. The use of questionnaires in data collection of chronic respiratory symptoms may be affected by recall bias, which may lead to the misclassification of some study participants. This study did not assess important parameters like physical activity level, cART adherence, previous AIDS events and viral suppression which are reported to have an impact on pulmonary health. In this study, airway obstruction was defined by single spirometry. It has been reported in patients diagnosed with COPD using spirometry, 20% reversed from OSP to normal spirometry after one year of diagnosis, and this increased to 28% after two years. Among patients diagnosed with normal spirometric patterns, 11% progressed to OSP after one year and this increased to 17% after two years [36]. Therefore, categorising patients with abnormal spirometric patterns as COPD using a single spirometric measurement, as done in this study, may lead to potential misclassification. In addition, the reference equations for the Cameroonian population were employed to compute prediction values and LLN for spirometric values, and this yielded different outcomes compared to the GLI reference equations for African-Americans. The GLI equation is reported to significantly underestimate FEV1 and FVC by 9% in the Cameroonian population [37], implying significant errors in studies conducted in SSA that applied the GLI reference equation.

Conclusion

In conclusion, this study has shown that in PLWH without any recent pulmonary infection in a peri-urban area of Ghana, the prevalence of OSP was higher among cART-treated and cART-naïve HIV patients groups compared ~~to cART-naïve HIV patients and~~ non-HIV control. The prevalence of RSP was similar among various study groups. This study has also

shed light on the various factors associated with spirometric abnormalities with the hope that future studies may investigate the possibility of using interventions targeted at these factors to reduce pulmonary dysfunction in HIV patients.

Supporting information

Table S1. Factors associated with obstructive lung pattern in cART-treated HIV patients
Table S2. Factors associated with obstructive lung pattern in cART-naïve HIV patients
Table S3. Factors associated with obstructive lung pattern in non-HIV controls
Table S4. Factors associated with restrictive lung pattern in cART-treated HIV patients
Table S5. Factors associated with restrictive lung pattern in cART-naïve HIV patients
Table S6. Factors associated with restrictive lung pattern in non-HIV controls
Table S7. Respiratory symptoms and spirometric abnormalities among cART-treated HIV patients

Acknowledgements: Our sincere thanks go to the staff and patients of Atua Government Hospital who assisted in data collection, especially Mr Samuel Essel and Mrs Nneka Essel.

Funding: There was no funding for this study.

Availability of data and materials: The dataset supporting the conclusion of this study is available at the OSF platform with the link: https://osf.io/bg7r2/?view_only=f349189f097c4f00bf46c13933760a30

Contributions:

KY contributed to the conception, design, data analysis, and drafting of the manuscript and bears the primary responsibility for the content of the manuscript.

LM collected the data and revised the manuscript.

KBA was involved in the supervision and revision of the manuscript.

All the authors read and approved the content of the manuscript.

Consent for publication: Not applicable

Competing interests: The authors declare that they have no competing interests.

Reference

1. James SL, Abate D, Abate KH, Abay SM, Abbafati C, Abbasi N, Abbastabar H, Abd-Allah F, Abdela J, Abdelalim A: **Global, regional, and national incidence, prevalence, and years lived with disability for 354 diseases and injuries for 195 countries and territories, 1990–2017: a systematic analysis for the Global Burden of Disease Study 2017**. *The Lancet* 2018, **392**(10159):1789-1858.
2. Roth GA, Abate D, Abate KH, Abay SM, Abbafati C, Abbasi N, Abbastabar H, Abd-Allah F, Abdela J, Abdelalim A: **Global, regional, and national age-sex-specific mortality for 282 causes of death in 195 countries and territories, 1980–2017: a systematic analysis for the Global Burden of Disease Study 2017**. *The Lancet* 2018, **392**(10159):1736-1788.
3. Ghana AIDS Commission: **National and Sub-National HIV and AIDS Estimates and Projections 2020 Report**. In., vol. 2020. Accra: Ghana AIDS Commision; 2021.
4. Teeraananchai S, Kerr S, Amin J, Ruxrungtham K, Law M: **Life expectancy of HIV-positive people after starting combination antiretroviral therapy: a meta-analysis**. *HIV medicine* 2017, **18**(4):256-266.
5. Yang H-Y, Beymer MR, Suen S-c: **Chronic Disease Onset Among People Living with HIV and AIDS in a Large Private Insurance Claims Dataset**. *Scientific Reports* 2019, **9**(1):18514.
6. Adeloye D, Song P, Zhu Y, Campbell H, Sheikh A, Rudan I: **Global, regional, and national prevalence of, and risk factors for, chronic obstructive pulmonary disease (COPD) in 2019: a systematic review and modelling analysis**. *The Lancet Respiratory Medicine* 2022, **10**(5):447-458.
7. Kayongo A, Wosu AC, Naz T, Nassali F, Kalyesubula R, Kirenga B, Wise RA, Siddharthan T, Checkley W: **Chronic Obstructive Pulmonary Disease Prevalence and Associated Factors in a Setting of Well-Controlled HIV, A Cross-Sectional Study**. *COPD: Journal of Chronic Obstructive Pulmonary Disease* 2020, **17**(3):297-305.
8. Bigna JJ, Kenne AM, Asangbeh SL, Sibetcheu AT: **Prevalence of chronic obstructive pulmonary disease in the global population with HIV: a systematic review and meta-analysis**. *The Lancet Global Health* 2018, **6**(2):e193-e202.
9. Brown J, Roy A, Harris R, Filson S, Johnson M, Abubakar I, Lipman M: **Respiratory symptoms in people living with HIV and the effect of antiretroviral therapy: a systematic review and meta-analysis**. *Thorax* 2017, **72**(4):355-366.
10. Drummond MB, Huang L, Diaz PT, Kirk GD, Kleerup EC, Morris A, Rom W, Weiden MD, Zhao E, Thompson B *et al*: **Factors associated with abnormal spirometry among HIV-infected individuals**. *Aids* 2015, **29**(13):1691-1700.
11. Gupte AN, Wong ML, Msandiwa R, Barnes GL, Golub J, Chaisson RE, Hoffmann CJ, Martinson NA: **Factors associated with pulmonary impairment in HIV-infected South African adults**. *PLOS ONE* 2017, **12**(9):e0184530.
12. Vaz Fragoso CA, Gill TM: **Respiratory Impairment and the Aging Lung: A Novel Paradigm for Assessing Pulmonary Function**. *The Journals of Gerontology: Series A* 2011, **67A**(3):264-275.
13. Venkateshiah SB, Ioachimescu OC, McCarthy K, Stoller JK: **The Utility of Spirometry in Diagnosing Pulmonary Restriction**. *Lung* 2008, **186**(1):19-25.
14. Guerra S, Sherrill DL, Venker C, Halonen M, Martinez FD: **Chronic Restrictive Lung Pattern Is Associated with Increased Mortality Risk**. In: *American Thoracic Society 2009 International Conference.* vol. C34. COPD PHENOTYPES. San Diego Convention centre: American Journal of Respiratory and Critical Care Medicine; 2009: A4325.
15. Akanbi MO, Taiwo BO, Achenbach CJ, Ozoh OB, Obaseki DO, Sule H, Agbaji OO, Ukoli CO: **HIV Associated Chronic Obstructive Pulmonary Disease in Nigeria**. *J AIDS Clin Res* 2015, **6**(5).
16. Ddungu A, Semitala FC, Castelnuovo B, Sekaggya-Wiltshire C, Worodria W, Kirenga BJ: **Chronic obstructive pulmonary disease prevalence and associated factors in an urban HIV clinic in a low income country**. *PLOS ONE* 2021, **16**(8):e0256121.
17. Pefura-Yone EW, Fodjeu G, kengne AP, Roche N, Kuaban C: **Prevalence and determinants of chronic obstructive pulmonary disease in HIV infected patients in an African country with low level of tobacco smoking**. *Respiratory Medicine* 2015, **109**(2):247-254.

18. van Riel SE, Klipstein-Grobusch K, Barth RE, Grobbee DE, Feldman C, Shaddock E, Stacey SL, Venter WDF, Vos AG: **Predictors of impaired pulmonary function in people living with HIV in an urban African setting**. *South Afr J HIV Med* 2021, **22**(1):1252.
19. Sidhu MK, Ravindra K, Mor S, John S: **Household air pollution from various types of rural kitchens and its exposure assessment**. *Science of The Total Environment* 2017, **586**:419-429.
20. Ferris BG: **Epidemiology Standardization Project (American Thoracic Society)**. *Am Rev Respir Dis* 1978, **118**(6 Pt 2):1-120.
21. Graham BL, Steenbruggen I, Miller MR, Barjaktarevic IZ, Cooper BG, Hall GL, Hallstrand TS, Kaminsky DA, McCarthy K, McCormack MC *et al*: **Standardization of Spirometry 2019 Update. An Official American Thoracic Society and European Respiratory Society Technical Statement**. *American Journal of Respiratory and Critical Care Medicine* 2019, **200**(8):e70-e88.
22. Pefura-Yone EW, Balkissou AD, Poka-Mayap V, Djenabou A, Massongo M, Ofimboudem NA, Mayoh-Nguemfo CF, Tsala AG, Hadjara H, Amougou F: **Spirometric reference equations for Cameroonians aged 4 to 89 years derived using lambda, mu, sigma (LMS) method**. *BMC Pulmonary Medicine* 2021, **21**(1):344.
23. van Gemert F, Kirenga B, Chavannes N, Kamya M, Luzige S, Musinguzi P, Turyagaruka J, Jones R, Tsiligianni I, Williams S *et al*: **Prevalence of chronic obstructive pulmonary disease and associated risk factors in Uganda (FRESH AIR Uganda): a prospective cross-sectional observational study**. *Lancet Glob Health* 2015, **3**(1):e44-51.
24. Vaz Fragoso CA, Van Ness PH, Murphy TE, McAvay GJ: **Spirometric impairments, cardiovascular outcomes, and noncardiovascular death in older persons**. *Respiratory Medicine* 2018, **137**:40-47.
25. Siddharthan T, Grigsby M, Miele CH, Bernabe-Ortiz A, Miranda JJ, Gilman RH, Wise RA, Porter JC, Hurst JR, Checkley W: **Prevalence and risk factors of restrictive spirometry in a cohort of Peruvian adults**. *Int J Tuberc Lung Dis* 2017, **21**(9):1062-1068.
26. Schultz K, D'Aquino LC, Soares MR, Gimenez A, Pereira CAdC: **Lung volumes and airway resistance in patients with a possible restrictive pattern on spirometry**. *Jornal Brasileiro de Pneumologia* 2016, **42**:341-347.
27. Hyatt RE, Cowl CT, Bjoraker JA, Scanlon PD: **Conditions Associated With an Abnormal Nonspecific Pattern of Pulmonary Function Tests**. *CHEST* 2009, **135**(2):419-424.
28. Aryal S, Diaz-Guzman E, Mannino DM: **COPD and gender differences: an update**. *Translational Research* 2013, **162**(4):208-218.
29. Camp PG, Goring SM: **Gender and the Diagnosis, Management, and Surveillance of Chronic Obstructive Pulmonary Disease**. *Proceedings of the American Thoracic Society* 2007, **4**(8):686-691.
30. Grønseth R, Erdal M, Tan WC, Obaseki DO, Amaral Andre FS, Gislason T, Juvekar SK, Koul PA, Studnicka M, Salvi S *et al*: **Unemployment in chronic airflow obstruction around the world: results from the BOLD study**. *European Respiratory Journal* 2017, **50**(3):1700499.
31. Montes de Oca M, Halbert RJ, Talamo C, Perez-Padilla R, Lopez MV, Muiño A, Jardim JRB, Valdivia G, Pertuzé J, Moreno D *et al*: **Paid employment in subjects with and without chronic obstructive pulmonary disease in five Latin American cities: the PLATINO study**. *The International Journal of Tuberculosis and Lung Disease* 2011, **15**(9):1259-1264.
32. Murphy JD, Liu B, Parascandola M: **Smoking and HIV in Sub-Saharan Africa: A 25-Country Analysis of the Demographic Health Surveys**. *Nicotine & Tobacco Research* 2018, **21**(8):1093-1102.
33. Nketiah-Amponsah E, Afful-Mensah G, Ampaw S: **Determinants of cigarette smoking and smoking intensity among adult males in Ghana**. *BMC Public Health* 2018, **18**(1):941.
34. Torres-Duque C, Maldonado D, Pérez-Padilla R, Ezzati M, Viegi G: **Biomass Fuels and Respiratory Diseases**. *Proceedings of the American Thoracic Society* 2008, **5**(5):577-590.
35. Silva R, Oyarzún M, Olloquequi J: **Pathogenic Mechanisms in Chronic Obstructive Pulmonary Disease Due to Biomass Smoke Exposure**. *Archivos de Bronconeumología (English Edition)* 2015, **51**(6):285-292.
36. Schermer TR, Robberts B, Crockett AJ, Thoonen BP, Lucas A, Grootens J, Smeele IJ, Thamrin C, Reddel HK: **Should the diagnosis of COPD be based on a single spirometry test?** *npj Primary Care Respiratory Medicine* 2016, **26**(1):16059.
37. Pefura-Yone EW, Balkissou AD, Poka-Mayap V, Djenabou A, Massongo M, Ofimboudem NA, Mayoh-Nguemfo CF, Tsala AG, Hadjara H, Amougou F: **Spirometric reference equations for Cameroonians aged 4 to 89 years derived using lambda, mu, sigma (LMS) method**. *BMC Pulm Med* 2021, **21**(1):344.

Please review your reference list to ensure that it is complete and correct. If you have cited papers that have been retracted, please include the rationale for doing so in the manuscript text, or remove these references and replace them with relevant current references. Any changes to the reference list should be mentioned in the rebuttal letter that accompanies your revised manuscript. If you need to cite a retracted article, indicate the article's retracted status in the References list and also include a citation and full reference for the retraction notice.
Response: We have kindly gone through all the references and they are current and appropriate. None had been retracted.

1. Areas for Improvements:
   There are no major areas for improvement. There are however, a few minor issues the authors need to tackle. These are specified below.
   1) Under "results" in "abstract" (page 2), line 9; review punctuation mark before the word "and".
   Response: We have kindly replaced the punctuation (,) with a (;) to make the two phrases distinct.

   2) Under "results" in "abstract" (page 2), line 10; replace the punctuation mark before the word "medium" with the word –"and".
   Response: We have kindly replaced the punctuation mark before the word "medium" with the word "and" as suggested.

   3) Under "study participants" in "methods" (page 4), line 6; review the location stated for Atua Government Hospital.
   Response: We have kindly corrected the location with the formal name as the the Lower Manya Krobo Municipality.

   4) Under "general characteristics of study participants" in "results" (page 7), line 4; review the phrase – "were likely to be" since you already found and know the result.
   Response: We have kindly replaced the phrase "were likely to be" with "were mostly".

   5) Under "general characteristics of study participants" in "results" (page 7), line 7; review the phrase – "were likely to be" since you already found and know the result.
   Response: We have kindly replaced the phrase "were likely to be" with "were mostly".

   6) Under "general characteristics of study participants" in "results" (page 7), line 7; indicate the proportion allocated to the underweights.
   Response: We have kindly indicated the proportion of underweights in the study to be 9.1%

   7) Under "general characteristics of study participants" in "results" (page 9), Table 1; indicate with Greek symbols, groups within which significant differences exist in the "obstructive" results under "abnormal spirometry by LLN, n (%)"
   Response: We have kindly used asterisks to indicate group difference in abnormal spirometry as were done for other parameters.

8) Under “general characteristics of study participants” in “results” (page 9), Table 1; indicate with Greek symbols, groups within which significant differences exist in results for “Respiratory symptoms, n (%)”
Response: We have kindly used asterisks to indicate group difference in abnormal spirometry as were done for other parameters.

9) Under “Respiratory symptoms” in “results” (page 9), Fig 1; indicate where in the document this “fig 1” could be located as actual figure is omitted.
Response: We have kindly indicated the location in the manuscript that Fig 1 should be inserted at Page 9 line 22. Also, Fig 1 was uploaded separately as a file document as recommended by PLOS One submission guidelines.

10) Under “Association between respiratory symptoms and abnormal spirometric patterns” in “results” (page 13), Fig 2; indicate where in the document this “Fig 2” could be located as actual figure is omitted.
Response: We have kindly indicated the location in the manuscript that Fig 2 should be inserted at Page 14 line 14. Also, Fig 2 was uploaded separately as a file document as recommended by PLOS One submission guidelines.

11) Under “Association between respiratory symptoms and abnormal spirometric patterns” in “results” (page 13), Fig 3; indicate where in the document this “Fig 3” could be located as actual figure is omitted.
Response: We have kindly indicated the location in the manuscript that Fig 3 should be inserted at Page 14 line 16. Also, Fig 3 was uploaded separately as a file document as recommended by PLOS One submission guidelines.

12) Under “Abnormal spirometric patterns” in “discussion” (page 14), line 1; do you intend to say higher or high and in reference to which of the three groups of study?
Response: We have kindly revised the statement as “the prevalence of OSP was higher in both cART-treated and cART-naïve HIV patient groups compared to non-HIV control”.

13) Under “Limitations of study” (page 18), line 17; correct the word- “ad” before the wors –“this”.
Response: We have kindly corrected the word “ad” to “and”.

14) Under “Conclusion” in “discussion” (page 18), line 2;- use the superlative of the word –“high”.
Response: We have kindly revised the statement as “the prevalence of OSP was higher in both cART-treated and cART-naïve HIV patient groups compared to non-HIV control”.
