# Supplementary material for: Abnormal spirometric patterns and respiratory symptoms in HIV patients with no recent pulmonary infection in a periurban hospital in Ghana
Source: PLoS One. 2024 Oct 16;19(10):e0273063. doi: 10.1371/journal.pone.0273063 (PMC11482697; doi:10.1371/journal.pone.0273063)
Supplement: Attachment — Submitted filename: Manuscript - PONE-D-22-21464R1.docx [file pone.0273063.s003.docx]

Manuscript : PONE-D-22-21464R1

Title : Abnormal spirometric patterns and respiratory symptoms in HIV patients with no recent

pulmonary infection in a periurban hospital in Ghana

Comment

This version has substantively improved. However, there still is window for improvements to reach the standards of PLOS One. I have extensively edited and annotated the manuscript. I urge the authors to pay attention on my comments in the manuscript. Here, I provide a brief on those comments.

For the entire manuscript, checks for compliance the use of “We” and “I”

Abstract. See manuscript on a few suggested changes

Introduction. Some affirmations lack references

Methods. The logistic regression is misphrased. Authors are expected to rephrase the formulation of logistic regression (see p. 7)

Results. Tables 2—4: All categorical variables need a reference category (RC). Females and Unmarried are categorical variables as well

Please use “Factors associated” but “determinants” which has a “causation” connotation
