# Supplementary material for: Abnormal spirometric patterns and respiratory symptoms in HIV patients with no recent pulmonary infection in a periurban hospital in Ghana
Source: PLoS One. 2024 Oct 16;19(10):e0273063. doi: 10.1371/journal.pone.0273063 (PMC11482697; doi:10.1371/journal.pone.0273063)
Supplement: Attachment — Submitted filename: response to editor and reviewers comments.docx [file pone.0273063.s005.docx]

Please review your reference list to ensure that it is complete and correct. If you have cited papers that have been retracted, please include the rationale for doing so in the manuscript text, or remove these references and replace them with relevant current references. Any changes to the reference list should be mentioned in the rebuttal letter that accompanies your revised manuscript. If you need to cite a retracted article, indicate the article’s retracted status in the References list and also include a citation and full reference for the retraction notice.

Response: We have kindly gone through all the references and they are current and appropriate. None had been retracted.

1. Areas for Improvements:
   There are no major areas for improvement. There are however, a few minor issues the authors need to tackle. These are specified below.
   1) Under “results” in “abstract” (page 2), line 9; review punctuation mark before the word “and”.

Response: We have kindly replaced the punctuation (,) with a (;) to make the two phrases distinct.

2) Under “results” in “abstract” (page 2), line 10; replace the punctuation mark before the word “medium” with the word –“and”.
Response: We have kindly corrected the punctuation as suggested.

3) Under “study participants” in “methods” (page 4), line 6; review the location stated for Atua Government Hospital.

Response: We have kindly corrected the location with the formal name of the municipality.

4) Under “general characteristics of study participants” in “results” (page 7), line 4; review the phrase – “were likely to be” since you already found and know the result.

Response: We have kindly replaced the phrase “were likely to be” with “were mostly”.

5) Under “general characteristics of study participants” in “results” (page 7), line 7; review the phrase – “were likely to be” since you already found and know the result.
Response: We have kindly replaced the phrase “were likely to be” with “were mostly”.

6) Under “general characteristics of study participants” in “results” (page 7), line 7; indicate the proportion allocated to the underweights.

Response: We have kindly indicated the proportion of underweights in the study to be 9.1%

7) Under “general characteristics of study participants” in “results” (page 9), Table 1; indicate with Greek symbols, groups within which significant differences exist in the “obstructive” results under “abnormal spirometry by LLN, n (%)”

Response: We have kindly used asterisks to indicate group difference in abnormal spirometry as were done for other parameters.

8) Under “general characteristics of study participants” in “results” (page 9), Table 1; indicate with Greek symbols, groups within which significant differences exist in results for “Respiratory symptoms, n (%)”

Response: We have kindly used asterisks to indicate group difference in abnormal spirometry as were done for other parameters.

9) Under “Respiratory symptoms” in “results” (page 9), Fig 1; indicate where in the document this “fig 1” could be located as actual figure is omitted.

Response: Base on PLOS One submission guidelines, Figures are not supposed to be added to the main manuscript, but uploaded separately.

10) Under “Association between respiratory symptoms and abnormal spirometric patterns” in “results” (page 13), Fig 2; indicate where in the document this “Fig 2” could be located as actual figure is omitted.
Response: Base on PLOS One submission guidelines, Figures are not supposed to be added to the main manuscript, but uploaded separately.

11) Under “Association between respiratory symptoms and abnormal spirometric patterns” in “results” (page 13), Fig 3; indicate where in the document this “Fig 3” could be located as actual figure is omitted.
Response: Base on PLOS One submission guidelines, Figures are not supposed to be added to the main manuscript, but uploaded separately.

12) Under “Abnormal spirometric patterns” in “discussion” (page 14), line 1; do you intend to say higher or high and in reference to which of the three groups of study?
Response: We have kindly changed the word to higher.

13) Under “Limitations of study” (page 18), line 17; correct the word- “ad” before the wors –“this”.

Response: We have kindly corrected the word “ad” to “and”.

14) Under “Conclusion” in “discussion” (page 18), line 2;- use the superlative of the word –“high”.

Response: We have kindly changed the word to higher to correspond to the results of the study.
