# Supplementary material for: Abnormal spirometric patterns and respiratory symptoms in HIV patients with no recent pulmonary infection in a periurban hospital in Ghana
Source: PLoS One. 2024 Oct 16;19(10):e0273063. doi: 10.1371/journal.pone.0273063 (PMC11482697; doi:10.1371/journal.pone.0273063)
Supplement: Attachment — Submitted filename: Response to editor queries.docx [file pone.0273063.s008.docx]

Response to editor queries:

1. Harmonize all over the places if you want spaces before and after the sign =; <; etc

Response: We have added spaces before and after all signs as suggested.

2. This is very unclear

Response: We have clarified the statement to mean: “However, the prevalence of RSP was similar among HIV patients and non-HIV controls.”

3. COPD; previous studies suggested

Response: We have kindly changed the statement as suggested.

4. This para addresses inclusion/exclusion criteria. It should have been clearer a sub-heading "Inclusion/Exclusion" and simply list/explain these criteria.

Response: We have kindly created a subheading for inclusion and exclusion criteria

5. Table 1 should have been announced earlier before aligning findings

Response: We have mentioned the Table 1 at the beginning of the paragraph as suggested.

6. Not sure the way this Table was built align with PLoS One requirements

Response: We have ensured that the Table 1 aligns with PLOSone requirements

7. all "p" in Italics please

Response: All “p” are in italics as suggested.

8. be consistent: use being female or female gender

Response: We have kindly changed all females to female gender.

9. why different font?

Response: We have harmonized all fonts in the manuscript please.

10. Is this part of main findings or not?

Response: Yes, it is part of the main findings discussing the prevalence of abnormal spirometry, which includes obstructive/restrictive patterns.

11. References: Use same font as in the manuscript

Response: The font of the references have been changed to be the same as the manuscript.
